# Structural definition of HLA class II-presented SARS-CoV-2 epitopes reveals a mechanism to escape pre-existing CD4^+^ T cell immunity

**DOI:** 10.1016/j.celrep.2023.112827

**Published:** 2023-07-19

**Authors:** Yuan Chen, Georgina H. Mason, D. Oliver Scourfield, Alexander Greenshields-Watson, Tracey A. Haigh, Andrew K. Sewell, Heather M. Long, Awen M. Gallimore, Pierre Rizkallah, Bruce J. MacLachlan, Andrew Godkin

**Affiliations:** 1 Division of Infection and Immunity, School of Medicine, Cardiff University, Cardiff CF14 4XN, UK; 2 Systems Immunity University Research Institute, School of Medicine, Cardiff University, Cardiff CF14 4XN, UK; 3 Institute of Immunology and Immunotherapy, University of Birmingham, Birmingham B15 2TT, UK; 4 Department of Gastroenterology & Hepatology, University Hospital of Wales, Cardiff CF14 4XW, UK

**Keywords:** T cells, CD4^+^ T cells, SARS-CoV-2, coronavirus, COVID-19, HLA class II, antigen presentation, immune escape, immune memory, crystallography

## Abstract

CD4^+^ T cells recognize a broad range of peptide epitopes of severe acute respiratory syndrome coronavirus 2 (SARS-CoV-2), which contribute to immune memory and limit COVID-19 disease. We demonstrate that the immunogenicity of SARS-CoV-2 peptides, in the context of the model allotype HLA-DR1, does not correlate with their binding affinity to the HLA heterodimer. Analyzing six epitopes, some with very low binding affinity, we solve X-ray crystallographic structures of each bound to HLA-DR1. Further structural definitions reveal the precise molecular impact of viral variant mutations on epitope presentation. Omicron escaped ancestral SARS-CoV-2 immunity to two epitopes through two distinct mechanisms: (1) mutations to TCR-facing epitope positions and (2) a mechanism whereby a single amino acid substitution caused a register shift within the HLA binding groove, completely altering the peptide-HLA structure. This HLA-II-specific paradigm of immune escape highlights how CD4^+^ T cell memory is finely poised at the level of peptide-HLA-II presentation.

## Highlights

• Characterization of HLA-DR1-restricted SARS-CoV-2 peptide epitopes
• Epitope immunogenicity does not correlate with peptide affinity for HLA-DR1
• Structures of six p-HLA-DR1 complexes show that low-affinity peptides bind canonically
• BA.1 mutations evade T cells via two distinct mechanisms including register shifting

## Graphical abstract

**Figure:**
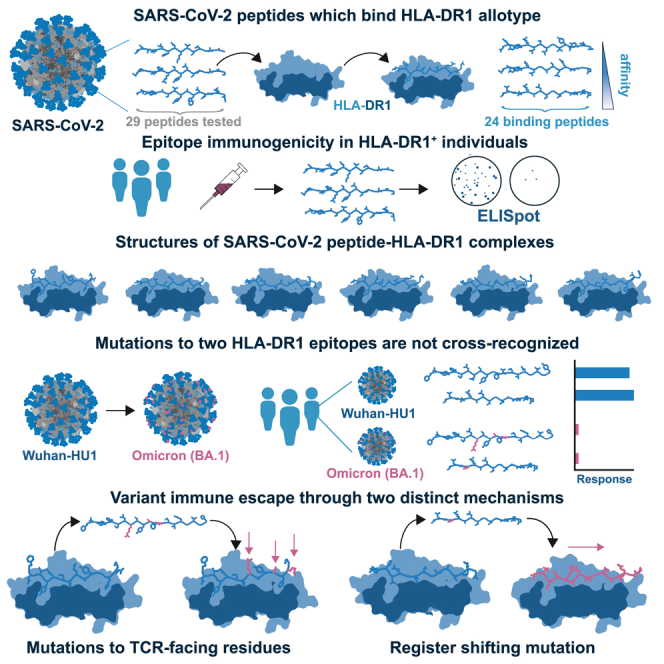

## Teaser

Chen et al. identify and characterize SARS-CoV-2-derived CD4^+^ T cell epitopes restricted by the common HLA-DR1 allotype. Biophysical analyses suggest that peptide-HLA-DR1 affinity is not the key determinant of immunogenicity, and crystallographic structures of HLA-bound variant epitopes demonstrate two different mechanisms of escape from memory CD4^+^ T cells.

## Introduction

Recovery from COVID-19, as a result of infection with the causative virus severe acute respiratory syndrome coronavirus 2 (SARS-CoV-2), is associated with a concerted immune response characterized by both CD8^+^ and CD4^+^ T cells^1^ along with neutralizing antibodies.^2^ Administration of various vaccines induces similar immune memory to the target antigen, currently the host entry protein Spike.^3^^,^^4^^,^^5^^,^^6^ Maintaining durable immunity within individuals that recognizes current and future global SARS-CoV-2 variants remains the key goal in preventing further impact caused by the circulating virus.

Highly activated CD4^+^ and CD8^+^ T cells were seen with systemic inflammation and more severe disease in patients hospitalized due to COVID-19.^7^ However, in individuals where cellular immunity was induced early after infection, the disease appeared less severe,^8^ and T cell immunity against multiple SARS-CoV-2 viral proteins is observed in nearly all patients recovered from COVID-19.^9^ SARS-CoV-2-specific central memory (CCR7^+^, CD45RA^−^) T_H_1 CD4^+^ T cells persist in patients recovered from COVID-19 with an estimated half-life of ∼200 days; T_H_2 and T_H_17 cells do not contribute significantly to this memory pool.^10^ Although there has been a clear focus on measured serological responses as markers for protective immunity, recent studies have highlighted the correlation between SARS-CoV-2-specific IFN-γ^+^ T cell responses and protection from reinfection over a 6 month follow-up period, irrespective of the levels of antibodies.^11^

Multiple groups have characterized targets of SARS-CoV-2-specific T cells, through computational prediction,^12^^,^^13^ experimental peptide mapping,^9^^,^^14^^,^^15^^,^^16^^,^^17^ humanized murine models,^18^ and library display approaches.^19^ These have revealed cellular immunity across the breadth of the SARS-CoV-2 proteome. Furthermore, in virus-inexperienced early-pandemic cohorts, pre-existing immunity to SARS-CoV-2^14^ has been attributed to cross-reactivity with endemic seasonal human betacoronaviruses, particularly to more conserved regions of the Spike protein (S2 domain)^20^ and conserved proteins of the replication-transcription complex (RTC).^21^

Despite comprehensive whole-viral-proteome analysis of both CD4^+^ and CD8^+^ T cell epitopes of SARS-CoV-2, detailed analyses of specific epitopes, particularly those presented on the human leukocyte antigen class II (HLA-II) platform to CD4^+^ T cells, are limited. Focused structural and biophysical analyses of individual CD4^+^ T cell epitopes in other anti-viral immune responses, such as influenza A^22^^,^^23^ and HIV,^24^ contribute enormously to understanding the immune response. Moreover, such data have provided mechanistic understanding to viral escape—particularly in the CD8^+^ T cell/HLA-I axis—including escape from SARS-CoV-2^25^^,^^26^^,^^27^^,^^28^ and HIV.^29^^,^^30^ Given the different properties of HLA-II-peptide binding, the mechanisms of escape may not necessarily be translatable from their cytotoxic T cell counterparts. Characteristic of the open HLA-II binding groove, peptides presented to CD4^+^ T cells exhibit a varied longer length (∼12–20 amino acids)^31^ and are anchored by a central nonamer core binding region, leaving peptide flanking residues (PFRs) at the N and C termini of the core, which may also impact immunogenicity.^32^ As a result, accurately pinpointing how epitopes are presented as peptide cargo is challenging both experimentally and computationally, especially with respect to determining the core “register” of presentation.

Utilizing iterative peptide/HLA-II display libraries covering the SARS-CoV-2 genome, Obermair et al. identified binding ligands for a series of HLA types.^19^ This approach allowed an exploration of the impact of mutants on ligand/epitope display. It is known that a mutation to either a key “downward-facing” residue that contributes to peptide binding to the HLA groove or an “upward-facing” amino acid side chain that engages the T cell receptor (TCR) can lead to loss of T cell activation.^33^ For instance, in HIV, escape from CD4^+^ T cell recognition of immunodominant viral epitopes has been demonstrated via mutations that disrupt TCR recognition while maintaining HLA-II binding.^34^ Although not clearly proven, it is also plausible that mutations in epitopes might create new ligand binding registers, hence theoretically shifting the TCR upward-facing residues.^19^ Structural explanations of such molecular mechanisms, however, are yet to be described in the CD4^+^ T cell/HLA-II axis.

In this study, we set out at the start of the pandemic to identify and characterize peptide epitopes of SARS-CoV-2 that could be used to explore the importance of CD4^+^ T cell responses in anti-viral immunity. Using HLA-DRA1^∗^0101, B1^∗^0101 (HLA-DR1) as a model allotype, we first assessed the ability of 29 SARS-CoV-2-derived candidate peptides to bind HLA-DR1 and characterized their immunogenicity *in vitro* using blood from a range of HLA-DR1-positive and -negative donors. Details of these CD4^+^ T cell responses would allow insight into both epitope selection and immunodominance and the mechanism(s) behind loss of T cell responses that independently associate with recurrent infection. We describe the structures of six epitopes enabling unequivocal definition of epitope core region and register. As the pandemic unfolded and variant viruses emerged, we analyzed their effect on bound peptide cargo. Corroborating further structural data of Omicron variant-mutated peptide-HLA-DR1 complexes with *in vitro* binding and immunogenicity data, we identify distinct mechanisms of immune escape from T cell recognition by Omicron variant peptides, highlighting the importance of the peptide-HLA-II platform characteristics in maintaining long-lasting anti-viral immunity.

## Results

### Identification of HLA-DR1 binding peptides from SARS-CoV-2

**Figure 1:**
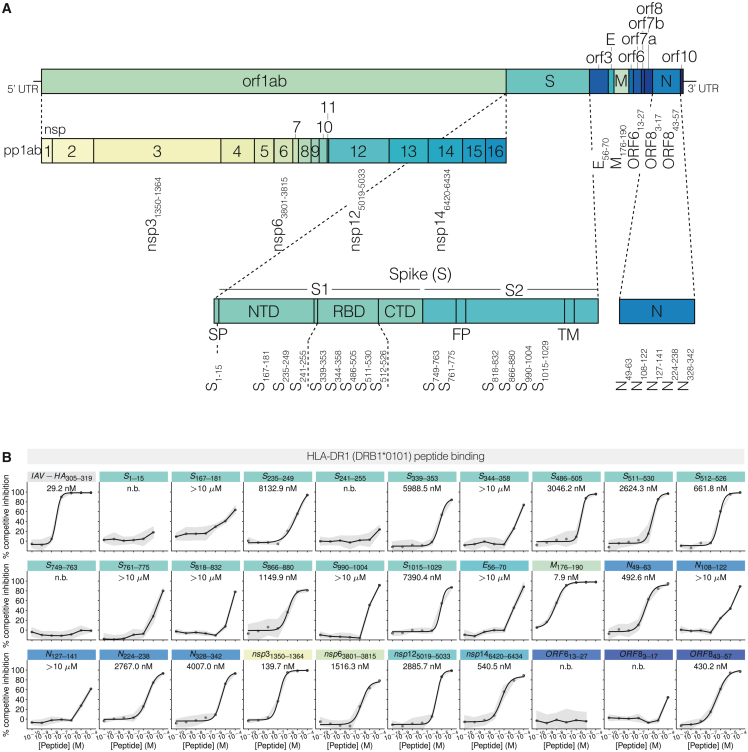
Identification of HLA-DR1 binding peptides derived from SARS-CoV-2 (A) A schematic overview of the SARS-CoV-2 genome highlighting peptides selected for analysis. An expanded view of Spike and the non-structural proteins produced from orf1ab (nsp1 to nsp16) is shown. S, Spike; E, Envelope; M, Membrane; N, Nucleocapsid; orf, open reading frame; nsp, non-structural protein; and UTR, untranslated region. Within Spike: SP, signal peptide; NTD, N-terminal domain; RBD, receptor binding domain; CTD, C-terminal domain; FP, fusion peptide; and TM, transmembrane domain. (B) Peptide-HLA-DR1 binding curves for each peptide as determined by competitive inhibition assay. HA_305–319_ was used as a comparative control. Data representative of n = 3 independent experiments, each with n = 3 technical replicates. Data presented as mean percentage inhibition (circles) with standard deviation (shaded area) shown as error from n = 3 technical replicates. An IC_50_ value defining peptide-HLA-DR1 binding affinity (inset) is shown where fitting resulted in values of <10 μM (curve fit, black line). Peptides with poor curve fit and weak binding (>10 μM) or no binding (n.b.) have mean values that are connected via straight lines.

To characterize important HLA-II peptide epitopes of SARS-CoV-2, we aimed to provide a mechanistic understanding of their immunogenicity through the structural characterization of their HLA-II presentation using HLA-DR1 as a model allotype. Surveying the literature on CD4^+^ T cell epitopes in either individuals who were unexposed or patients recovered/recovering from COVID-19 circa November 2020, we selected 29 peptides using evidence of immunogenicity in cohorts containing HLA-DR1 positivity combined with predicted binding to HLA-DR1 according to NetMHCIIpan^35^ (Table 1). Selected candidate peptides encompassed diverse regions across the SARS-CoV-2 (Wuhan HU-1) genome, including both Spike (S)-derived and non-Spike peptides (Figure 1A). Twenty-seven peptides were synthesized as 15mers, registering the predicted 9mer core binding sequence centrally, flanked by 3-amino-acid N-terminal and C-terminal PFRs. Two further peptides were identified as HLA-DR1 epitopes through the isolation and HLA-II restriction of CD4^+^ T cell clones, which were reactive to 20mer peptides,^36^ and, as core prediction was not conclusive, S_486–505_ and S_511–530_ were synthesized as 20mer peptides.

**Table 1:** SARS-CoV-2-derived peptides selected for analysis

Name	Sequence	Protein	Start	End	Length	Reference
S_1–15_	MFVFLVLLPLVSSQC	S	1	15	15	Prakash et al.^18^
S_167–181_	TFEYVSQPFLMDLEG	S	167	181	15	Peng et al.^15^
S_235–249_	ITRFQTLLALHRSYL	S	235	249	15	Mateus et al. and Nelde et al.^14^^,^^16^
S_241–255_	LLALHRSYLTPGDSS	S	241	255	15	Mateus et al.^14^
S_339–353_	GEVFNATRFASVYAW	S	339	353	15	Mateus et al.^14^
S_344–358_	ATRFASVYAWNRKRI	S	344	358	15	Mateus et al.^14^
S_486–505_	FNCYFPLQSYGFQPTNGVGY	S	486	505	20	Tye et al.^36^
S_511–530_	VVLSFELLHAPATVCGPKKS	S	511	530	20	Tye et al.^36^
S_512–526_	VLSFELLHAPATVCG	S	512	526	15	Peng et al.^15^
S_749–763_	CSNLLLQYGSFCTQL	S	749	763	15	Peng et al.^15^
S_761–775_	TQLNRALTGIAVEQD	S	761	775	15	Mateus et al.^14^
S_818–832_	IEDLLFNKVTLADAG	S	818	832	15	Mateus et al.^14^
S_866–880_	TDEMIAQYTSALLAG	S	866	880	15	Peng et al.^15^
S_990–1004_	EVQIDRLITGRLQSL	S	990	1,004	15	Mateus et al.^14^
S_1015–1029_	AAEIRASANLAATKM	S	1,015	1,029	15	Peng et al.^15^
E_56–70_	FYVYSRVKNLNSSRV	E	56	70	15	Peng et al. and Nelde et al.^15^^,^^16^
M_176–190_	LSYYKLGASQRVAGD	M	176	190	15	Peng et al., Nelde et al., and Prakash et al.^15^^,^^16^^,^^18^
N_49–63_	TASWFTALTQHGKED	N	49	63	15	Nelde et al.^16^
N_108–122_	WYFYYLGTGPEAGLP	N	108	122	15	Nelde et al.^16^
N_127–141_	KDGIIWVATEGALNT	N	127	141	15	Nelde et al.^16^
N_224–238_	LDRLNQLESKMSGKG	N	224	238	15	Nelde et al.^16^
N_328–342_	GTWLTYTGAIKLDDK	N	328	342	15	Nelde et al.^16^
nsp3_1350–1364_	KSAFYILPSIISNEK	orf1ab (nsp3)	1,350	1,364	15	Prakash et al.^18^
nsp6_3801–3815_	NRYFRLTLGVYDYLV	orf1ab (nsp6)	3,801	3,815	15	Mateus et al.^14^
nsp12_5019–5033_	PNMLRIMASLVLARK	orf1ab (nsp12)	5,019	5,033	15	Prakash et al.^18^
nsp14_6420–6434_	LDAYNMMISAGFSLW	orf1ab (nsp14)	6,420	6,434	15	Prakash et al.^18^
ORF6_13–27_	EILLIIMRTFKVSIW	ORF6	13	27	15	Prakash et al.^18^
ORF8_3–17_	FLVFLGIITTVAAFH	ORF8	3	17	15	Prakash et al.^18^
ORF8_43–57_	SKWYIRVGARKSAPL	ORF8	43	57	15	Nelde et al.^16^

Synthesized peptides were first assayed *in vitro* for the ability to bind HLA-DR1 by competitive inhibition assays.^37^ The immunodominant HLA-DR1-restricted influenza A virus hemagglutinin (HA) peptide HA_305–319_^38^^,^^39^ was also tested as a comparator, giving an IC_50_ of 29 nM. The binding assays of the SARS-CoV-2 peptides gave rise to a wide range of measured affinities, which were divided into four groups: 6 strong binding peptides (IC_50_ < 1,000 nM), 10 weak binding peptides (1,000 < IC_50_ < 10,000 nM), 8 very weak but detectable binding peptides (IC_50_ > 10,000 nM or 10 μM), and 5 peptides with no detectable binding (despite *in silico* predictions) (Figure 1B). The Membrane (M)-derived peptide M_176–190_ had the highest affinity (IC_50_ = 7.9 nM) and was the only binder stronger than our reference epitope (HA_305–319_, IC_50_ = 29 nM). Of the six peptides considered strong binders, only one was Spike derived (S_512–526_, IC_50_ = 662 nM). Thus, 24 of the 29 peptides tested bound HLA-DR1 and encompassed a wide range of affinities (IC_50_ = 7.9 to >10,000 nM).

### Immunogenicity of HLA-DR1 binding peptides from SARS-CoV-2

**Figure 2:**
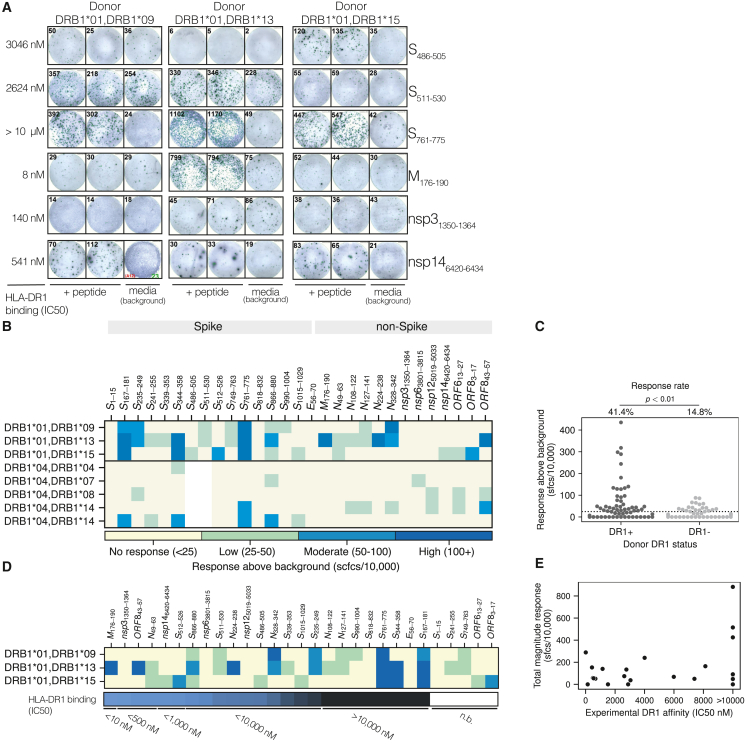
Immunogenicity of selected SARS-CoV-2 peptides in HLA-DR1^+^ and HLA-DR4^+^ donors (A) Peptide-specific, 12-day-expanded T cell responses to SARS-CoV-2 peptides. Example test peptide (n = 2) and background (media) control IFN-γ ELISpot assays for the HLA-DR1^+^ donors (n = 3) are shown. Examples shown are of peptides selected for later structural analyses. (B) Heatmap summary of IFN-γ ELISpot data. Donors are grouped by HLA-DR1^+^ (n = 3) and HLA-DR4^+^ (n = 5). Each peptide was tested twice (n = 2) in each donor. ELISpot assays were performed in duplicate (n = 2). The maximal response from either time point is shown, with individual time points shown in Figures S1A and S1B. Responses were background subtracted (medium only), normalized to sfcs/10,000 cells, and binned into low, moderate, and high responders (cutoffs and colors indicated at bottom). S_486–505_ and S_511–530_ were assayed in HLA-DR1^+^ donors (n = 3) only and post-vaccination only. (C) Summary of maximal response of all peptides (n = 29) in all donors tested (n = 8), divided into donor DR1 status. Each marker represents a single donor maximal response to a single peptide (n = 2 ELISpot wells). Dashed line at 25 sfcs/10,000 cells was used as a cutoff for donor-peptide response. Response rate is shown as a percentage. Inset p value calculated via Fisher's exact test comparing DR1^+^/DR4^+^ status (groups) and the positive responses to peptides (outcomes). (D) Heatmap summary of IFN-γ ELISpot data presented in (B) but ordered by HLA-DR1 binding affinity (IC_50_) as determined in Figure 1B: strongest affinity (left) to weakest (right). HLA-DR1^+^ donor data only are shown. (E) Scatterplot summary of total magnitude response to each peptide (summated maximal response by each donor for each peptide) in HLA-DR1^+^ donors (n = 3) against HLA-DR1 binding affinity (IC_50_) as determined in Figure 1B. No correlation was observed.

To focus our analyses, the immunogenicity of each candidate peptide was assessed in short-term T cell lines derived from HLA-DR1-positive or -negative donors, whereby isolated peripheral blood mononuclear cells (PBMCs) underwent a brief 12 day culture with individual candidate peptides. Peptide-specific T cell expansion to each was assessed via overnight IFN-γ ELISpot in response to peptide restimulation (Figures 2A and 2B). Each donor was assayed for reactivity at two time points, pre- (Figure S1A) and post- (Figure S1B) vaccination, and the maximal response is summarized in Figure 2B. Peptides S_486–505_ and S_511–530_ were identified in a separate cohort of health-care workers^36^ after the vaccination of the HLA-DR1-positive cohort and thus tested post-vaccination only.

The validity of the initial peptide selection was confirmed by the significant increase in the total number of T cell responses in HLA-DR1-positive donors (Figure 2C) (HLA-DR1-positive donors 36/87 (41%) vs. HLA-DR1-negative donors 20/135 (15%), p < 0.01, Fisher’s exact test). Across the panel of tested peptides, response rate in HLA-DR1-positive donors to Spike peptides was higher at 23/45 (51%) than for non-Spike peptides, with 13/42 (31%), possibly indicative of vaccination. As expected, not only was the overall response rate higher in HLA-DR1-positive donors, but larger magnitude cultured responses (>100 spot-forming cells [sfcs]/10,000 cultured cells) were confined to HLA-DR1-positive donors (Figure 2C).

S_167–181_, S_761–775_, and S_866–880_ were recognized by all HLA-DR1-positive donors and may represent immunodominant Spike epitopes. S_167–181_ and S_761–775_ induced the strongest T cell expansions (Figure 2B), yet curiously, both of these peptides were designated as very weak binders to HLA-DR1 (IC_50_ > 10,000 nM; Figures 1B and 2D). The S_866–880_ peptide bound with a higher affinity to HLA-DR1 (weak binder, IC_50_ = 1,149.9 nM; Figures 1B and 2D), yet the magnitude of expanded response did not exceed S_761–775_. Within the tested peptides, peptide-HLA-DR1 binding affinity did not correlate with the magnitude of T cell expansion to candidate peptides (Figure 2E). There was also no correlation between percentage eluted ligand (Rank-EL) (Figure S1C) or predicted affinity (Figure S1D) and total magnitude of cultured response, highlighting how a prediction-based approach may miss important epitopes in this context.

To confirm HLA-DR1 presentation of the peptides, we generated a CD4^+^ T cell-enriched (magnetically sorted) T cell line and used T2 cells lentivirally transduced with HLA-DR1 (DR1^+^ only).^22^ These were used as antigen-presenting cells (APCs) to stimulate cognate CD4^+^ T cell lines, which confirmed that the very weak peptide binders, such as S_761–775_, could be presented by HLA-DR1 in the context of these enriched, more protracted T cell cultures (Figures S1E and S1F). One donor (HLA-DRB1^∗^01, -DRB1^∗^13) responded to 50% (7/14) of the non-Spike peptides. This individual was the only donor (either HLA-DR1-positive or -negative) to respond to M_176–190_, yet exhibited the strongest response measured from any donor to a non-Spike peptide. M_176–190_ was the highest-affinity HLA-DR1 binder we measured from the SARS-CoV-2 peptides (Figure 1B). The HLA-DR1 restriction was again confirmed using the T2-DR1^+^-only cells, where an enriched CD4^+^ T cell line (which had undergone three rounds of enrichment/stimulation) exhibited ∼80% M_176–190_ reactivity by dual expression of TNF-α and IFN-γ via intracellular cytokine staining (Figure S1G). Together, these results highlight the complexity of coupling HLA-peptide binding affinities, T cell responses, and immunodominance and predicting ligand → epitope selection for HLA-II.

### Structural definition of SARS-CoV-2 epitopes presented by HLA-DR1

**Figure 3:**
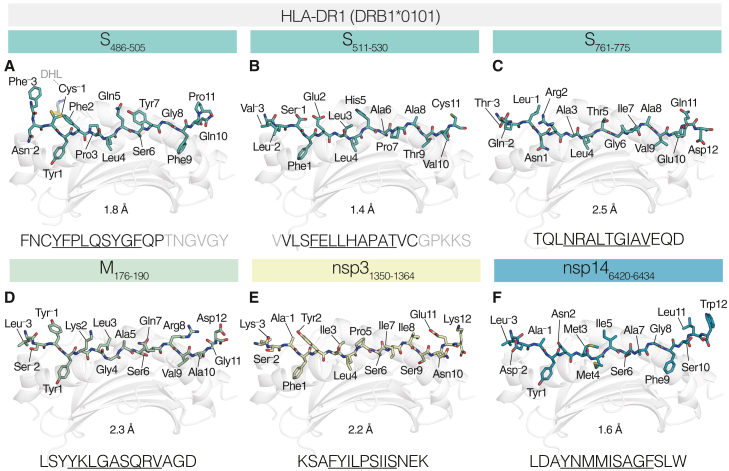
Structural definition of SARS-CoV-2-derived HLA-DR1 epitopes Structural overview of HLA-DR1-S_486–505_ (A), -S_511–530_ (B), -S_761–775_ (C), -M_176–190_ (D), -nsp3_1350–1364_ (E), and -nsp14_6420–6434_ (F). In each, the HLA-DR1 peptide binding groove (light gray, cartoon representation) and bound peptide cargo are shown. Each peptide is shown as sticks and colored by atom (C matches the color of the viral protein origin shown at the top; N, blue; O, red; S, yellow). Residues within the peptides are numbered according to their register position, i.e., Tyr1 is in the P1 position, and Phe^−^3 is in the P^−^3 position. Inset peptide amino acid sequences are shown below each, with modeled residues (black) and unmodeled residues (gray) indicated, and the core nonamer binding register is underlined. Resolution is indicated below each.

Of the 29 peptides studied, we focused our structural analysis on three Spike and three non-Spike peptides, reasoning that this approach would characterize epitopes relevant to vaccination/infection (Spike) and infection only (non-Spike). A rationale for the selection of each is given below. All six peptide-HLA-DR1 complexes were solved via X-ray crystallography at resolutions of 1.4 to 2.5 Å (Figure 3; data collection and refinement statistics detailed in Table S1). The primary aim was to define the core binding motif of each peptide, which determines the presented peptide’s binding register. We used unbiased omit map analysis to affirm binding registers (Figure S2). To consider each complex in the context of HLA-DR1 binding “fit,” below we compare each to eluted ligand (EL) enrichment or binding affinity (BA) motifs described by NetMHCIIpan-4.1 data.^35^ Concurrently, the definition of the core binding motif allowed for the assignment of outward-facing epitope positions, which are typically contacted by TCRs.^40^^,^^41^

### S_486–505_: FNCYFPLQSYGFQPTNGVGY

S_486–505_-reactive CD4^+^ T cell clones restricted to HLA-DR1 have been isolated from individuals recovering from COVID-19 by ourselves^36^ and others.^42^ S_486–505_ bound HLA-DR1 at low affinity (IC_50_ = 3,046 nM; Figures 1B and 2D). The refolded complex was crystallized and diffracted to a resolution of 1.8 Å. HLA-DR1-S_486–505_ was solved in space group P 2_1_ 2_1_ 2 containing a single copy in the asymmetric unit (Figure 3A). HLA-DR1 bound S_486–505_ via the register FNCYFPLQSYGFQPTNGVGY (core residues underlined). Binding in this register satisfied 3/4 anchor residue preferences for HLA-DR1, incorporating the favored P1-Tyr, P4-Leu, and P6-Ser. Deviation from the motif ideals was observed at P9 by incorporation of P9-Phe, which prefers shorter aliphatic residues. Within the core, this register placed P2-Phe, P5-Gln, and P7-Tyr as potential contact points for TCR recognition.

A free cysteine within the peptide did not negatively affect HLA-DR1-S_486–505_ refolding; however, a strong positive peak in the Fo-Fc map at the P^−^1-Cys side chain indicated additional density capping the reactive free thiol. A covalently conjugated cystamine, likely from reaction with cysteamine (a component of the refolding solution), modeled and refined well into this density. The cysteamine, however, did not affect peptide-HLA-DR complexation due to its outward-facing orientation.

### S_511–530_: VVLSFELLHAPATVCGPKKS

Studies using peptides encompassing S_511–530_ have identified the region to contain a potential CD4^+^ T cell epitope in cohorts of individuals recovered from COVID-19 (3%–22% of individuals responded).^15^^,^^17^^,^^43^ We have recently isolated CD4^+^ T cell clones reactive to S_511–530_ and confirmed HLA-DR1 restriction via activation to HLA-matched allogeneic lymphoblastoid cell lines (LCLs).^36^ CD4^+^ T cell clones restricted to HLA-DR1 have also been identified by others.^42^^,^^44^ S_511–530_ was designated a weak binder (IC_50_ = 2,624 nM; Figure 1B). The structure of HLA-DR1-S_511–530_ was solved to 1.4 Å resolution in space group P 6_5_ 2 2 (Figure 3B).

With a defined core of VVLSFELLHAPATVCGPKKS, S_511–530_ best satisfied the HLA-DR1 binding motif within all presented peptide-HLA-DR1 complexes, where P1-Phe, P4-Leu, and P6-Ala are all the most enriched amino acids at these positions within both the EL and the BA motif. At P9, P9-Thr is enriched in the BA motif but not EL data. Despite satisfying BA/EL motifs, S_511–530_ exhibited a modest affinity for HLA-DR1 (∼2,600 nM). The presence of Cys11 in the C-terminal PFR did not affect refold or crystallization ability, and additional density indicating thiol capping was not present as observed in HLA-DR1-S_486–505_. Within the core epitope, S_511–530_ exhibits diverse physicochemical properties at TCR-facing positions: acidic P2-Glu, a short aliphatic P6-Ala, P7-Pro and P8-Ala stretch, and the basic P5-His, which shrouded over P3-Leu to form a central charge within the epitope core.

### S_761–775_: TQLNRALTGIAVEQD

S_761–775_ was an immunogenic CD4^+^ T cell epitope in healthy unexposed individuals (3/14; 21%), of which 5/14 donors possessed the DRB1^∗^0101 allele.^14^ More recently, S_761–775_ was identified as the most immunogenic peptide to induce CD4^+^ T cell reactivity *ex vivo*, including in subjects with no obvious history of virus exposure.^45^ Despite very weak binding to HLA-DR1 *in vitro* (IC_50_ > 10 μM; Figure 1B), not only were T cell responses to S_761–775_ observed in all three of our DR1^+^ donors (Figures 2A and 2B), but the peptide bound and was crystallized with HLA-DR1. The structure of DR1-S_761–775_ was solved to 2.5 Å resolution in space group C 2 2 2_1_ containing three copies in the asymmetric unit (Figure 3C).

S_761–775_ bound HLA-DR1 via the register TQLNRALTGIAVEQD, which placed an unfavorable Asn at P1 while placing favorable residues at all other anchor positions: P4-Leu, P6-Gly, and P9-Val. Thus, lower compatibility of P1-Asn for the P1 pocket, with preferences for larger bulky side chains, was the most likely contributor to the observed weak affinity of S_761–775_ for HLA-DR1 (weakest of all crystallized peptide-HLA-DR1 complexes) when considering binding motifs. Outward-facing residues across the core and PFRs were very diverse (polar, aliphatic, and charged).

### M_176–190_: LSYYKLGASQRVAGD

M_176–190_ was initially identified in convalescent (21/22 individuals; 95%)^16^ and fully recovered (16/34 individuals)^15^ patient cohorts. M_176–190_ has confirmed restriction to DRB1^∗^0101 in humanized mice,^18^ using DRB1^∗^0101-expressing APCs^46^ and via HLA-DR1-M_176–190_ tetramers.^47^ M_176–190_ is therefore one of the best-characterized CD4^+^ T cell epitopes for SARS-CoV-2 in the Immune Epitope Database (IEDB). In our study, M_176–190_ had the highest affinity for HLA-DR1 assayed (IC_50_ = 8 nM; Figure 1B). The structure of HLA-DR1-M_176–190_ was solved at 2.3 Å resolution in space group C 2 2 2_1_ with three copies in the asymmetric unit (Figure 3D).

HLA-DR1 bound M_176–190_ via the register LSYYKLGASQRVAGD, which is in line with previous *in vitro* data determining a minimal epitope for reactive CD4^+^ T cells.^46^^,^^47^ This register placed the archetypal aromatic P1-Tyr into the P1 pocket as well as the favored P6-Ser and P9-Val. P4-Gly represented the only mismatch from mooted motif ideals. Outward TCR-facing residues were most varied at the N terminus (P^−^1-Try and P2-Lys) and the C terminus (P8-Arg). In contrast, the aliphatic central core (P4-Gly and P5-Ala) maintained a low profile in the groove. Despite being the highest-affinity binder, M_176–190_ did not distinguish itself by best satisfying EL/BA motifs compared with other epitopes with lower affinity (particularly S_511–530_).

### nsp3_1350–1364_: KSAFYILPSIISNEK

nsp3_1350–1364_ was identified as immunogenic in humanized HLA-DR1^+^ transgenic mice and elicited strong IFN-γ responses in individuals who were both SARS-CoV-2 experienced and HLA-DR1^+^ (27/30; 90%), as well as healthy donors with no known exposure (10/10; 100% deemed responders).^18^ As one of the highest-affinity binders for HLA-DR1 identified herein (IC_50_ = 140 nM; Figure 1B) the structure of nsp3_1350–1364_ was solved at 2.2 Å resolution in space group P 2_1_ 2_1_ 2_1_ with two copies in the asymmetric unit (Figure 3E).

HLA-DR1 bound nsp3_1350–1364_ via its predicted register (KSAFYILPSIISNEK). Placing P1-Phe and P4-Leu (most enriched in EL and BA motifs) and P6-Ser (enriched at P6) meant that nsp3_1350–1364_ satisfied 3/4 positions within the HLA-DR1 binding motif. Incorporation of P9-Ser is deemed detrimental to HLA-DR1 binding. Thus, P9-Ser is likely the only shortcoming to nsp3_1350–1364_ affinity when considering the core binding motif. Yet, nsp3_1350–1364_ was still one of the highest-binding peptides, suggesting P9 may not be as important to HLA-DR1 binding affinity compared with other pockets (such as P1). In TCR-facing positions, nsp3_1350–1364_ placed the large bulky Tyr residue at position P2 (P2-Tyr), forming a pi-pi stack between aromatic rings of P2-Tyr and His50β, resulting in a protruding surface at this TCR-facing position.

### nsp14_6420–6434_: LDAYNMMISAGFSLW

nsp14_6420–6434_ was identified as an HLA-DR1 peptide epitope in HLA-DR1^+^ transgenic mice and HLA-DR1^+^ exposed individuals (16/30; 53%) and individuals with no known exposure (3/10; 30%).^18^ Exhibiting strong binding to HLA-DR1 (IC_50_ = 541 nM; Figure 1B), the structure of nsp14_6420–6434_ was solved at 1.6 Å resolution in space group P 6_5_ 2 2 with a single copy in the asymmetric unit (Figure 3F).

HLA-DR1 bound nsp14_6420–6434_ via its predicted register (LDAYNMMISAGFSLW). Once again, nsp14_6420–6434_ satisfied 3/4 of the allowed HLA-DR1 pocket anchors: P1-Tyr, P4-Met, and P6-Ser, while P9-Phe represented a deviation. The incorporation of P9-Phe was reminiscent of the similar S_486–505_ (also P9-Phe), which bound HLA-DR1 at weak affinity. TCR-facing residues were highly aliphatic (P^−^1-Ala, P3-Met, P5-Ile, P7-Ala, P8-Gly, and P11-Leu), the exception being the polar P2-Asn.

In summary, each peptide bound HLA-DR1 canonically in the “forward” orientation that is described for HLA-DR,^48^ excluding CLIP.^49^ We observed little deviation in the overall structure of HLA-DR1 irrespective of bound peptide (Figure S3A), particularly within the peptide binding groove (Figure S3B), where the solved diversity of bound peptides had little impact on the position of the HLA-DR1 residues that lined each pocket (Figure S3C). NetMHCIIpan-4.1 correctly predicted five of six peptide registers, the exception being S_486–505_ (prediction, FNCYFPLQSYGFQPTNGVGY, core reliability score 89% vs. structure, FNCYFPLQSYGFQPTNGVGY). The bound peptides encompassed a range of affinities (IC_50_ = 7.9 to >10,000 nM), and no peptide fully satisfied the EL or BA motif. HLA-DR1-S_511–530_ presented a “best fit,” deviating only by Thr incorporation at P9. Despite this, S_511–530_ was in fact a weak binding peptide *in vitro*. The strongest binding peptide, M_176–190_, also satisfied 3/4 binding pocket preferences: incorporating its mismatch at position P4 (Gly). In fact, satisfying 3/4 EL binding pocket preferences was a commonality to all six complexes. The weakest affinity, S_761–775_ (IC_50_ > 10,000 nM), was the only complex where the P1 position did not incorporate a favored aromatic side chain, which may point toward an increased importance of peptide-HLA-DR1 affinity compared with other pockets. Nevertheless, the degree of adherence to core binding motifs did not clearly distinguish strong from weak binders, suggesting more subtle parameters may determine binding affinity such as the influence of PFRs.^50^

### Spike-derived SARS-CoV-2 HLA-DR1 epitopes have mutated in viral variants

The impact of emerging viral mutations of SARS-CoV-2 on immunity has mainly been assessed in the context of escape from Spike-specific neutralizing antibodies.^51^^,^^52^^,^^53^^,^^54^ However, recent data have demonstrated the loss of T cell responses as crucial to a decline in immunity from reinfection.^11^ This has not been explored mechanistically, particularly for HLA-II-restricted responses, which may be critical for prolonged serological protection.

The defined HLA-DR1 epitopes we initially studied used the Wuhan HU-1 sequences (Figure 1A and Table 1). Each of the non-Spike epitopes studied structurally remained stable and largely unmutated (to June 2022) when comparing sequenced genomes within the all-time global GISAID ncov genomic sequencing database (Figure S4A).^55^ This included sequences of WHO-designated variants of interest (VOI)/variants of concern (VOC) strains, i.e., Alpha to Omicron. nsp14_6420–6434_ exhibited a brief sequence deviation through incorporation of L6433F (maximal genotypic frequency ∼5%; circa November 2021) present in a Delta (21J) strain predominating in Australasian viral genome sequences that did not persist regionally or globally (Figures S4B–S4D).

**Figure 4:**
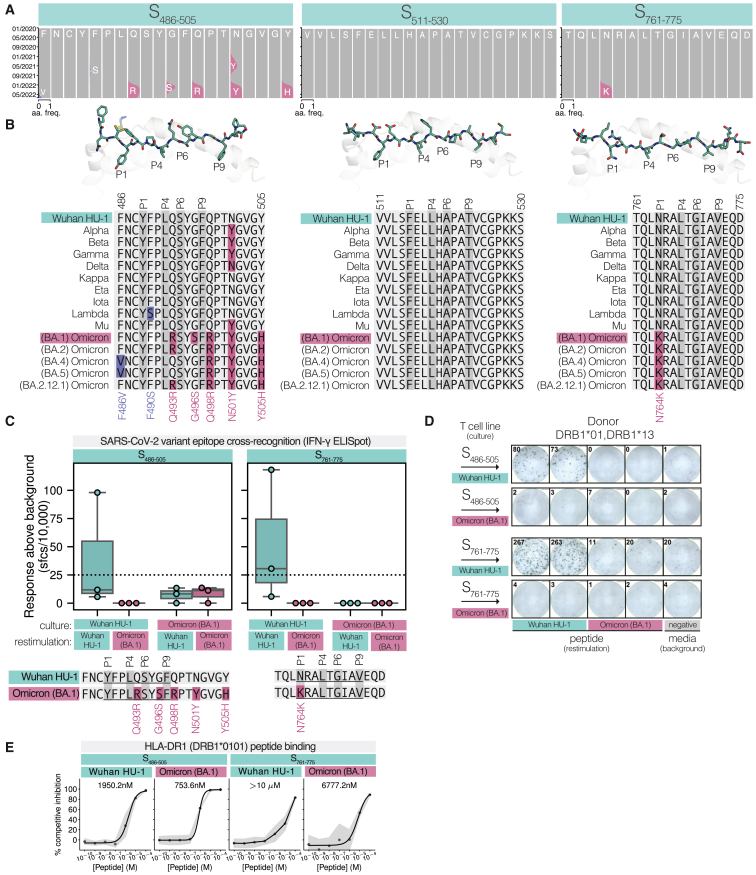
Impact of SARS-CoV-2 variants on epitope HLA-DR1 binding and immunogenicity (A) Cumulative genotypic frequency plots of amino acid usage of peptide epitope residue positions over time (January 2020–June 2022) in global viral genome sequences (GISAID)^55^: HLA-DR1-S_486–505_ (left), -S_511–530_ (middle), and -S_761–775_ (right). Reference (Wuhan HU-1 strain) amino acid usage frequency (aa. freq.) is colored gray, and mutations associated with Omicron (BA.1) mutations are colored pink. Other mutations present in other lineages are colored blue. Plots were generated using the Nextstrain ncov portal.^58^ (B) Sequence alignment of epitope sequences in SARS-CoV-2 variants. Variant mutations are as defined by the CoVariants project,^59^ highlighting mutations found in Omicron (BA.1) (pink) and other variants (blue) compared with Wuhan HU-1. Anchor residue positions are highlighted (darker gray) based on structural definitions. (C) *In vitro* immunogenicity of Wuhan HU-1 and Omicron (BA.1) variants of S_486–505_ and S_761–775_: overnight IFN-γ ELISpot (n = 2 wells) in response to short-term culture with both variant peptides in HLA-DR1^+^ donors (n = 3). Data are presented as boxplots (center line, mean; box edges, IQR; whiskers, ±1.5^∗^IQR) with individual response by each donor shown via circles. Data are colored by restimulating peptide during ELISpot assay as indicated below. (D) Representative IFN-γ ELISpot images of data described in (C) for donor DRB1^∗^01, DRB1^∗^13. Peptides used in the cultured T cell line are across rows, and the variant peptides used for restimulation (overnight ELISpot assay) are indicated in columns. ELISpot images from all three donors are shown in Figure S4E. (E) Peptide-HLA-DR1 binding curves for Omicron (BA.1) variant peptide epitopes of S_486–505_ and S_761–775_. Data are presented as described in Figure 1B with calculated affinity (IC_50_) indicated in the insets, representative of n = 3 independent assays, each with n = 3 technical replicates.

Within Spike, S_511–530_ also remained stable; however, S_486–505_ and S_761–775_ contained mutations that have persisted in sequenced viral genomes (Figure 4A). Within S_486–505_, N501Y was one of the first defining mutations to emerge^56^ circa November 2020 (Figure 4A). N501Y was present within Alpha, Beta, and Gamma, but was absent in Kappa, Eta, Iota, and Lambda before returning in Mu and all Omicron-designated variants (Figure 4B). Later mutations arose centrally to the epitope through the outgrowth of Omicron-designated strains circa November 2021. Within Omicron (BA.1), the earliest Omicron lineage,^57^ Q493R and G496S mutations occupied core residue positions P5 and P8 within the HLA-DR1 epitope. The more C-terminal Q498R (P10), N501Y (P13), and Y505H (P17) have persisted in more recent Omicron lineages (BA.2, BA.4, BA.5, and BA.2.12.1), while Q493R has reverted (Q493; BA.4 and BA.5) along with G496S (G496; BA.2, BA.4, BA.5, and BA.2.12.1). Likewise, the spread of Omicron (BA.4 and BA.5) circa May 2022 resulted in the rising frequency of a further mutation, F486V, positioned at P^−^3 in the HLA-DR1 presented epitope.

S_761–775_ was more stable in comparison, but exhibited a single mutation, N764K, located at the P1 anchor residue position with HLA-DR1-S_761–775_. N764K emerged circa November 2021 as a defining mutation of the Omicron (BA.1) variant (Figures 4A and 4B). N764K persisted throughout all Omicron-designated lineages (BA.1, BA.2, BA.4, BA.5, and BA.2.12.1) to become the dominant genotype (∼100% genotypic frequency) throughout global SARS-CoV-2 genomic sequencing data circa May 2022. Thus, the N764K mutation within S_761–775_ can be considered a pan-Omicron-lineage mutation.

### Omicron (BA.1) mutations result in a loss of recognition of two structurally defined HLA-DR1 epitopes

As Omicron (BA.1) represented the biggest step change in viral sequence to emerge, we synthesized and assessed the impact of Omicron (BA.1) mutations on the immunogenicity of S_486–505_ and S_761–775_; henceforth S_486–505_^Wuhan HU-1^ and S_761–775_^Wuhan HU-1^ or S_486–505_^Omicron (BA.1)^ and S_761–775_^Omicron (BA.1)^. We have recently shown that CD4^+^ T cell clones reactive to S_486–505_^Wuhan HU-1^ presented by HLA-DR1 were not able to cross-recognize S_486–505_^Omicron (BA.1)^ at a clonal level, even at high concentrations of peptide (10 μM).^36^ To further this analysis of S_486–505_ and to understand S_761–775_ in Omicron, we assayed Omicron (BA.1) recognition of S_486–505_ and S_761–775_ *in vitro* in our HLA-DR1-positive donors who initially responded to Wuhan HU-1-derived sequences. We assessed cross-recognition between the corresponding Wuhan HU-1 and Omicron (BA.1) peptides by generating short-term cultures (12 days) of T cells exposed independently to each peptide. The expanded T cells were subsequently assayed for responding cells via overnight stimulation with Omicron (BA.1) variant or Wuhan HU-1 peptides, measuring activation via IFN-γ ELISpot. This approach allowed for the assessment of cross-recognition by expanded T cells that may be at low frequency *ex vivo*.

We first retested the expansion of T cells stimulated with Wuhan HU-1 peptides, where one of three donors responded to S_486–505_^Wuhan HU-1^ and two of three donors responded to S_761–775_^Wuhan HU-1^ (Figures 4C, 4D, and S3E). These same T cell lines exhibited no reactivity to the variant peptides, S_486–505_^Omicron (BA.1)^ and S_761–775_^Omicron (BA.1)^. We also tested the ability of the same donors to expand T cells against the Omicron (BA.1) variant peptides S_486–505_^Omicron (BA.1)^ and S_761–775_^Omicron (BA.1)^. In these T cell lines, we observed no reactivity to either the Omicron (BA.1) or the Wuhan HU-1 peptides in any donors. These results indicate that Wuhan HU-1-reactive T cells against the two epitopes do not cross-recognize their mutated counterparts.

To attempt to gain insight into the mechanism surrounding complete loss of T cell activation, we assessed the ability of HLA-DR1 to bind S_486–505_ and S_761–775_ in Omicron (BA.1) form. Both S_486–505_^Omicron (BA.1)^ and S_761–775_^Omicron (BA.1)^ variant sequences demonstrated an improved ability to bind HLA-DR1: S_486–505_^Wuhan HU-1^ IC_50_ = 1,950.2 nM vs. S_486–505_^Omicron (BA.1)^ IC_50_ = 753.6 nM and S_761–775_^Wuhan HU-1^ IC_50_ > 10 μM vs. S_761–775_^Omicron (BA.1)^ IC_50_ = 6,777.2 nM (Figure 4E). Thus, Omicron (BA.1) mutations did not escape the CD4^+^ T cell response through inability to present peptide to T cells on HLA-DR1; in fact, the converse was found, with improved binding.

### Viral variant mutations directly alter TCR-facing residues

Given that S_486–505_^Omicron (BA.1)^ and S_761–775_^Omicron (BA.1)^ exhibited increased HLA-DR1 binding but lost T cell recognition, we sought to generate structures of these peptides bound to HLA-DR1 to understand this mechanistically.

**Figure 5:**
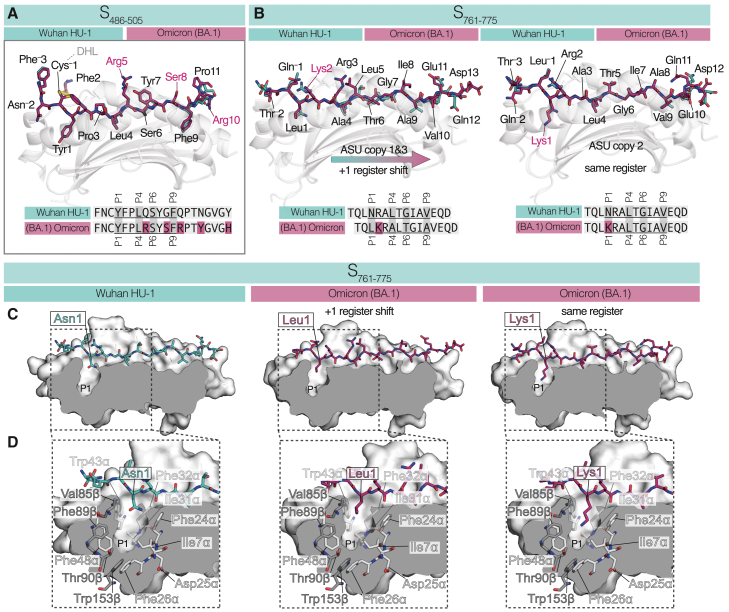
Structural implications of Omicron (BA.1) mutations on HLA-DR1 epitope presentation (A) Structural overview of HLA-DR1-S_486–505_^Omicron (BA.1)^ aligned and overlaid on top of the HLA-DR1-S_486–505_^Wuhan HU-1^ structure. For both, the HLA-DR1 peptide binding groove is shown as a gray cartoon representation, with the S_486–505_^Omicron (BA.1)^ peptide shown as sticks (C atoms, pink) and S_486–505_^Wuhan HU-1^ peptide shown as sticks (C atoms, aqua). Amino acid mutations contained within S_486–505_^Omicron (BA.1)^ that differ between variants are highlighted by the pink residue labels (inset). (B) Structural overview of HLA-DR1-S_761–775_^Omicron (BA.1)^ aligned and overlaid on top of the HLA-DR1-S_761–775_^Wuhan HU-1^ structure in two registers: (left) a +1 register shift relative to HLA-DR1-S_761–775_^Wuhan HU-1^ observed in asymmetric unit (ASU) copies 1and3 and (right) the same register as HLA-DR1-S_761–775_^Wuhan HU-1^ observed in ASU copy 2. Colored and represented as described in (A). (C) Surface cross-sectional view of the HLA-DR1 binding groove of HLA-DR1-S_761–775_^Wuhan HU-1^ (left), HLA-DR1-S_761–775_^Omicron (BA.1)^ +1 register shift (middle), and HLA-DR1-S_761–775_^Omicron (BA.1)^ same register (right). In each, the HLA-DR1 binding groove has been clipped in the z plane at approximately the deepest point in the P1 pocket. The residue buried in the deep hydrophobic P1 pocket is labeled with peptide represented as sticks (colored as previously). (D) Expanded cross-sectional view of the P1 pocket of S_761–775_ peptides/conformations. In each, residues that line the P1 pocket are shown as stick representations (DRA, light gray C atoms; DR1β, dark gray C atoms). Residues that form the back of the pocket (as viewed) are shown semi-transparent. Asn82β, which forms the front of the pocket (as viewed), is omitted for clarity.

S_486–505_^Omicron (BA.1)^ possesses mutations located within both the epitope core and the PFR as determined by the HLA-DR1-S_486–505_^Wuhan HU-1^ structure. The structure of HLA-DR1-S_486–505_^Omicron (BA.1)^ was solved at 1.6 Å and compared with the similar-resolution Wuhan HU-1 structure (Figure 5A). HLA-DR1 bound S_486–505_^Omicron (BA.1)^ using the same register. All core-positioned mutations were in potential upward-facing TCR contact positions, i.e., non-anchor residues: Q493R (P5) and G496S (P8). The overall peptide conformation remained highly similar to that of S_486–505_^Wuhan HU-1^, with the most drastic alteration induced by the Q493R mutation, which placed the differing elongated positive charge of P5-Arg in the center of the peptide core. The more subtle G496S resulting in P8-Ser did not alter backbone dihedrals, but placed an additional polar hydroxyl at this position. Thus, loss of cross-recognition between Wuhan HU-1 and the Omicron (BA.1) peptide variant of S_486–505_ was concurrent with mutations to epitope positions typically recognized by TCRs.

### A single viral variant mutation can induce a complete register shift in HLA-DR1 presentation

S_761–775_^Omicron (BA.1)^ contains a single mutation positioned at the archetypal P1 anchor position for HLA-DR1. This was particularly puzzling for the variant S_761–775_^Omicron (BA.1)^ peptide, which possessed only a single Leu → Lys change at position P1 defined by the structure of HLA-DR1-S_761–775_^Wuhan HU-1^ shown in Figure 3. This mutation, N764K, in fact increased HLA-DR1 binding affinity (Figure 4E), ruling out loss of HLA binding as an explanation for the loss of T cell recognition. The structure of HLA-DR1-S_761–775_^Omicron (BA.1)^ was solved at 2.5 Å resolution in space group C 2 2 2_1_ with three copies in the asymmetric unit allowing comparison to HLA-DR1-S_761–775_^Wuhan HU-1^ at similar resolutions (Figure 5B). Surprisingly, HLA-DR1-S_761–775_^Omicron (BA.1)^ exhibited two distinct peptide conformations within the three copies in the asymmetric unit whereby two copies (copies 1 and 3) exhibited a distinct peptide binding register compared with copy 2. These differential registers were supported by omit map analysis (Figure S5), which evidenced that HLA-DR1 can bind S_761–775_^Omicron (BA.1)^ by accommodating the N764K mutation into the P1 pocket and thus maintain the same binding register as S_761–775_^Wuhan HU-1^ (copy 2) but also incorporate the neighboring Leu into P1 (copies 1 and 3) to create a +1 register frameshift, i.e., TQLKRALTGIAVEQD to TQLKRALTGIAVEQD. This new register was accommodated by incorporating P1-Leu, P4-Ala, and P9-Ala, while placing the unfavored Thr at P6. The HLA-DR1 P1 binding pocket was able to accommodate the P1-Asn (S_761–775_^Wuhan HU-1^), P1-Leu, or P1-Lys (S_761–775_^Omicron (BA.1)^) within its deep hydrophobic pocket volume (Figure 5C). In each case, the residues surrounding the P1 pocket were positionally identical irrespective of buried cargo (Figure 5D), highlighting an extensive permissibility of the HLA-DR1 P1 pocket to accommodate diverse amino acids, including residues absent from the EL and BA motifs. While the +1 frameshifted register of the peptide was present in 2/3 of the copies within the asymmetric unit of the crystal structure, the relative distribution between the two registers in solution is unclear. Despite the results from these structural variants, the complete abrogation of T cell reactivity argues that, under physiological conditions, the shifted +1 register binding is favored. As a result, the frameshifted peptide epitope exhibited a drastically altered peptide landscape to the TCR repertoire compared with HLA-DR1-S_761–775_^Wuhan HU-1^, P3, Ala → Arg; P5, Thr → Leu; and P7, Ile → Gly, thus explaining the lack of immune cross-recognition against the Omicron (BA.1) variant of S_761–775_ peptide in Wuhan HU-1 Spike-experienced HLA-DR1-positive donors.

## Discussion

Our analyses of SARS-CoV-2 CD4^+^ T cell epitopes has provided insights into viral antigen recognition from immunodominance down to the molecular level via structural characterization of ancestral- and variant-derived viral epitopes presented by HLA-DR1. These first provide a resource of model-characterized HLA-II epitopes for tracking CD4^+^ T cells in COVID-19. Further, these data provide a base on which we could understand mechanistically the impact SARS-CoV-2 variants had on immune recognition of CD4^+^ T cells. The key outcome of this was that viral variants of two HLA-II-presented epitopes escaped pre-existing immune memory in two ways. An epitope from a highly variable region of Spike induced loss of CD4^+^ T cell memory via multiple mutations that are likely directly recognized by TCRs. A second more stable epitope contained a single amino acid mutation found in all Omicron lineages to date. Located at a key anchor position, which dictates peptide selection by HLA-DR1, this mutation did not lessen or ablate epitope presentation but instead induced a drastic alteration in its presentation by causing a register shift within the HLA-DR groove. The occurrence of register shift-inducing mutations may have wider implications in HLA-II-presented epitopes in the context of immune memory in general.

The most immunogenic HLA-II peptides are thought to have a predicted binding affinity of K_D_ <1,000 nM.^60^ All three Spike epitopes studied structurally exhibit *predicted* affinities for HLA-DR1 within this proposed threshold, yet we demonstrated that many showed experimentally weaker IC_50_ binding. These weaker-binding peptides could bind into the HLA-DR groove and elicit memory responses in individuals who were antigen experienced, which can be detected *in vitro* (this study) and *ex vivo*.^36^ Weaker-binding peptides may be compensated for by antigen abundance to reach sufficient antigen density.^61^ Indeed, SARS-CoV-2 Nucleocapsid, Spike, and Membrane proteins are the most abundant in infected cells at the protein^62^ and transcript^63^ levels. Together, these results highlight the difficulty in accurately predicting important and immunodominant HLA-II epitopes through *in silico* assessment alone of peptide-HLA-II binding.

Of the three Spike epitopes studied structurally, S_486–505_ was most frequently targeted by mutation. S_486–505_ is located within the receptor binding motif (RBM),^64^^,^^65^^,^^66^ which is highly mutable through positive selection at the Spike-ACE2 interface^67^ and the immunodominance of RBM-binding neutralizing antibodies.^68^ Mutations that enhance Spike-ACE2 binding are associated with increased replication efficiency^69^ and transmissibility.^70^ Indeed, Q493R, G496S, Q498R, and N501Y of Omicron (BA.1), present in S_486–505_, form new high-enthalpy receptor-ligand interactions.^71^ In contrast, Y505H is detrimental to Spike-ACE2 contacts^72^ and free energy estimations.^71^ Omicron strain Spike-ACE2 affinity is not significantly increased from the earlier-lineage Delta, which is thus suggestive that Omicron-lineage mutations may be driven by another factor,^72^ such as immune escape. Indeed, Omicron broadly escapes Spike-specific neutralizing antibodies,^51^^,^^52^^,^^53^^,^^54^ including N501Y.^73^ Viral accumulation of Q493R, which is an escape mutant of drug/antibody binding,^74^ and subsequent drug resistance were demonstrated in an individual treated with mAb cocktails of bamlanivimab and etesevimab.^75^ It is difficult to deduce whether these Omicron Spike mutations were driven by T cell escape, antibody escape, or viral-intrinsic evolution of fitness. Nevertheless, we have shown that mutations in S_486–505_ enable escape of epitope-specific CD4^+^ T cell immunity through alterations in TCR-facing residues that are sufficient to ablate T cell recognition in HLA-DR1^+^ donors.

S_761–775_ differs by being located within the S2 domain of Spike, which is more conserved across coronaviruses.^76^ S_761–775_ does not directly bind ACE2 and is not likely to be accessible to B cells,^77^^,^^78^ as it is within a tightly packed helix sandwiched between the central helical domain and the N-terminal domain of the pre-fusion Spike trimer.^79^ S_761–775_ exhibited a single mutation, N764K, which is persistent in all Omicron strains, having reached and maintained >98% genotypic frequency since around April 2022.^55^^,^^58^ The N764K mutation affected the archetypal P1 pocket of the S_761–775_ epitope presented by HLA-DR1 through its positioning, inducing a frameshift concurrent with loss of cross-recognition. Such frameshift mutations have been postulated previously in a DRB1^∗^07:01 epitope,^19^ but we herein demonstrate structural evidence of this mechanism of CD4^+^ T cell escape.

While we demonstrate two examples of CD4^+^ T cell epitope escape, T cell immunity toward SARS-CoV-2 viral variants is more robust compared with antibody immunity,^80^ potentially due to the sheer breadth of epitopes recognized by T cells.^81^ Characterization of the TCR clonotypes and the modes of their binding would aid the mechanistic explanations as to why, in the case of S_486–505_ and S_761–775_, TCRs of HLA-DR-restricted CD4^+^ T cells do not exhibit the capacity to cross-recognize their mutated variant counterparts. Nevertheless, these data highlight the importance of vaccinating against circulating Spike variants, i.e., Spike Omicron (BA.1) mRNA-1273.214^82^ and Omicron (BA.4/BA.5) BNT162b2,^83^ and/or targeting more conserved regions of coronaviral proteomes, which exhibit higher homology and also induce strong humoral and T cell responses.^84^

Our structural studies show how mutations to virus sequences may induce drastic alterations in HLA-II-presented peptides through either multiple epitope changes or seemingly subtle mutations. The net result of both observed mechanisms was the same (i.e., a loss of cross-recognition/memory), yet while the former may have been easily predicted due to the large number of mutations, the latter would not have been obvious without experimental characterization. The implication of this is that we require further ways to identify and predict the occurrence of register-shifting mutations and the frequency at which they influence CD4^+^ T cell recognition/memory such that future vaccines are appropriately designed to induce cross-protection against heterologous viruses.

### Limitations of the study

Our study set out during the pandemic to use the HLA-DR1 allotype as a model to assess the presentation and recognition of SARS-CoV-2 by CD4^+^ T cells. Emerging epitopes were selected (circa November 2020) from published studies that performed thorough and extensive epitope mapping throughout the SARS-CoV-2 genome. By selecting peptides from these published data, we have not characterized the full breadth of potential HLA-DR1 epitopes. As the peptides were selected from a combination of studies describing immunogenicity in unexposed individuals^14^ and individuals who were SARS-CoV-2 experienced,^15^^,^^16^ we have not drawn conclusions from the collective responses to individual peptides observed in our donors to inform whether donors had (1) experienced a previous infection, (2) responded to vaccination, or (3) immune protection against viral infection/reinfection. This is in part because we did not screen peptide responses against the full breadth of epitopes, but also because some peptides were immunogenic in individuals who were unexposed (pre-/early pandemic), and thus may represent cross-reactive memory to antigens other than SARS-CoV-2. Similarly, the effect of variant mutations on the breadth of HLA-DR1 epitopes was not studied, as we could focus our in-depth structural analysis on only a small subset of peptides. Thus, the overall rate of epitope loss within host-viral evolution and whether other mechanisms of escape also exist cannot be determined. Nevertheless, our study highlights the consequences that viral evolution could have generally on the peptide epitopes presented to CD4^+^ T cells on the HLA-II platform and that could have relevance in all host-viral interactions.

## STAR★Methods

### Key resources table

**Table:** 

REAGENT or RESOURCE	SOURCE	IDENTIFIER
**Antibodies**		

anti-CD3-APC (clone UCHT1)	Biolegend	Cat#300412; RRID: AB_314066
anti-CD4-BV421 (clone RPA-T4)	Biolegend	Cat#300532; RRID: AB_10965645
anti-CD8-BV786 (RPA-T8)	BD Bioscience	Cat#563823; RRID: AB_2687487
anti-IFN-γ-FITC (clone B27)	Biolegend	Cat#506504; RRID: AB_315437
anti-TNF-α-PE-Cy7 (clone MAb11)	Biolegend	Cat#502930; RRID: AB_2204079
anti-HLA-DR (clone L243)	Biolegend	Cat#307667; RRID: AB_2800798

**Bacterial and virus strains**		

Escherichia coliBL21(DE3) Chemically Competent cells	ThermoFisher Scientific	Cat#C600003

**Biological samples**		

Peripheral blood of local HLA-DR1^+^ and DR4^+^ individuals	This manuscript	N/A

**Chemicals, peptides, and recombinant proteins**		

Synthetic peptide S_1-15_ MFVFLVLLPLVSSQC	Peptide Protein Research	Custom synthesis
Synthetic peptide S_167-181_ TFEYVSQPFLMDLEG	Peptide Protein Research	Custom synthesis
Synthetic peptide S_235-249_ ITRFQTLLALHRSYL	Peptide Protein Research	Custom synthesis
Synthetic peptide S_241-255_ LLALHRSYLTPGDSS	Peptide Protein Research	Custom synthesis
Synthetic peptide S_339-353_ GEVFNATRFASVYAW	Peptide Protein Research	Custom synthesis
Synthetic peptide S_344-358_ ATRFASVYAWNRKRI	Peptide Protein Research	Custom synthesis
Synthetic peptide S_486-505_ FNCYFPLQSYGFQPTN GVGY	Peptide Protein Research	Custom synthesis
Synthetic peptide S_511-530_ VVLSFELLHAPATVCG PKKS	Peptide Protein Research	Custom synthesis
Synthetic peptide S_512-526_ VLSFELLHAPATVCG	Peptide Protein Research	Custom synthesis
Synthetic peptide S_749-763_ CSNLLLQYGSFCTQL	Peptide Protein Research	Custom synthesis
Synthetic peptide _S761-775_ TQLNRALTGIAVEQD	Peptide Protein Research	Custom synthesis
Synthetic peptide S_818-832_ IEDLLFNKVTLADAG	Peptide Protein Research	Custom synthesis
Synthetic peptide S_866-880_ TDEMIAQYTSALLAG	Peptide Protein Research	Custom synthesis
Synthetic peptide S_990-1004_ EVQIDRLITGRLQSL	Peptide Protein Research	Custom synthesis
Synthetic peptide S_1015-1029_ AAEIRASANLAATKM	Peptide Protein Research	Custom synthesis
Synthetic peptide E_56-70_ FYVYSRVKNLNSSRV	Peptide Protein Research	Custom synthesis
Synthetic peptide M_176-190_ LSYYKLGASQRVAGD	Peptide Protein Research	Custom synthesis
Synthetic peptide N_49-63_ TASWFTALTQHGKED	Peptide Protein Research	Custom synthesis
Synthetic peptide N_108-122_ WYFYYLGTGPEAGLP	Peptide Protein Research	Custom synthesis
Synthetic peptide N_127-141_ KDGIIWVATEGALNT	Peptide Protein Research	Custom synthesis
Synthetic peptide N_224-238_ LDRLNQLESKMSGKG	Peptide Protein Research	Custom synthesis
Synthetic peptide N_328-342_ GTWLTYTGAIKLDDK	Peptide Protein Research	Custom synthesis
Synthetic peptide NSP3_1350-1364_ KSAFYILPSIISNEK	Peptide Protein Research	Custom synthesis
Synthetic peptide NSP6_3801-3815_ NRYFRLTLGVYDYLV	Peptide Protein Research	Custom synthesis
Synthetic peptide NSP12_5019-5033_ PNMLRIMASLVLARK	Peptide Protein Research	Custom synthesis
Synthetic peptide NSP14_6420-6434_ LDAYNMMISAGFSLW	Peptide Protein Research	Custom synthesis
Synthetic peptide ORF6_13-27_ EILLIIMRTFKVSIW	Peptide Protein Research	Custom synthesis
Synthetic peptide ORF8_3-17_ FLVFLGIITTVAAFH	Peptide Protein Research	Custom synthesis
Synthetic peptide ORF8_43-57_ SKWYIRVGARKSAPL	Peptide Protein Research	Custom synthesis
Synthetic peptide S_486-505_ (Omicron BA.1) FNCYFPLRSY SFRPTYGVGH	Peptide Protein Research	Custom synthesis
Synthetic peptide S761-775 (Omicron BA.1) TQLKRALTGIAVEQD	Peptide Protein Research	Custom synthesis
Synthetic peptide CLIP105–117 SKMRMATPLLMQA	Peptide Protein Research	Custom synthesis
Synthetic peptide N-terminally biotinylated CLIP99–117 bt-LPKPPKPVSKMRMAT PLLMQA	Peptide Protein Research	Custom synthesis
Lymphoprep	Axis-Shield	Cat#1114547
Pierce™ Protein A IgG Plus Orientation Kit, 2 mL	Thermo Scientific	Cat#44893
Human recombinant IL-2 (Proleukin®)	Cardiff and Vale University Health Board Pharmacy	N/A
CTL Test Plus media	CTL Europe	Cat#CTLTP-005
RPMI 1640	ThermoFisher Scientific	Cat#11875093

**Critical commercial assays**		

Streptavidin-HRP	R&D Systems (bio-techne)	Cat# DY998
BD OptEIA™ TMB Substrate Reagent Set	BD Biosciences	Cat# 555214; RRID: AB_2869044
ELISpot Flex: Human IFN-γ (ALP)	Mabtech	Cat# 3420-2A
ELISPOT plates	Sigma-Aldrich	Cat# MAIPS4510
TOPS Crystallization Screen	Molecular Dimensions	Custom product. Bulek et al.^85^
PACT premier™ HT-96 Crystallization Screen	Molecular Dimensions	Cat# MD1-36
MojoSort™ Human CD4 T Cell Isolation Kit	Biolegend	Cat# 480010
FIX & PERM Cell Fixation & Cell Permeabilization	BD Biosciences	Cat# 554714
LIVE/DEAD™ Fixable Aqua	ThermoFisher Scientific	Cat# L34957

**Deposited data**		

HLA-DR1-S_486-505_	This manuscript	PDB: 8CMB
HLA-DR1-S_511-530_	This manuscript	PDB: 8CMC
HLA-DR1-S_761-775_	This manuscript	PDB: 8CMD
HLA-DR1-M_176-190_	This manuscript	PDB: 8CME
HLA-DR1-nsp3_1350-1364_	This manuscript	PDB: 8CMF
HLA-DR1-nsp14_6420-6434_	This manuscript	PDB: 8CMG
HLA-DR1-S_486-505_^Omicron (BA.1)^	This manuscript	PDB: 8CMH
HLA-DR1-S_761-775_^Omicron (BA.1)^	This manuscript	PDB: 8CMI
3,013-genome subsample of the ncov/gisaid/global/all-time dataset	GISAID contributors	Table S3
3,214 genome subset of the ncov/gisaid/oceania/all-time dataset	GISAID contributors	Table S3

**Experimental models: Cell lines**		

T2 (174 x CEM.T2)	ATCC	CRL-1992™; RRID: CVCL_2211
T2-DR1 (DRA^∗^0101, DRB1^∗^0101 lentrivirally transduced)	Cole, Godkin labs	Theaker et al.^86^

**Recombinant DNA**		

pGEM-T7	Promega	Cat#A3600
pGEM-T7-DRA1^∗^0101	Cole, Godkin labs	Greenshields-Watson et al.^22^
pGEM-T7-DRB1^∗^0101	Cole, Godkin labs	Greenshields-Watson et al.^22^

**Software and algorithms**		

PyMOL (v2.5 open-source build)	Maintained by Schrodinger	RRID:SCR_000305
Phaser	Phenix Online	RRID:SCR_014219
Phenix v1.20	Phenix Online	RRID:SCR_014224
CCP4	Collaborative Computational Project No. 4	RRID:SCR_007255
Molprobity	Duke University^87^	RRID:SCR_014226
Xia2 pipeline	Diamond Light Source (DLS)	RRID:SCR_015746
Dials	DLS, Lawrence Berkeley National Laboratory and STFC	https://dials.github.io/about.html
XDS program package	Max Planck Institute for Medical Research	RRID:SCR_015652
COOT v0.9.6	MRC Laboratory of Molecular Biology	RRID:SCR_014222
AceDRG	Collaborative Computational Project No. 4	RRID:SCR_007255
Nextstrain interactive portal	Nextstrain (Hadfield et al.)^58^	https://nextstrain.org/ncov/gisaid/global/6m
FlowJo	FlowJo LLC	RRID:SCR_008520
NetMHCpanII v4.1	DTU Health Tech Department of Health Technology	https://services.healthtech.dtu.dk/services/NetMHCpan-4.1/
Matplotlib	The Matplotlib development team	RRID:SCR_008624
Seaborn	Michael Waskom	RRID:SCR_018132
SciPy	The SciPy Project	RRID:SCR_008058

### Resource availability

#### Lead contact

Further information and requests for resources and reagents should be directed to and will be fulfilled by the lead contact, Andrew Godkin (godkinaj@cardiff.ac.uk).

#### Materials availability

Cell lines and protein expression plasmids are available from the lead contact on request. All other reagents are available to purchase from commercial suppliers.

### Experimental model and study participant details

#### Ethical approval, participant recruitment & consenting

Ethical approval was obtained from the Medical School Research Ethics Committee, Cardiff University to study “Immune surveillance and recognition of infection and cancer” in local healthy individuals. Study participants were recruited via an open advertisement for volunteers within the Heath Park Campus, Cardiff, UK. Informed consent was obtained from all volunteering donors. Donors were aged 31-59 years old (mean age 39 years old) at time of their first donated sample. The cohort consisted of four females and four males. Ethnicity was not recorded during consenting. Participation in the UK COVID-19 vaccination campaign was not a requirement for study participation. HLA-typing was performed by the Welsh Transplantation and Immunogenetics Laboratory (Pontyclun, UK). Blood donations to test the Wuhan HU-1 peptide panel were taken between November 2020 and November 2021. Blood donations to compare Wuhan HU-1 and Omicron (BA.1) peptides were taken between March 2022 and October 2022.

#### Generation of cultured T cell lines from donor PBMCs

PBMCs were isolated over ficoll gradient (Lymphoprep, Axis-Shield). Subsequent isolated PBMCs were cultured at 2×10^6^ cells/mL in CTL Test Plus (CTL Europe), 2 mM L-glutamine, 100 U/mL penicillin & 100 μg/mL streptomycin (Sigma-Aldrich) media in 96-well plates incubated at 37 °C, 5 % CO_2_. Cultured T cell lines were generated by addition of 5 μg/mL of candidate test peptide at day 0. Cultured cells were replenished with fresh media containing 20 IU/mL IL-2 on days 3,7 & 10 before harvest at day 12-14.

For long term CD4^+^ T cell lines with identified specificity, cells were first sorted using a MojoSort™ Human CD4 T Cell Isolation Kit (Biolegend, UK) according to manufacturer’s instructions. Enriched CD4^+^ T cells were then expanded using co-culture with irradiated T2 cells (detailed below). T2-DR1 cells were loaded with 2 μg/mL of relevant peptides for 2 hrs before washing and irradiating. CD4^+^ T cells were co-cultured with irradiated peptide loaded T2-DR1 (as APCs) and irradiated autologous PBMCs (as feeder cells) and analysed for functionality 14 days after restimulation.

#### Cell lines

T2 cells (T2 (174 x CEM.T2) - CRL-1992) transduced with HLA-DR1 (HLA-DRA^∗^0101, DRB1^∗^0101) were generated previously and denoted as T2-DR1 cells.^22^^,^^86^ T2-DR1 cells were maintained in suspension at 37 °C, 5 % CO_2_ in RPMI-1640 media (GIBCO) supplemented with 10% heat inactivated fetal calf serum (gibco), 2 mM l-glutamine, 100 U/mL penicillin, 100 μg/mL streptomycin (all gibco/ThermoFisher Scientific). Cells were passaged every two to three days, maintaining cell density between 1x10^6^ and 1x10^7^ cells/mL.

### Method details

#### Production of peptide-HLA-DR1 proteins

HLA-DR1 (HLA-DRA^∗^0101 & HLA-DRB1^∗^0101) proteins were expressed and refolded *in vitro* from inclusion bodies as previously described.^50^^,^^88^ HLA-DRA and –DRB chains were produced separately in BL21(DE3) *Escherichia coli* cells via autoinduction expression.^89^ Inclusion bodies were extracted and solubilized in 50 mM Tris, 8 M Urea, 2 mM EDTA, 100 mM NaCl, 1 mM DTT. Peptide-HLA-DR1 molecules were refolded by rapid dilution of HLA-DRA & -DRB inclusion bodies into refold buffer (20 mM TRIS pH 8.5, 30 % w/v glycerol, 1 mM EDTA, 20 mM NaCl, 0.3 mM cystamine & 0.6 mM cysteamine) in the presence of synthetically synthesized peptides (Peptide Protein Research Ltd.) at final concentrations of 10 μg/mL for each –DR chain and 0.5 μg/mL of peptide. Refolds were incubated at 4 °C for 2-3 days before sequential diafiltration with 10 mM Tris pH 8.1, 150 mM NaCl and 10 mM Tris pH 8.1 using 30 kDa MWCO filtration devices (Sartorius). Buffer exchanged peptide-HLA-DR1 samples were purified via 1) anion exchange (HiTrap® Q High Performance) attached to an AKTA Pure FPLC system (Cytiva), 2) affinity chromatography purified using the conformationally specific anti-HLA-DR antibody clone L243 chemically crosslinked to Protein A gravity columns (Thermo Scientific) and 3) size-exclusion chromatography using a Superdex®200 Increase 10/300 GL column (Cytiva). Final samples were purified into a buffer solution of 10 mM Tris pH 8.1, 150 mM NaCl. Sample purity was analyzed by SDS-PAGE.

#### HLA-II peptide binding assays

HLA-DR1 proteins were expressed as above, refolded with CLIP_105–117_ (SKMRMATPLLMQA), henceforth DR1-CLIP_105–117_. Competitive inhibition HLA-DR1 binding assays were performed as previously described.^37^^,^^50^ Peptide exchange reactions were prepared in 20 mM MES pH 5, 140 mm NaCl, 0.02% NaN_3_. Each reaction contained 0.1 μg of refolded DR1-CLIP_105–117_, 45 nM N-terminally biotinylated CLIP_99–117_ (bt-LPKPPKPVSKMRMATPLLMQA) marker peptide and the candidate test peptide. Candidate test peptides were 10-fold serially diluted (10^−4^-10^−10^ M) and added to the exchange reaction before incubation overnight at 37 °C. After incubation, exchange reactions were neutralized with 1 M Tris, 10 % BSA, 1 % Tween, 0.02 % NaN_3_, pH 8 and transferred to high-bind ELISA plates coated with the anti-HLA-DR antibody clone L243. After capture, biotinylated CLIP_99–117_ was detected using streptavidin-HRP (R&D Systems) and colorimetric HRP substrate reagent (BD Bioscience).

#### Peptide immunogenicity analysis

Short-term T cell lines cultured after day 12 were directly restimulated with candidate peptide and assayed via IFN-γ ELISpot assay. T cell lines were washed, counted and plated in duplicate onto anti-IFN-γ coated PVDF filter membrane plates (Mabtech) with addition of 5 μg/mL candidate peptide. For each T cell line, response to restimulation with peptide was compared to a no peptide (media) control. Plates were incubated overnight (∼16 hrs.) at 37 °C, 5% CO_2_ before developing as described by manufacturer protocol. Developed plates were imaged and spot forming cells (sfcs) counted using an ImmunoSpot S6 Ultra (CTL Europe).

For long-term T cell lines, post-12 days were restimulated with candidate peptide-pulsed T2-DR1 cells (generated as described above). T2-DR1 cells were first pulsed with 5 μg/mL of candidate peptide for 2 hrs., washed to remove unbound peptide, then co-cultured with T cells in the presence of 10 μg/mL Brefeldin A. Co-cultured activation assays were incubated overnight (∼16 hrs.) at 37 °C, 5% CO_2_. Unpulsed T2-DR1 cells (no peptide/media) were used as negative control. Following co-culture, cells were stained with LIVE/DEAD™ Fixable Aqua (ThermoFisher Scientific), anti-CD3-APC, anti-CD4-BV421 antibodies (all Biolegend) and anti-CD8-BV780 (BD Biosciences), subsequently treated with FIX & PERM Cell Fixation & Cell Permeabilization (BD Biosciences) and then stained with anti-IFN-γ-FITC & TNF-α-PE-Cy7 (both Biolegend) for 20 mins at 4 °C in the absence of light. Stained cells were analyzed on a NovoCyte 3000 (Agilent Technologies Inc.) and analyzed using FlowJo v10 (FlowJo LLC). Heatmap and swarm/box plots presented using *seaborn.*^90^

#### Crystallization and structure determination of peptide-HLA-DR1 complexes

Purified peptide-HLA-DR1 samples were screened for crystallization using the vapor diffusion method using screen plates dispensed by a Crystal Gryphon (Art Robbins Instruments, LLC) at drop volumes of 0.4 μL in sitting drop format. Further condition screening was performed using hanging drop experiments hand-pipetted in EasyXtal 15-Well plates (Nextal) with a drop volume of 3 μL. Peptide-HLA-DR1 complexes crystallized at 18 °C within 2 – 30 days in a variety of conditions outlined in Table S2.

Obtained crystals of sufficient size for diffraction were flash frozen in crystallization solution supplemented with either 10 % glycerol or 10 % ethylene-glycol. Crystals were exposed to x-rays at Diamond Light Source beamlines i04 (Harwell Science and Innovation Campus, Oxfordshire, United Kingdom). Diffraction data were reduced using xia2^91^ which implements DIALS^92^ and XDS.^93^ Phases were estimated using molecular replacement implemented by PHASER^94^ using various HLA-DR1 models as search models depending on space group. For each, peptide atoms were removed from search models to prevent phases biasing peptide atom placement. Details specific to each structure are detailed in Table S2. Refinement of structures was performed using iterative rounds of refinement using *phenix.refine* of the PHENIX suite^95^ and manual model editing using COOT v0.9.6. TLS groups for TLS refinement were determined using *phenix.find_tls_groups.* Progress of model quality during refinement was assessed by MolProbity.^87^^,^^96^ For HLA-DR1-S_486-505_ and HLA-DR1-S_486-505_^Omicron (BA.1)^, coordinates for cysteamine (2-Aminoethanethiol, PDB ligand code: DHL) were obtained from the COOT ligand dictionary after which covalent link restraints to the cysteine thiol sulfur atom were generated using AceDRG,^97^ part of the CCP4 suite.^98^ Subsequent refinements proceeded by defining this covalent constraint using the *refinement.geometry_restraints.edits* parameter during *phenix.refine*.

Omit maps were calculated by performing a two macro-cycle refinement (bulk solvent and scaling, local real-space refinement, simulated annealing (cartesian), reciprocal space refinement, occupancy refinement) in the absence of all peptide atoms. This was achieved by passing the following command line parameter definitions to the *phenix.refine* default refinement strategy: *simulated_annealing=True, main.number_of_macro_cycles=2, tls=False*. Molecular visualizations of structures were generated using the open-source build of Pymol V.2.5.0 (Schrodinger, LLC). CCP4 format electron density maps were generated using *phenix.mtz2map*. Final model co-ordinates and structure factors were submitted to the Protein Data Bank under accession codes: DR1-S_486-505_ = 8CMB, DR1-S_511-530_ = 8CMC, DR1-S_761-775_ = 8CMD, DR1-M_176-190_ = 8CME, DR1-nsp3_1350-1364_ = 8CMF, DR1-nsp14_6420-6434_ = 8CMG, DR1-S_486-505_^Omicron (BA.1)^ = 8CMH, DR1-S_761-775_^Omicron (BA.1)^ = 8CMI.

#### SARS-CoV-2 mutational sequence analysis

Analysis of variant mutations was performed using the Nextstrain^58^ ncov interactive visualization portal which is enabled by data from GISAID^55^ and its contributors – a full acknowledgements list is shown in Table S3: References for accessed GISAID contributor data. A 3,013-genome subsample of the ncov/gisaid/global/all-time dataset was used to display the genotypic per-residue frequency of amino acid usage encompassing epitope regions over time (16^th^ December 2019 – 10^th^ June 2022; Accessed 14^th^ June 2022) within the subsampled dataset. For analysis of nsp14_6420-6434_ L6433F in Australasia, a 3,214 genome subset of the ncov/gisaid/oceania/all-time dataset (4^th^ Jan 2020 – 14^th^ June 2022; Accessed 15^th^ June 2022) was used. Defining mutations of Variants of Concern/Variants of Interest were accessed as defined by the CoVariants portal^59^ which is enabled by Nextstrain and data from GISAID.

### Quantification and statistical analysis

#### HLA-II peptide binding assays

All assays were performed in triplicate (n=3 technical replicate wells) in two independent experiments (n=2). Mean, standard deviation and IC50 were calculated from a representative example. Raw absorbance data were normalized to a no-competitor peptide control to derive percentage competitive inhibition by each candidate peptide. Mean percentage competitive inhibition values and standard deviation were calculated using *SciPy.*^99^ IC50 values were calculated by fitting a four-parameter log(inhibitor) response function by *math.curve_fit* of *SciPy*, using the following initial values for curve fitting: slope = SIGN(Y at XMAX – Y at XMIN), min = YMIN, max = YMAX & IC50 = X at YMID. Data were plotted and presented using *matplotlib.*^100^

#### ELISpot analyses

Assays were performed in duplicate peptide stimulations (n=2 technical replicate wells) at two independent two time points (n=2 peripheral blood donations per donor). For each blood donation, replicate wells were averaged (mean) and background subtracted by a no peptide control well (media) for each corresponding cultured line. Spot forming cells (sfcs) were normalized to sfcs/10,000 cells based on total number of plated cells in each assay well. The maximal response for each peptide from the two time points was taken as a maximal response. A positive response to a peptide by a donor was considered if maximal response was ≥ 25 sfcs/10,000 cells above background. Data were binned into no response (< 25 sfcs/10,000 cells), low (25-50) moderate (50-100) and high (100+) for the purpose of heat map visualization clarity.

To evaluate the peptide response rate in the donor cohort, the number of responses (tests) was divided by the total number of tests (i.e. number of donors multiplied by number of peptides tested) and represented as a percentage. This equated to 87 total tests for HLA-DR1^+^ donors and 135 total tests for HLA-DR4^+^ donors. A Fisher’s exact test was used to evaluate an association between DR1^+^/DR4^+^ status (groups) and the positive responses to peptides (outcomes). Significance was defined by P < 0.01.

## Data Availability

• Final model co-ordinates and structure factors were submitted to the Protein Data Bank under accession codes: DR1-S_486-505_ = PDB: 8CMB, DR1-S_511-530_ = PDB: 8CMC, DR1-S_761-775_ = PDB: 8CMD, DR1-M_176-190_ = PDB: 8CME, DR1-nsp3_1350-1364_ = PDB: 8CMF, DR1-nsp14_6420-6434_ = PDB: 8CMG, DR1-S_486-505_^Omicron (BA.1)^ = PDB: 8CMH, DR1-S_761-775_^Omicron (BA.1)^ = PDB: 8CMI. Raw data from all non-structural related figures are available from Mendeley Data: https://doi.org/10.17632/5hh7rwwttm.2. • Structural analysis code is available at: https://zenodo.org/record/8047547. • Any additional information required to reanalyze the data reported in this paper is available from the lead contact upon request.

## Supplemental information

Document S1. Figures S1–S5 and Tables S1 and S2

Table S3. References for accessed GISAID contributor data, related to Figures 4A, S4A–S4C (ncov/gisaid/global/all-time), and S4D (ncov/gisaid/oceania/all-time)

Document S2. Article plus supplemental information
