# Supplementary material for: Structural definition of HLA class II-presented SARS-CoV-2 epitopes reveals a mechanism to escape pre-existing CD4+ T cell immunity
Source: Cell Rep. 2023 Jul 19;42(8):112827. doi: 10.1016/j.celrep.2023.112827 (PMC10840515; doi:10.1016/j.celrep.2023.112827)
Supplement: Document S1. Figures S1–S5 and Tables S1 and S2 [file mmc1.pdf]

Cell Reports, Volume *42*

Supplemental information

Structural definition of HLA class II-presented SARS-CoV-2 epitopes reveals a mechanism to escape pre-existing CD4^{+} T cell immunity

Yuan Chen, Georgina H. Mason, D. Oliver Scourfield, Alexander Greenshields-Watson, Tracey A. Haigh, Andrew K. Sewell, Heather M. Long, Awen M. Gallimore, Pierre Rizkallah, Bruce J. MacLachlan, and Andrew Godkin

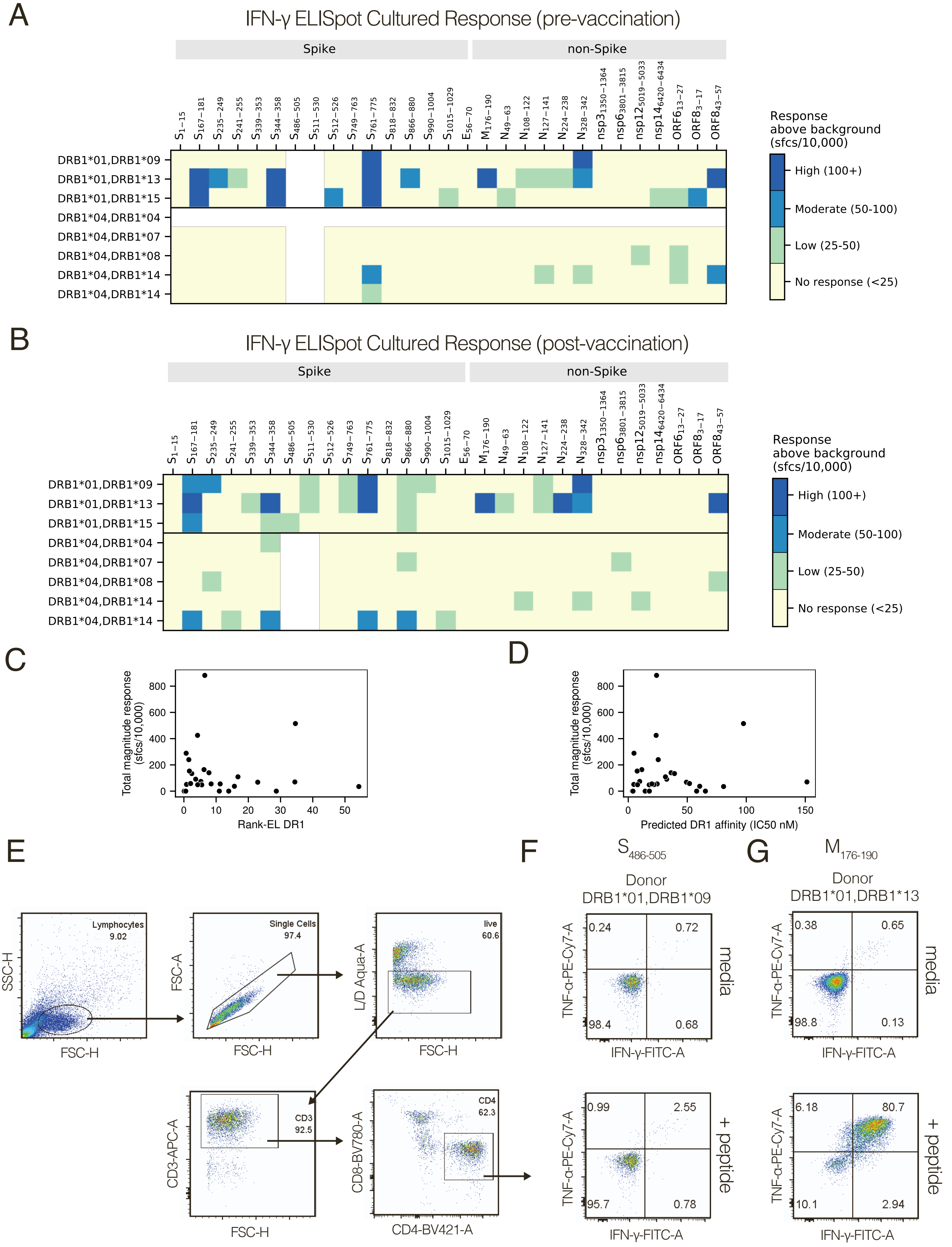
A
IFN-γ ELISpot Cultured Response (pre-vaccination)
Spike
non-Spike
Response above background (sfcs/10,000)
High (100+)
Moderate (50-100)
Low (25-50)
No response (<25)
DRB1*01,DRB1*09
DRB1*01,DRB1*13
DRB1*01,DRB1*15
DRB1*04,DRB1*04
DRB1*04,DRB1*07
DRB1*04,DRB1*08
DRB1*04,DRB1*14
DRB1*04,DRB1*14
B
IFN-γ ELISpot Cultured Response (post-vaccination)
C
Total magnitude response (sfcs/10,000)
Rank-EL DR1
D
Predicted DR1 affinity (IC50 nM)
E
Lymphocytes 9.02
Single Cells 97.4
live 60.6
CD3 92.5
CD4 62.3
SSC-H
FSC-H
FSC-A
L/D Aqua-A
CD3-APC-A
CD8-BV780-A
CD4-BV421-A
F
S486-505
Donor DRB1*01,DRB1*09
G
M176-190
Donor DRB1*01,DRB1*13
TNF-α-PE-Cy7-A
IFN-γ-FITC-A
media
+ peptide

Figure S1 – Immunogenicity of selected SARS-CoV-2 peptides in HLA-DR1$^+$ and HLA-DR4$^+$ donors. **A)** Heat map summary of IFN-γ ELISpot responses to overnight restimulation with SARS-CoV-2 peptides (15 – 20mer length) following 12-day culture of PBMCs in presence of candidate peptide before vaccination. Donors are grouped by HLA-DR1$^+$ and HLA-DR4$^+$ status. ELISPOT assays performed in duplicate. Responses were background subtracted (media only), normalized to sfcs/10,000 cells and binned into low, moderate and high responders (cut-offs and colors indicated inset). **B)** Heatmap summary of IFN-γ ELISpot responses as described in A) following SARS-CoV-2 vaccination. **C)** Scatter plot summary of total magnitude response to each peptide (summated maximal response by each donor for each peptide) in HLA-DR1$^+$ donors against predicted rank eluted likelihood (Rank-EL) as determined by NetMHCIIpan v4.1. No correlation was observed. **D)** Scatter plot summary of total magnitude response to each peptide in HLA-DR1$^+$ donors against predicted binding affinity (IC_{50} nM) as determined by NetMHCIIpan v4.1. No correlation was observed. **E)** Representative gating strategy analyzing lymphocyte gate, single cells, live cells, CD3$^+$, CD4$^+$ cells. Gating strategy applied in D&E. **F)** TNF-α & IFN-γ intracellular cytokine staining of $S_{761\text{-}775}$ T cell line in response to $S_{761\text{-}775}$ pulsed T2-DR1$^+$ cells. Response of T cell/APC co-culture in absence of peptide (media) control is shown. **G)** TNF-α & IFN-γ intracellular cytokine staining of $M_{176\text{-}190}$ T cell line in response to $M_{176\text{-}190}$ pulsed T2-DR1$^+$ cells. Response of T cell/APC co-culture in absence of peptide (media) control is shown.

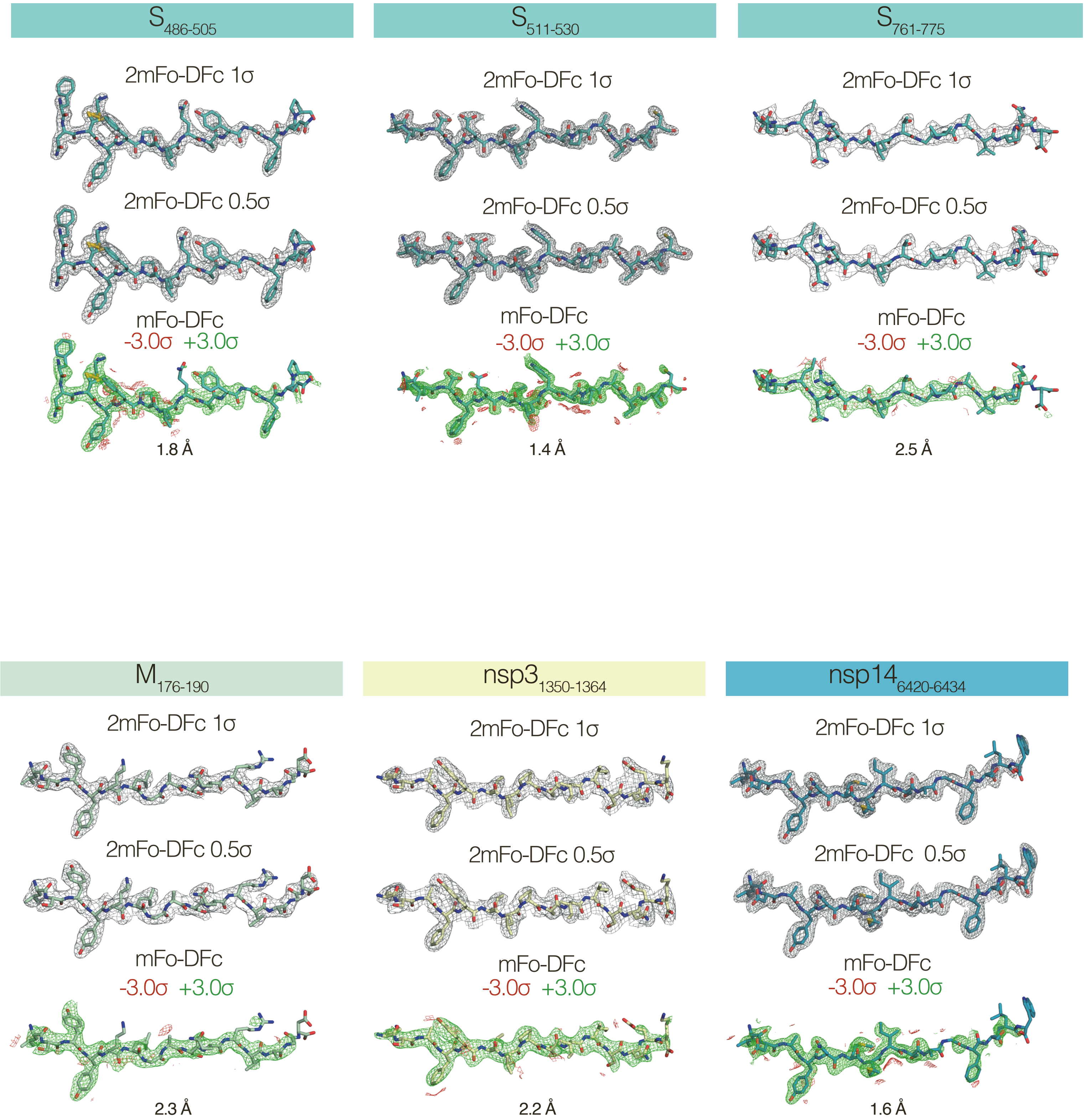

S486-505
2mFo-DFc 1σ
2mFo-DFc 0.5σ
mFo-DFc
-3.0σ +3.0σ
1.8 Å
S511-530
2mFo-DFc 1σ
2mFo-DFc 0.5σ
mFo-DFc
-3.0σ +3.0σ
1.4 Å
S761-775
2mFo-DFc 1σ
2mFo-DFc 0.5σ
mFo-DFc
-3.0σ +3.0σ
2.5 Å
M176-190
2mFo-DFc 1σ
2mFo-DFc 0.5σ
mFo-DFc
-3.0σ +3.0σ
2.3 Å
nsp3 1350-1364
2mFo-DFc 1σ
2mFo-DFc 0.5σ
mFo-DFc
-3.0σ +3.0σ
2.2 Å
nsp14 6420-6434
2mFo-DFc 1σ
2mFo-DFc 0.5σ
mFo-DFc
-3.0σ +3.0σ
1.6 Å

Figure S2 – Electron density maps and omit map analysis of peptide-HLA-DR1 structures. 2mFo-DFc contoured at 1.0 σ (top), 0.5 σ (middle) and omit map mFo-DFc difference map (bottom) are shown for each peptide-HLA-DR1 structure. In each, peptide is shown as stick representation, colored with C=indicated inset, N=blue, O=red. 2mFo-DFc maps are presented as grey mesh. For omit map analysis, a two macro-cycle refinement including simulated annealing was performed using *phenix.refine* in the absence of peptide atoms. A resultant mFo-DFc map was calculated from the omit map refinement. For each omit map, positive difference map peaks (green mesh, +3.0 σ) and negative difference map peaks (red mesh, -3.0 σ) are shown overlayed with the final peptide co-ordinates which were absent in their calculation.

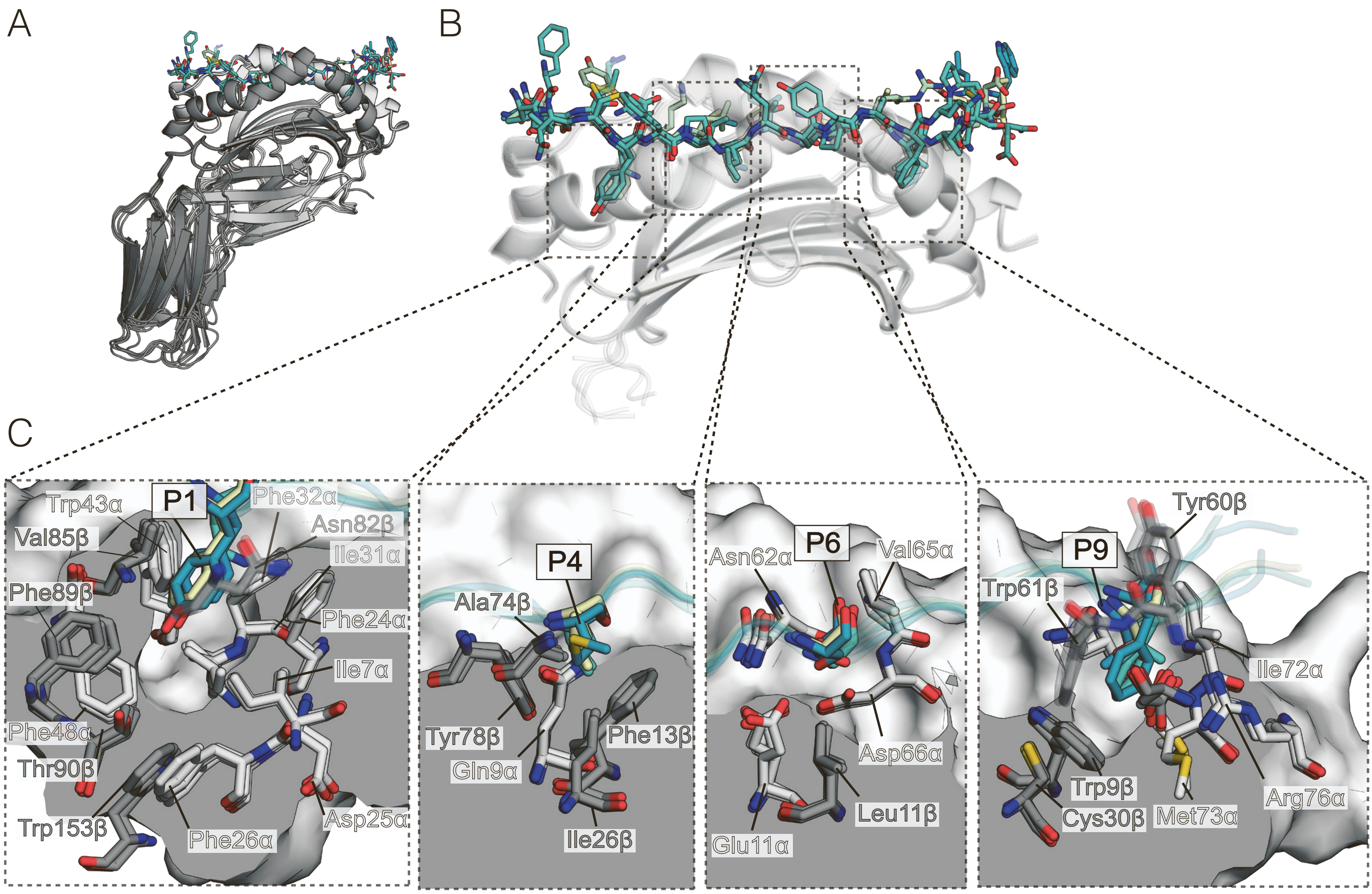

	P1 pocket	P4 pocket	P6 pocket	P9 pocket
$S_{486-505}$	Y	L	S	F
$S_{511-530}$	F	L	A	T
$S_{761-775}$	N	L	G	V
$M_{176-190}$	Y	G	S	V
$nsp3_{1350-1364}$	F	L	S	S
$nsp14_{6420-6434}$	Y	M	S	F

Figure S3 – Comparison of each SARS-CoV-2 peptide-HLA-DR1 structure. A) Structural alignment of the six solved peptide-HLA-DR1 complexes (HLA-DR1-$S_{486-505,}$ -$S_{511-530}$, -$S_{761-775}$, -$M_{176-190,,}$ -$nsp3_{1350-1364}$ and – $nsp14_{6420-6434}$). An overall view is shown of HLA-DR1 (grey, cartoon representation). Each peptide is shown as sticks and colored by atom (C= indicated inset matching to viral protein origin as in Fig 3, N=blue, O=red, S=yellow). **B)** A zoomed in view of the aligned peptide-HLA-DR1 binding groove for each structure as in A). **C)** Focused views of the P1, P4, P6 & P9 binding pockets of aligned peptide-HLA-DR1 structures. In each, the HLA-DR1 residues which form the binding pocket are shown as sticks (DRA: light grey, DRB: dark grey). Each peptide is shown (cartoon, semi-transparent) with the residue bound into each pocket show as opaque sticks. Residues which are in front of the bound peptide are shown semi-transparent for clarity (e.g. Asn82β). A surface cross-sectional view of the HLA-DR1 binding groove clipped in the z-plane at approximately the deepest point in the P1 pocket is also shown.

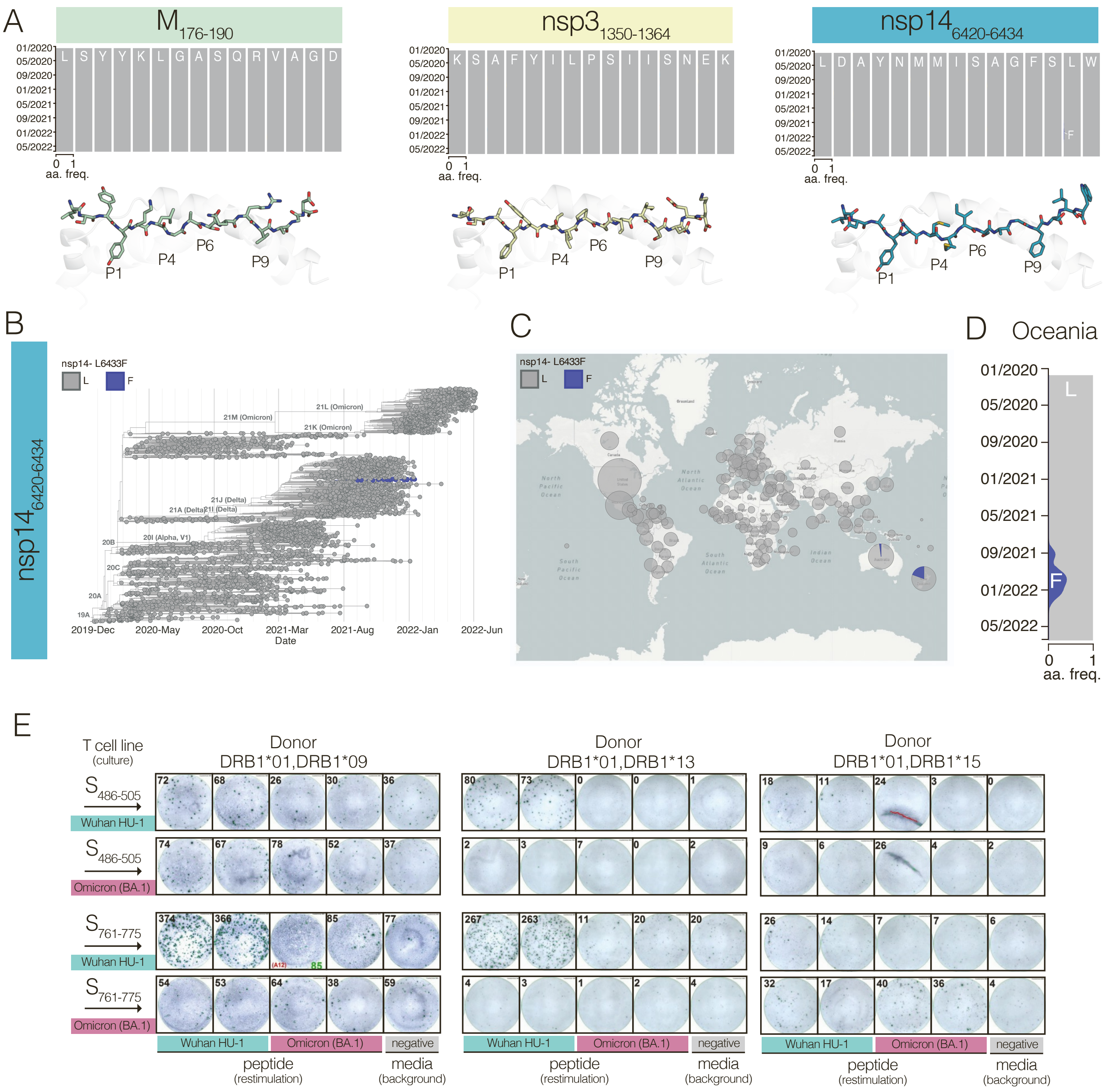
A
M176-190
L S Y Y K L G A S Q R V A G D
nsp3 1350-1364
K S A F Y I L P S I I S N E K
nsp14 6420-6434
L D A Y N M M I S A G F S L W
01/2020
05/2020
09/2020
01/2021
05/2021
09/2021
01/2022
05/2022
0 1
aa. freq.
P1
P4
P6
P9
B
nsp14 6420-6434
nsp14- L6433F
L
F
21L (Omicron)
21M (Omicron)
21K (Omicron)
21J (Delta)
21A (Delta)
21I (Delta)
20B
20I (Alpha, V1)
20C
20A
19A
2019-Dec
2020-May
2020-Oct
2021-Mar
2021-Aug
2022-Jan
2022-Jun
Date
C
nsp14- L6433F
L
F
D
Oceania
L
F
E
T cell line
(culture)
Donor
DRB1*01,DRB1*09
DRB1*01,DRB1*13
DRB1*01,DRB1*15
S486-505
S761-775
Wuhan HU-1
Omicron (BA.1)
negative
peptide
(restimulation)
media
(background)

Figure S4 - Analysis of SARS-CoV-2 variant impact on epitope sequence and immunogenicity. A) Cumulative genotypic frequency plots of amino acid usage of peptide epitope residue positions over time (Jan 2020 – June 2022) in global viral genome sequences for non-Spike epitopes crystallized (GISAID database) - HLA-DR1-$M_{176-190}$ (left), -$nsp3_{1350-1364}$ (middle) and -$nsp14_{6420-6434}$ (right). For each, the reference amino acid (aa) usage (Wuhan HU-1 strain) is colored grey. No mutations associated with Omicron (BA.1) were observed in global data. Other mutations present in other lineages are colored blue. Plots generated using the Nextstrain ncov portal. **B)** Phylogeny tree of SARS-CoV-2 sequences in the GISAID global database highlighting a lineage of 21J (Delta) containing the nsp14-L6433F dimorphism contained within the HLA-DR1-$nsp14_{6420-6434}$ epitope. **C)** Geographical map highlighting the global distribution of the nsp14-L6433F dimorphism isolated to Oceania. Pie slices colored as indicated inset. **D)** Cumulative genotypic frequency plot of amino acid residue usage at position nsp14-L6433F over time (Jan 2020 – June 2022) in the GISAID Oceania dataset alone highlighting the nsp14-L6433F dimorphism occurrence over time within this population. **E)** IFN-γ ELISpot images of data described and plotted in Fig 5C) for all three HLA-DR1^{+} donors. Donor DRB1*01, DRB1*13 is replicated from Fig 5D to allow ease of comparison with other donors. Peptides used in cultured T cell line are across rows and the variant peptides used for restimulation (overnight ELISpot assay) indicated in columns.

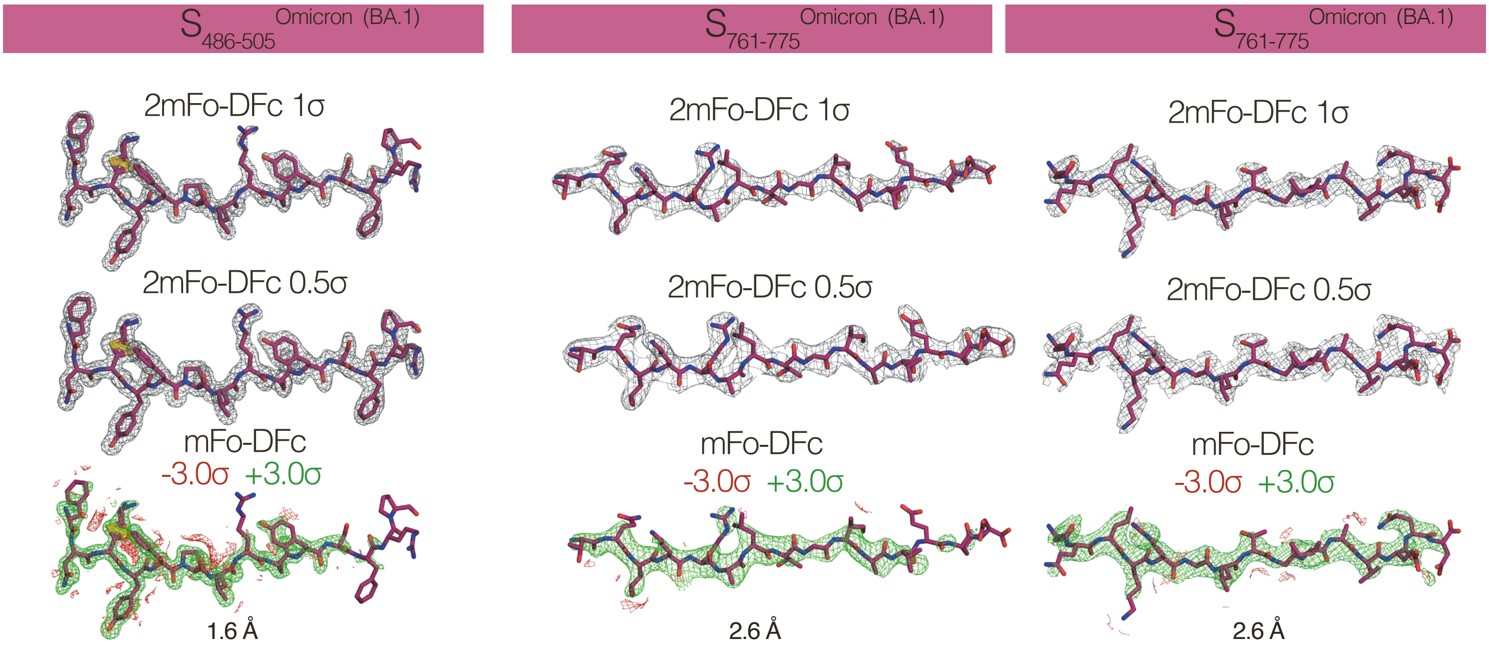

$S_{486-505}$ Omicron (BA.1)
2mFo-DFc 1σ
2mFo-DFc 0.5σ
mFo-DFc
-3.0σ +3.0σ
1.6 Å
$S_{761-775}$ Omicron (BA.1)
2mFo-DFc 1σ
2mFo-DFc 0.5σ
mFo-DFc
-3.0σ +3.0σ
2.6 Å
$S_{761-775}$ Omicron (BA.1)
2mFo-DFc 1σ
2mFo-DFc 0.5σ
mFo-DFc
-3.0σ +3.0σ
2.6 Å

Figure S5 – Electron density maps and omit map analysis of HLA-DR1-S$_{486-505}$ $^{Omicron\ (BA.1)}$ and -S$_{761-775}$ $^{Omicron\ (BA.1)}$. Electron density and omit map analysis as described in FigS2 for HLA-DR1-S$_{486-505}$ $^{Omicron\ (BA.1)}$ (left) and -S$_{761-775}$ $^{Omicron\ (BA.1)}$ in the two registers observed in the asymmetric unit (middle & right). 2mFo-DFc contoured at 1.0 σ (top), 0.5 σ (middle) and omit map mFo-DFc difference map (bottom) are shown for each. In each, peptide is shown as stick representation, colored with C=indicated inset, N=blue, O=red. 2mFo-DFc maps are presented as grey mesh. For omit map analysis, a two macro-cycle refinement including simulated annealing was performed using *phenix.refine* in the absence of peptide atoms. A resultant mFo-DFc map was calculated from the omit map refinement. For each omit map, positive difference map peaks (green mesh, +3.0 σ) and negative difference map peaks (red mesh, -3.0 σ) are shown overlayed with the final peptide co-ordinates which were absent in their calculation.

	HLA-DR1-$S_{486-505}$	HLA-DR1-$S_{511-530}$	HLA-DR1-$S_{761-775}$	HLA-DR1-$M_{176-190}$	HLA-DR1-$nsp3_{1350-1364}$	HLA-DR1-$nsp14_{6420-6434}$	HLA-DR1-$S_{486-505}^{(BA.1)\ omicron}$	HLA-DR1-$S_{761-775}^{(BA.1\ Omicron)}$
Wavelength	0.9795	0.9795	0.9795	0.9795	0.9795	0.9795	0.9795	0.9795
Resolution range	42.43 - 1.84 (1.90 - 1.84)	52.47 - 1.42 (1.47 - 1.42)	91.56 - 2.54 (2.63 - 2.54)	47.29 - 2.26 (2.34 - 2.26)	55.11 - 2.20 (2.28 - 2.20)	59.80 - 1.64 (1.70 - 1.64)	50.16 - 1.64 (1.70 - 1.64)	48.67 - 2.60 (2.69 - 2.60)
Space group	P 2_1 2_1 2	P 6_5 2 2	C 2 2 2_1	C 2 2 2_1	P 2_1 2_1 2_1	P 6_5 2 2	P 2_1 2_1 2	P 3_2 2 1
Unit cell	89.29, 136.12, 41.18 90°, 90°, 90°	60.58, 60.58, 419.25 90°, 90°, 120°	113.02, 156.12, 164.85 90°, 90°, 90°	113.37, 156.77, 165.45 90°, 90°, 90°	60.23, 95.49, 202.43 90°, 90°, 90°	61.25, 61.25, 418.59 90°, 90°, 120°	91.03, 120.19, 40.88 90°, 90°, 90°	148.19, 148.19, 129.07 90°, 90°, 120°
Total reflections	580210 (60381)	3420039 (337712)	1721918 (174963)	946681 (92623)	1678731 (154626)	1271644 (126014)	754062 (76271)	1062657 (109285)
Unique reflections	44533 (4359)	88010 (8568)	48332 (4754)	69030 (6779)	60213 (5910)	58910 (5691)	55347 (5416)	50460 (4998)
Multiplicity	13.0 (13.9)	38.9 (39.4)	35.6 (36.8)	13.7 (13.7)	27.9 (26.2)	21.6 (22.1)	13.6 (14.1)	21.1 (21.8)
Completeness (%)	99.96 (99.98)	99.89 (99.63)	99.69 (99.22)	99.81 (98.96)	99.64 (98.89)	99.52 (98.07)	98.92 (97.87)	99.52 (99.09)
Mean I/sigma(I)	10.48 (0.80)	16.93 (0.73)	8.05 (0.88)	16.05 (0.50)	9.39 (0.86)	24.06 (1.08)	6.20 (0.59)	5.93 (0.56)
Wilson B-factor	36.33	20.77	53.04	62.92	44.49	32.77	19.44	55.03
R_{merge}	0.133 (3.046)	0.116 (3.341)	0.808 (8.588)	0.0996 (3.099)	0.345 (4.163)	0.0540 (2.341)	0.341 (3.678)	0.358 (2.637)
R_{meas}	0.138 (3.162)	0.117 (3.383)	0.820 (8.707)	0.104 (3.219)	0.352 (4.244)	0.0553 (2.396)	0.354 (3.815)	0.367 (2.699)
R_{pim}	0.0385 (0.842)	0.0187 (0.530)	0.137 (1.431)	0.0279 (0.867)	0.0663 (0.818)	0.0120 (0.502)	0.0949 (1.005)	0.0793 (0.571)
$CC_{1/2}$	0.999 (0.368)	1.000 (0.663)	0.994 (0.356)	0.998 (0.378)	0.998 (0.717)	0.999 (0.859)	0.995 (0.340)	0.992 (0.522)
CC*	1.000 (0.734)	1.000 (0.893)	0.999 (0.725)	1.000 (0.741)	1.000 (0.914)	1.000 (0.961)	0.999 (0.712)	0.998 (0.828)
Reflections used in refinement	44522 (4358)	87967 (8565)	48190 (4717)	68956 (6753)	60029 (5859)	58711 (5643)	55327 (5411)	50437 (5001)
Reflections used for R-free	2182 (217)	4405 (425)	2452 (219)	3406 (315)	2972 (280)	2865 (281)	2731 (283)	2554 (245)
R_{work}	0.193 (0.375)	0.187 (0.310)	0.195 (0.355)	0.198 (0.383)	0.207 (0.368)	0.194 (0.411)	0.182 (0.325)	0.192 (0.293)
R_{free}	0.231 (0.427)	0.209 (0.319)	0.239 (0.366)	0.240 (0.408)	0.240 (0.380)	0.226 (0.445)	0.207 (0.346)	0.237 (0.332)
CC_{work}	0.967 (0.685)	0.961 (0.797)	0.963 (0.673)	0.966 (0.546)	0.967 (0.822)	0.962 (0.883)	0.969 (0.657)	0.956 (0.658)
CC_{free}	0.967 (0.643)	0.963 (0.757)	0.955 (0.665)	0.948 (0.426)	0.954 (0.757)	0.963 (0.833)	0.965 (0.628)	0.931 (0.546)
Number of non-hydrogen atoms	3451	3608	9751	9655	6591	3447	3686	9714
- macromolecules	3207	3239	9546	9546	6363	3135	3228	9465
- ligands	48	82	40	20	32	84	133	98
- solvent	196	287	165	89	196	228	325	151
Protein residues	388	388	1162	1163	772	382	388	1156
RMS_{bonds}	0.016	0.015	0.004	0.005	0.006	0.012	0.005	0.002
RMS_{angles}	1.36	1.44	0.75	0.79	0.82	1.26	0.81	0.60
Ramachandran favored (%)	98.17	98.17	98.08	98.08	97.89	98.66	97.91	97.89

Ramachandran allowed (%)	1.83	1.83	1.92	1.83	2.11	1.34	2.09	2.11
Ramachandran outliers (%)	0.00	0.00	0.00	0.09	0.00	0.00	0.00	0.00
Rotamer outliers (%)	0.28	0.00	0.95	0.48	0.00	0.00	0.00	0.10
Clashscore	6.89	3.81	4.73	3.20	4.86	4.42	3.90	4.69
Average B-factor	40.93	30.06	56.11	74.35	62.96	46.47	25.80	65.65
- macromolecules	40.80	29.03	56.21	74.44	63.23	46.28	24.47	65.66
- ligands	47.19	48.90	64.86	83.85	71.48	53.04	42.15	83.45
- solvent	41.59	36.33	48.48	62.01	52.81	46.62	32.38	53.89
Number of TLS groups	10	5	36	33	19	5	10	34

Statistics for the highest-resolution shell are shown in parentheses.

Table S1 – Data collection and refinement statistics

	**HLA-DR1-$S_{486-505}$**	**HLA-DR1-$S_{511-530}$**	**HLA-DR1-$S_{761-775}$**	**HLA-DR1-$M_{176-190}$**	**HLA-DR1-$nsp3_{1350-1364}$**	**HLA-DR1-$nsp14_{6420-6434}$**	**HLA-DR1-$S_{486-505}^{(BA.1)\ omicron}$**	**HLA-DR1-$S_{761-775}^{(BA.1\ Omicron)}$**
Peptide sequence	FNCYFPLQSYGF QPTNGVGY	VVLSFELLHAPA TVCGPKKS	TQLNRALTGIAV EQD	LSYYKLGASQRV AGD	KSAFYILPSIISNE K	LDAYNMMISAGF SLW	FNCYFPLRSYSF RPTYGVGH	TQLKRALTGIAV EQD
Protein	S	S	S	M	nsp3	nsp14	S	S
Viral variant	Wuhan HU-1	Wuhan HU-1	Wuhan HU-1	Wuhan HU-1	Wuhan HU-1	Wuhan HU-1	Omicron (BA.1)	Omicron (BA.1)
Residues	S 486-505	S 511-530	S 761-775	M 167-181	nsp3<br><br>Orf1ab 1350-1364	nsp14<br><br>Orf1ab 6420-6434	S 486-505	S 761-775
Beamline	DLS i04	DLS i04	DLS i04	DLS i04	DLS i04	DLS i04	DLS i04	DLS i04
Proposal/visit	mx20147-39	mx20147-39	mx20147-44	mx20147-39	mx29502-3	mx29502-3	mx29990-2	mx29990-2
Crystallization conditions	M MES pH 6.0 20 % PEG1500	0.1 M MES pH 7.0, 25 % PEG8000, 0.2 M $(NH_4)_2SO4$	0.1 M MES pH 5.0, 20 % PEG1500	0.1 M MES pH 5.0 20 % PEG3350	0.1 M SPG pH 4.6 25 % PEG1500	0.1 M Sodium cacodylate pH 6.5, 25 % PEG8000, 0.2 M $(NH_4)_2SO4$	0.1 M Sodium cacodylate pH 6.0,25 % PEG4000, 0.2 M $(NH_4)_2SO4$	0.1 M Tris pH 7.0, 28 % PEG8000, 0.2 M $(NH_4)_2SO4$
Cryoprotectant	+ 10 % ethylene glycol	+ 10 % ethylene glycol	+ 10 % ethylene glycol	+ 10 % ethylene glycol	+ 10 % ethylene glycol	+ 10 % ethylene glycol	+ 10 % ethylene glycol	+ 10 % ethylene glycol
Drop size	3 μL	0.4 μL	3 μL	3 μL	0.4 μL	0.4 μL	0.4 μL	0.4 μL
Crystallization experiment	Hanging drop	Sitting drop	Hanging drop	Hanging drop	Sitting drop	Sitting drop	Sitting drop	Sitting drop
PHASER model (PDB Acc. No.)	6QZC	4X5W	4X5W	4X5W	3PDO	4X5W	HLA-DR1-$S_{486-505}$	HLA-DR1-$S_{761-775}$

Table S2 – Additional p-HLA-DR1 crystallization, data collection and refinement information
