# Supplementary material for: Structural definition of HLA class II-presented SARS-CoV-2 epitopes reveals a mechanism to escape pre-existing CD4+ T cell immunity
Source: Cell Rep. 2023 Jul 19;42(8):112827. doi: 10.1016/j.celrep.2023.112827 (PMC10840515; doi:10.1016/j.celrep.2023.112827)
Supplement: Document S2. Article plus supplemental information [file mmc3.pdf]

Cell Reports

Article

Structural definition of HLA class II-presented SARS-CoV-2 epitopes reveals a mechanism to escape pre-existing CD4$^+$ T cell immunity

Graphical abstract

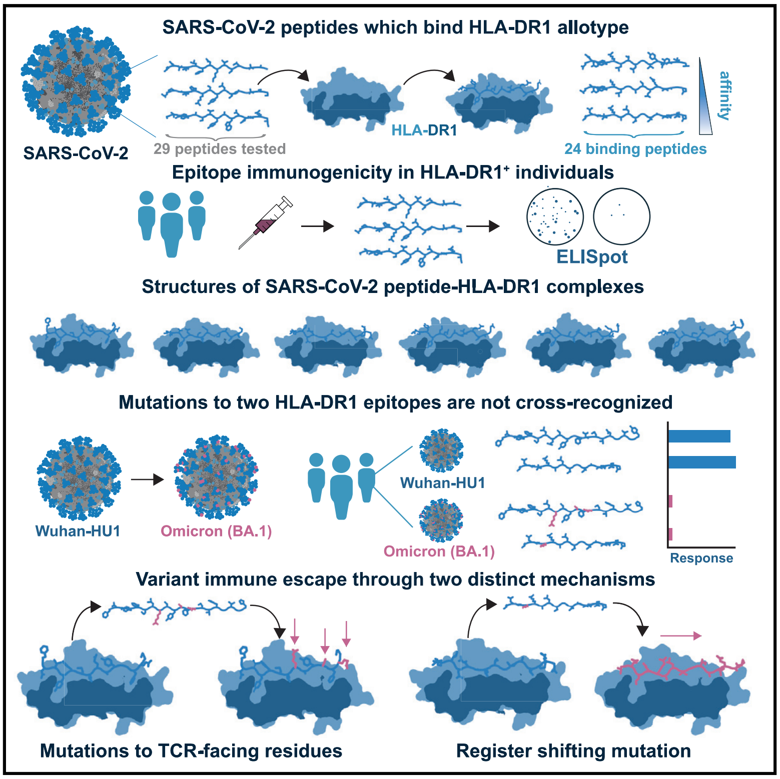

Authors

Yuan Chen, Georgina H. Mason, D. Oliver Scourfield, ..., Pierre Rizkallah, Bruce J. MacLachlan, Andrew Godkin

Correspondence

maclachlanb@cardiff.ac.uk (B.J.M.), godkinaj@cardiff.ac.uk (A.G.)

In brief

Chen et al. identify and characterize SARS-CoV-2-derived CD4$^+$ T cell epitopes restricted by the common HLA-DR1 allotype. Biophysical analyses suggest that peptide-HLA-DR1 affinity is not the key determinant of immunogenicity, and crystallographic structures of HLA-bound variant epitopes demonstrate two different mechanisms of escape from memory CD4$^+$ T cells.

Highlights

- Characterization of HLA-DR1-restricted SARS-CoV-2 peptide epitopes
- Epitope immunogenicity does not correlate with peptide affinity for HLA-DR1
- Structures of six p-HLA-DR1 complexes show that low-affinity peptides bind canonically
- BA.1 mutations evade T cells via two distinct mechanisms including register shifting

Chen et al., 2023, Cell Reports *42*, 112827
August 29, 2023 © 2023 The Authors.
https://doi.org/10.1016/j.celrep.2023.112827

CellPress

Cell Reports

CellPress
OPEN ACCESS

Article

Structural definition of HLA class II-presented SARS-CoV-2 epitopes reveals a mechanism to escape pre-existing CD4+ T cell immunity

Yuan Chen,[1,2] Georgina H. Mason,[1,2] D. Oliver Scourfield,[1,2] Alexander Greenshields-Watson,[1,2,5] Tracey A. Haigh,[3] Andrew K. Sewell,[1,2] Heather M. Long,[3] Awen M. Gallimore,[1,2] Pierre Rizkallah,[1,2] Bruce J. MacLachlan,[1,2,6,*] and Andrew Godkin[1,2,4,6,7,*]

[1]Division of Infection and Immunity, School of Medicine, Cardiff University, Cardiff CF14 4XN, UK
[2]Systems Immunity University Research Institute, School of Medicine, Cardiff University, Cardiff CF14 4XN, UK
[3]Institute of Immunology and Immunotherapy, University of Birmingham, Birmingham B15 2TT, UK
[4]Department of Gastroenterology & Hepatology, University Hospital of Wales, Cardiff CF14 4XW, UK
[5]Present address: Department of Statistics, University of Oxford, Oxford OX1 3LB, UK
[6]These authors contributed equally
[7]Lead contact
*Correspondence: maclachlanb@cardiff.ac.uk (B.J.M.), godkinaj@cardiff.ac.uk (A.G.)
https://doi.org/10.1016/j.celrep.2023.112827

SUMMARY

CD4+ T cells recognize a broad range of peptide epitopes of severe acute respiratory syndrome coronavirus 2 (SARS-CoV-2), which contribute to immune memory and limit COVID-19 disease. We demonstrate that the immunogenicity of SARS-CoV-2 peptides, in the context of the model allotype HLA-DR1, does not correlate with their binding affinity to the HLA heterodimer. Analyzing six epitopes, some with very low binding affinity, we solve X-ray crystallographic structures of each bound to HLA-DR1. Further structural definitions reveal the precise molecular impact of viral variant mutations on epitope presentation. Omicron escaped ancestral SARS-CoV-2 immunity to two epitopes through two distinct mechanisms: (1) mutations to TCR-facing epitope positions and (2) a mechanism whereby a single amino acid substitution caused a register shift within the HLA binding groove, completely altering the peptide-HLA structure. This HLA-II-specific paradigm of immune escape highlights how CD4+ T cell memory is finely poised at the level of peptide-HLA-II presentation.

INTRODUCTION

Recovery from COVID-19, as a result of infection with the causative virus severe acute respiratory syndrome coronavirus 2 (SARS-CoV-2), is associated with a concerted immune response characterized by both CD8+ and CD4+ T cells[1] along with neutralizing antibodies.[2] Administration of various vaccines induces similar immune memory to the target antigen, currently the host entry protein Spike.[3–6] Maintaining durable immunity within individuals that recognizes current and future global SARS-CoV-2 variants remains the key goal in preventing further impact caused by the circulating virus.

Highly activated CD4+ and CD8+ T cells were seen with systemic inflammation and more severe disease in patients hospitalized due to COVID-19.[7] However, in individuals where cellular immunity was induced early after infection, the disease appeared less severe,[8] and T cell immunity against multiple SARS-CoV-2 viral proteins is observed in nearly all patients recovered from COVID-19.[9] SARS-CoV-2-specific central memory (CCR7+, CD45RA−) T_H1 CD4+ T cells persist in patients recovered from COVID-19 with an estimated half-life of ~200 days; T_H2 and T_H17 cells do not contribute significantly to this memory pool.[10] Although there has been a clear focus on measured serological responses as markers for protective immunity, recent studies have highlighted the correlation between SARS-CoV-2-specific IFN-γ^+ T cell responses and protection from reinfection over a 6 month follow-up period, irrespective of the levels of antibodies.[11]

Multiple groups have characterized targets of SARS-CoV-2-specific T cells, through computational prediction,[12,13] experimental peptide mapping,[9,14–17] humanized murine models,[18] and library display approaches.[19] These have revealed cellular immunity across the breadth of the SARS-CoV-2 proteome. Furthermore, in virus-inexperienced early-pandemic cohorts, pre-existing immunity to SARS-CoV-2[14] has been attributed to cross-reactivity with endemic seasonal human betacoronaviruses, particularly to more conserved regions of the Spike protein (S2 domain)[20] and conserved proteins of the replication-transcription complex (RTC).[21]

Despite comprehensive whole-viral-proteome analysis of both CD4+ and CD8+ T cell epitopes of SARS-CoV-2, detailed analyses of specific epitopes, particularly those presented on the human leukocyte antigen class II (HLA-II) platform to CD4+ T cells, are limited. Focused structural and biophysical analyses of individual CD4+ T cell epitopes in other anti-viral immune responses, such as influenza A[22,23] and HIV,[24] contribute enormously to understanding the immune response. Moreover, such data have

This is an open access article under the CC BY license (http://creativecommons.org/licenses/by/4.0/).

provided mechanistic understanding to viral escape—particularly in the CD8$^+$ T cell/HLA-I axis—including escape from SARS-CoV-2[25–28] and HIV.[29,30] Given the different properties of HLA-II-peptide binding, the mechanisms of escape may not necessarily be translatable from their cytotoxic T cell counterparts. Characteristic of the open HLA-II binding groove, peptides presented to CD4$^+$ T cells exhibit a varied longer length (~12–20 amino acids)[31] and are anchored by a central nonamer core binding region, leaving peptide flanking residues (PFRs) at the N and C termini of the core, which may also impact immunogenicity.[32] As a result, accurately pinpointing how epitopes are presented as peptide cargo is challenging both experimentally and computationally, especially with respect to determining the core "register" of presentation.

Utilizing iterative peptide/HLA-II display libraries covering the SARS-CoV-2 genome, Obermair et al. identified binding ligands for a series of HLA types.[19] This approach allowed an exploration of the impact of mutants on ligand/epitope display. It is known that a mutation to either a key "downward-facing" residue that contributes to peptide binding to the HLA groove or an "upward-facing" amino acid side chain that engages the T cell receptor (TCR) can lead to loss of T cell activation.[33] For instance, in HIV, escape from CD4$^+$ T cell recognition of immunodominant viral epitopes has been demonstrated via mutations that disrupt TCR recognition while maintaining HLA-II binding.[34] Although not clearly proven, it is also plausible that mutations in epitopes might create new ligand binding registers, hence theoretically shifting the TCR upward-facing residues.[19] Structural explanations of such molecular mechanisms, however, are yet to be described in the CD4$^+$ T cell/HLA-II axis.

In this study, we set out at the start of the pandemic to identify and characterize peptide epitopes of SARS-CoV-2 that could be used to explore the importance of CD4$^+$ T cell responses in antiviral immunity. Using HLA-DRA1*0101, B1*0101 (HLA-DR1) as a model allotype, we first assessed the ability of 29 SARS-CoV-2-derived candidate peptides to bind HLA-DR1 and characterized their immunogenicity *in vitro* using blood from a range of HLA-DR1-positive and -negative donors. Details of these CD4$^+$ T cell responses would allow insight into both epitope selection and immunodominance and the mechanism(s) behind loss of T cell responses that independently associate with recurrent infection. We describe the structures of six epitopes enabling unequivocal definition of epitope core region and register. As the pandemic unfolded and variant viruses emerged, we analyzed their effect on bound peptide cargo. Corroborating further structural data of Omicron variant-mutated peptide-HLA-DR1 complexes with *in vitro* binding and immunogenicity data, we identify distinct mechanisms of immune escape from T cell recognition by Omicron variant peptides, highlighting the importance of the peptide-HLA-II platform characteristics in maintaining long-lasting anti-viral immunity.

RESULTS

Identification of HLA-DR1 binding peptides from SARS-CoV-2

To characterize important HLA-II peptide epitopes of SARS-CoV-2, we aimed to provide a mechanistic understanding of their immunogenicity through the structural characterization of their HLA-II presentation using HLA-DR1 as a model allotype. Surveying the literature on CD4$^+$ T cell epitopes in either individuals who were unexposed or patients recovered/recovering from COVID-19 circa November 2020, we selected 29 peptides using evidence of immunogenicity in cohorts containing HLA-DR1 positivity combined with predicted binding to HLA-DR1 according to NetMHCIIpan[35] (Table 1). Selected candidate peptides encompassed diverse regions across the SARS-CoV-2 (Wuhan HU-1) genome, including both Spike (S)-derived and non-Spike peptides (Figure 1A). Twenty-seven peptides were synthesized as 15mers, registering the predicted 9mer core binding sequence centrally, flanked by 3-amino-acid N-terminal and C-terminal PFRs. Two further peptides were identified as HLA-DR1 epitopes through the isolation and HLA-II restriction of CD4$^+$ T cell clones, which were reactive to 20mer peptides,[36] and, as core prediction was not conclusive, $S_{486–505}$ and $S_{511–530}$ were synthesized as 20mer peptides.

Synthesized peptides were first assayed *in vitro* for the ability to bind HLA-DR1 by competitive inhibition assays.[37] The immunodominant HLA-DR1-restricted influenza A virus hemagglutinin (HA) peptide $HA_{305–319}$[38,39] was also tested as a comparator, giving an IC_{50} of 29 nM. The binding assays of the SARS-CoV-2 peptides gave rise to a wide range of measured affinities, which were divided into four groups: 6 strong binding peptides (IC_{50} < 1,000 nM), 10 weak binding peptides (1,000 < IC_{50} < 10,000 nM), 8 very weak but detectable binding peptides (IC_{50} > 10,000 nM or 10 μM), and 5 peptides with no detectable binding (despite *in silico* predictions) (Figure 1B). The Membrane (M)-derived peptide $M_{176–190}$ had the highest affinity (IC_{50} = 7.9 nM) and was the only binder stronger than our reference epitope ($HA_{305–319}$, IC_{50} = 29 nM). Of the six peptides considered strong binders, only one was Spike derived ($S_{512–526}$, IC_{50} = 662 nM). Thus, 24 of the 29 peptides tested bound HLA-DR1 and encompassed a wide range of affinities (IC_{50} = 7.9 to >10,000 nM).

Immunogenicity of HLA-DR1 binding peptides from SARS-CoV-2

To focus our analyses, the immunogenicity of each candidate peptide was assessed in short-term T cell lines derived from HLA-DR1-positive or -negative donors, whereby isolated peripheral blood mononuclear cells (PBMCs) underwent a brief 12 day culture with individual candidate peptides. Peptide-specific T cell expansion to each was assessed via overnight IFN-γ ELISpot in response to peptide restimulation (Figures 2A and 2B). Each donor was assayed for reactivity at two time points, pre- (Figure S1A) and post- (Figure S1B) vaccination, and the maximal response is summarized in Figure 2B. Peptides $S_{486–505}$ and $S_{511–530}$ were identified in a separate cohort of health-care workers[36] after the vaccination of the HLA-DR1-positive cohort and thus tested post-vaccination only.

The validity of the initial peptide selection was confirmed by the significant increase in the total number of T cell responses in HLA-DR1-positive donors (Figure 2C) (HLA-DR1-positive donors 36/87 (41%) vs. HLA-DR1-negative donors 20/135 (15%), $p < 0.01$, Fisher's exact test). Across the panel of tested peptides, response rate in HLA-DR1-positive donors to Spike peptides was higher at 23/45 (51%) than for non-Spike peptides, with 13/42 (31%), possibly indicative of vaccination. As

Table 1. SARS-CoV-2-derived peptides selected for analysis

Name	Sequence	Protein	Start	End	Length	Reference
$S_{1–15}$	MFVFLVLLPLVSSQC	S	1	15	15	Prakash et al.[18]
$S_{167–181}$	TFEYVSQPFLMDLEG	S	167	181	15	Peng et al.[15]
$S_{235–249}$	ITRFQTLLALHRSYL	S	235	249	15	Mateus et al. and Nelde et al.[14,16]
$S_{241–255}$	LLALHRSYLTPGDSS	S	241	255	15	Mateus et al.[14]
$S_{339–353}$	GEVFNATRFASVYAW	S	339	353	15	Mateus et al.[14]
$S_{344–358}$	ATRFASVYAWNRKRI	S	344	358	15	Mateus et al.[14]
$S_{486–505}$	FNCYFPLQSYGFQPTNGVGY	S	486	505	20	Tye et al.[36]
$S_{511–530}$	VVLSFELLHAPATVCGPKKS	S	511	530	20	Tye et al.[36]
$S_{512–526}$	VLSFELLHAPATVCG	S	512	526	15	Peng et al.[15]
$S_{749–763}$	CSNLLLQYGSFCTQL	S	749	763	15	Peng et al.[15]
$S_{761–775}$	TQLNRALTGIAVEQD	S	761	775	15	Mateus et al.[14]
$S_{818–832}$	IEDLLFNKVTLADAG	S	818	832	15	Mateus et al.[14]
$S_{866–880}$	TDEMIAQYTSALLAG	S	866	880	15	Peng et al.[15]
$S_{990–1004}$	EVQIDRLITGRLQSL	S	990	1,004	15	Mateus et al.[14]
$S_{1015–1029}$	AAEIRASANLAATKM	S	1,015	1,029	15	Peng et al.[15]
$E_{56–70}$	FYVYSRVKNLNSSRV	E	56	70	15	Peng et al. and Nelde et al.[15,16]
$M_{176–190}$	LSYYKLGASQRVAGD	M	176	190	15	Peng et al., Nelde et al., and Prakash et al.[15,16,18]
$N_{49–63}$	TASWFTALTQHGKED	N	49	63	15	Nelde et al.[16]
$N_{108–122}$	WYFYYLGTGPEAGLP	N	108	122	15	Nelde et al.[16]
$N_{127–141}$	KDGIIWVATEGALNT	N	127	141	15	Nelde et al.[16]
$N_{224–238}$	LDRLNQLESKMSGKG	N	224	238	15	Nelde et al.[16]
$N_{328–342}$	GTWLTYTGAIKLDDK	N	328	342	15	Nelde et al.[16]
$nsp3_{1350–1364}$	KSAFYILPSIISNEK	orf1ab (nsp3)	1,350	1,364	15	Prakash et al.[18]
$nsp6_{3801–3815}$	NRYFRLTLGVYDYLV	orf1ab (nsp6)	3,801	3,815	15	Mateus et al.[14]
$nsp12_{5019–5033}$	PNMLRIMASLVLARK	orf1ab (nsp12)	5,019	5,033	15	Prakash et al.[18]
$nsp14_{6420–6434}$	LDAYNMMISAGFSLW	orf1ab (nsp14)	6,420	6,434	15	Prakash et al.[18]
$ORF6_{13–27}$	EILLIIMRTFKVSIW	ORF6	13	27	15	Prakash et al.[18]
$ORF8_{3–17}$	FLVFLGIITTVAAFH	ORF8	3	17	15	Prakash et al.[18]
$ORF8_{43–57}$	SKWYIRVGARKSAPL	ORF8	43	57	15	Nelde et al.[16]

expected, not only was the overall response rate higher in HLA-DR1-positive donors, but larger magnitude cultured responses (>100 spot-forming cells [sfcs]/10,000 cultured cells) were confined to HLA-DR1-positive donors (Figure 2C).

$S_{167–181}$, $S_{761–775}$, and $S_{866–880}$ were recognized by all HLA-DR1-positive donors and may represent immunodominant Spike epitopes. $S_{167–181}$ and $S_{761–775}$ induced the strongest T cell expansions (Figure 2B), yet curiously, both of these peptides were designated as very weak binders to HLA-DR1 (IC_{50} > 10,000 nM; Figures 1B and 2D). The $S_{866–880}$ peptide bound with a higher affinity to HLA-DR1 (weak binder, IC_{50} = 1,149.9 nM; Figures 1B and 2D), yet the magnitude of expanded response did not exceed $S_{761–775}$. Within the tested peptides, peptide-HLA-DR1 binding affinity did not correlate with the magnitude of T cell expansion to candidate peptides (Figure 2E). There was also no correlation between percentage eluted ligand (Rank-EL) (Figure S1C) or predicted affinity (Figure S1D) and total magnitude of cultured response, highlighting how a prediction-based approach may miss important epitopes in this context.

To confirm HLA-DR1 presentation of the peptides, we generated a $CD4^+$ T cell-enriched (magnetically sorted) T cell line and used T2 cells lentivirally transduced with HLA-DR1 ($DR1^+$ only).[22] These were used as antigen-presenting cells (APCs) to stimulate cognate $CD4^+$ T cell lines, which confirmed that the very weak peptide binders, such as $S_{761–775}$, could be presented by HLA-DR1 in the context of these enriched, more protracted T cell cultures (Figures S1E and S1F). One donor (HLA-DRB1*01, -DRB1*13) responded to 50% (7/14) of the non-Spike peptides. This individual was the only donor (either HLA-DR1-positive or -negative) to respond to $M_{176–190}$, yet exhibited the strongest response measured from any donor to a non-Spike peptide. $M_{176–190}$ was the highest-affinity HLA-DR1 binder we measured from the SARS-CoV-2 peptides (Figure 1B). The HLA-DR1 restriction was again confirmed using the T2-$DR1^+$-only cells,

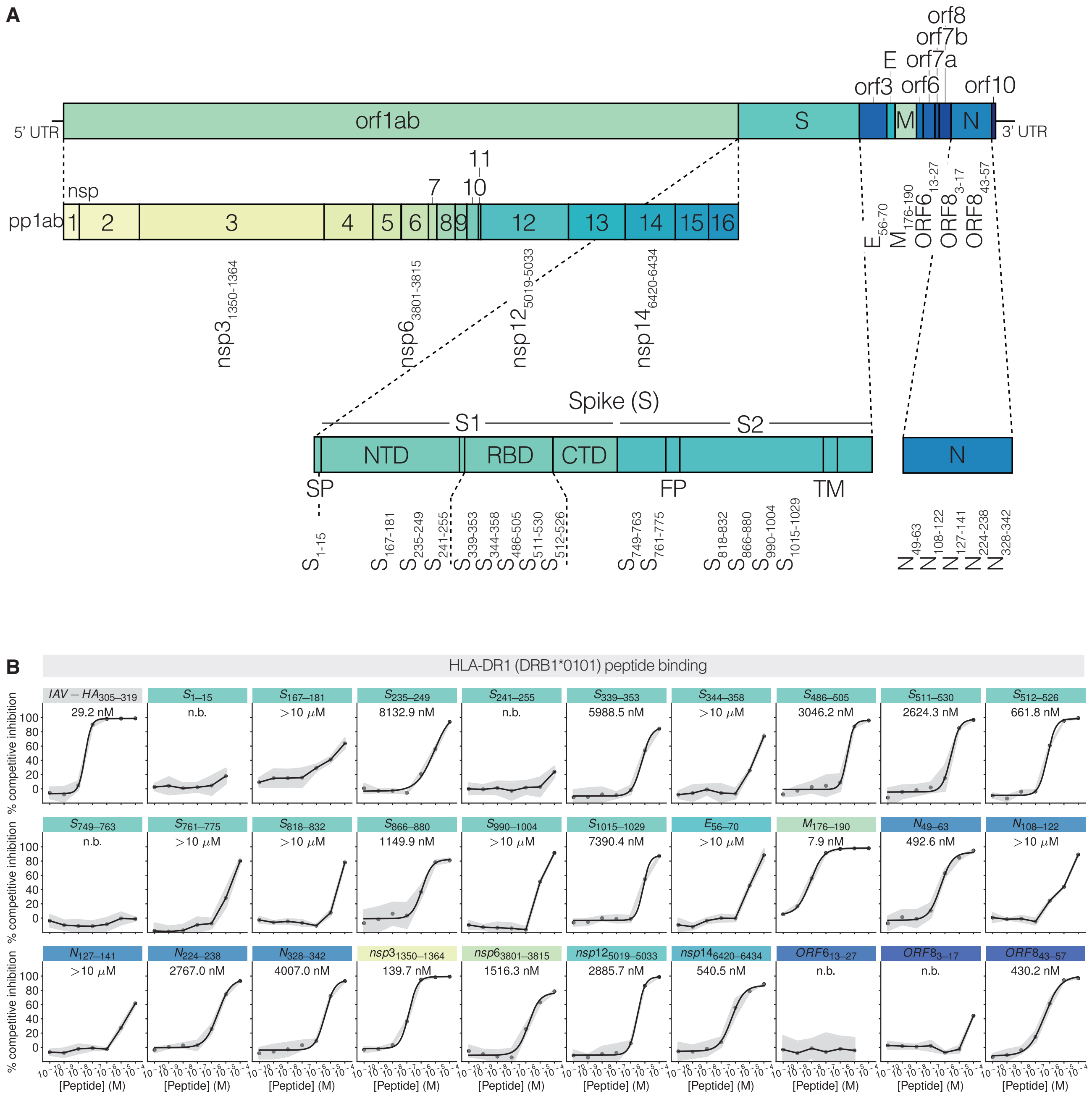

Figure 1. Identification of HLA-DR1 binding peptides derived from SARS-CoV-2

(A) A schematic overview of the SARS-CoV-2 genome highlighting peptides selected for analysis. An expanded view of Spike and the non-structural proteins produced from orf1ab (nsp1 to nsp16) is shown. S, Spike; E, Envelope; M, Membrane; N, Nucleocapsid; orf, open reading frame; nsp, non-structural protein; and UTR, untranslated region. Within Spike: SP, signal peptide; NTD, N-terminal domain; RBD, receptor binding domain; CTD, C-terminal domain; FP, fusion peptide; and TM, transmembrane domain.

(B) Peptide-HLA-DR1 binding curves for each peptide as determined by competitive inhibition assay. $HA_{305–319}$ was used as a comparative control. Data representative of n = 3 independent experiments, each with n = 3 technical replicates. Data presented as mean percentage inhibition (circles) with standard deviation (shaded area) shown as error from n = 3 technical replicates. An IC_{50} value defining peptide-HLA-DR1 binding affinity (inset) is shown where fitting resulted in values of <10 μM (curve fit, black line). Peptides with poor curve fit and weak binding (>10 μM) or no binding (n.b.) have mean values that are connected via straight lines.

CellPress
OPEN ACCESS

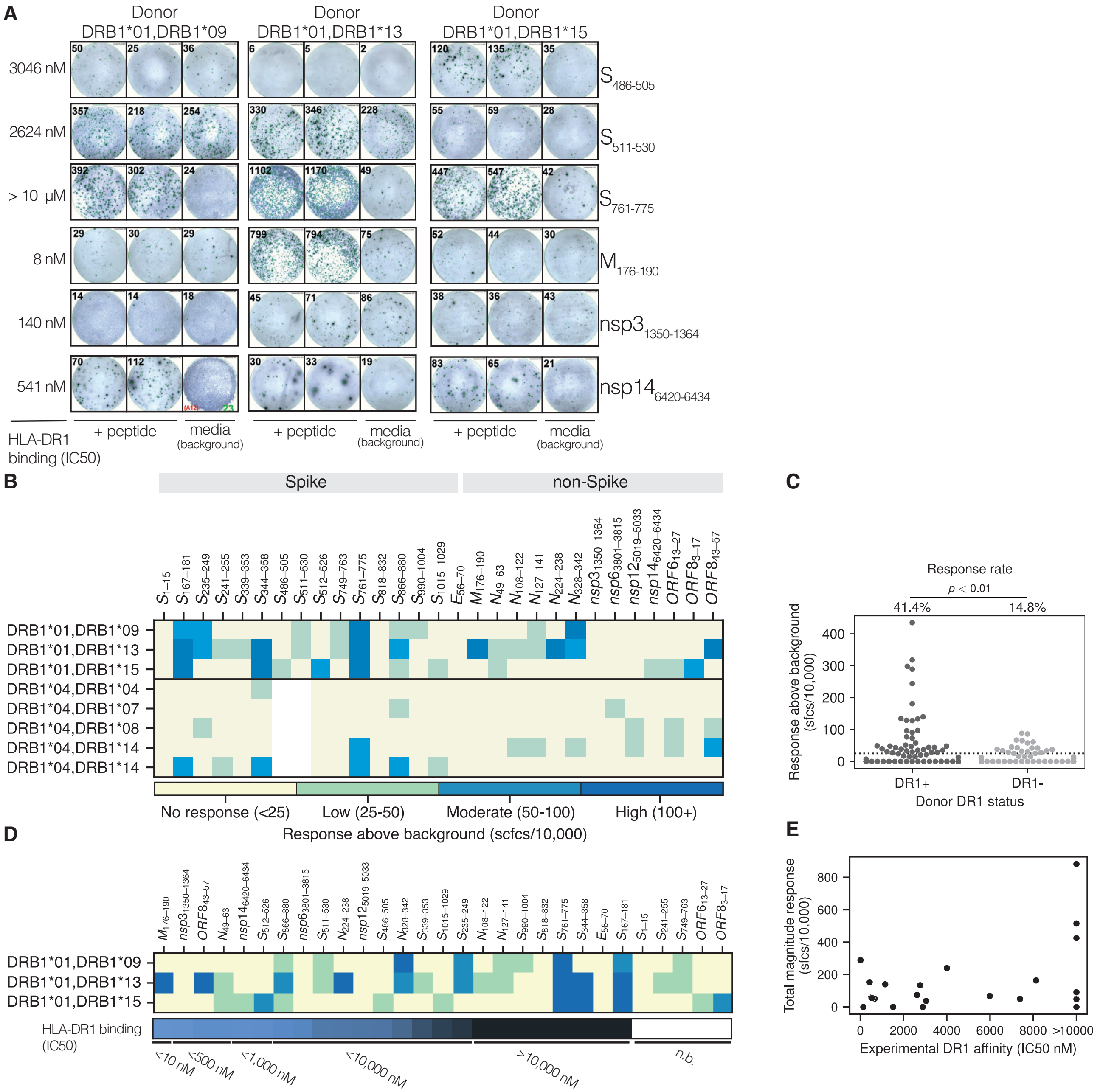

Figure 2. Immunogenicity of selected SARS-CoV-2 peptides in HLA-DR1$^+$ and HLA-DR4$^+$ donors

(A) Peptide-specific, 12-day-expanded T cell responses to SARS-CoV-2 peptides. Example test peptide (n = 2) and background (media) control IFN-γ ELISpot assays for the HLA-DR1$^+$ donors (n = 3) are shown. Examples shown are of peptides selected for later structural analyses.

(B) Heatmap summary of IFN-γ ELISpot data. Donors are grouped by HLA-DR1$^+$ (n = 3) and HLA-DR4$^+$ (n = 5). Each peptide was tested twice (n = 2) in each donor. ELISpot assays were performed in duplicate (n = 2). The maximal response from either time point is shown, with individual time points shown in Figures S1A and S1B. Responses were background subtracted (medium only), normalized to sfcs/10,000 cells, and binned into low, moderate, and high responders (cutoffs and colors indicated at bottom). $S_{486–505}$ and $S_{511–530}$ were assayed in HLA-DR1$^+$ donors (n = 3) only and post-vaccination only.

(C) Summary of maximal response of all peptides (n = 29) in all donors tested (n = 8), divided into donor DR1 status. Each marker represents a single donor maximal response to a single peptide (n = 2 ELISpot wells). Dashed line at 25 sfcs/10,000 cells was used as a cutoff for donor-peptide response. Response rate is shown as a percentage. Inset p value calculated via Fisher's exact test comparing DR1$^+$/DR4$^+$ status (groups) and the positive responses to peptides (outcomes).

(D) Heatmap summary of IFN-γ ELISpot data presented in (B) but ordered by HLA-DR1 binding affinity (IC_{50}) as determined in Figure 1B: strongest affinity (left) to weakest (right). HLA-DR1$^+$ donor data only are shown.

(E) Scatterplot summary of total magnitude response to each peptide (summated maximal response by each donor for each peptide) in HLA-DR1$^+$ donors (n = 3) against HLA-DR1 binding affinity (IC_{50}) as determined in Figure 1B. No correlation was observed.

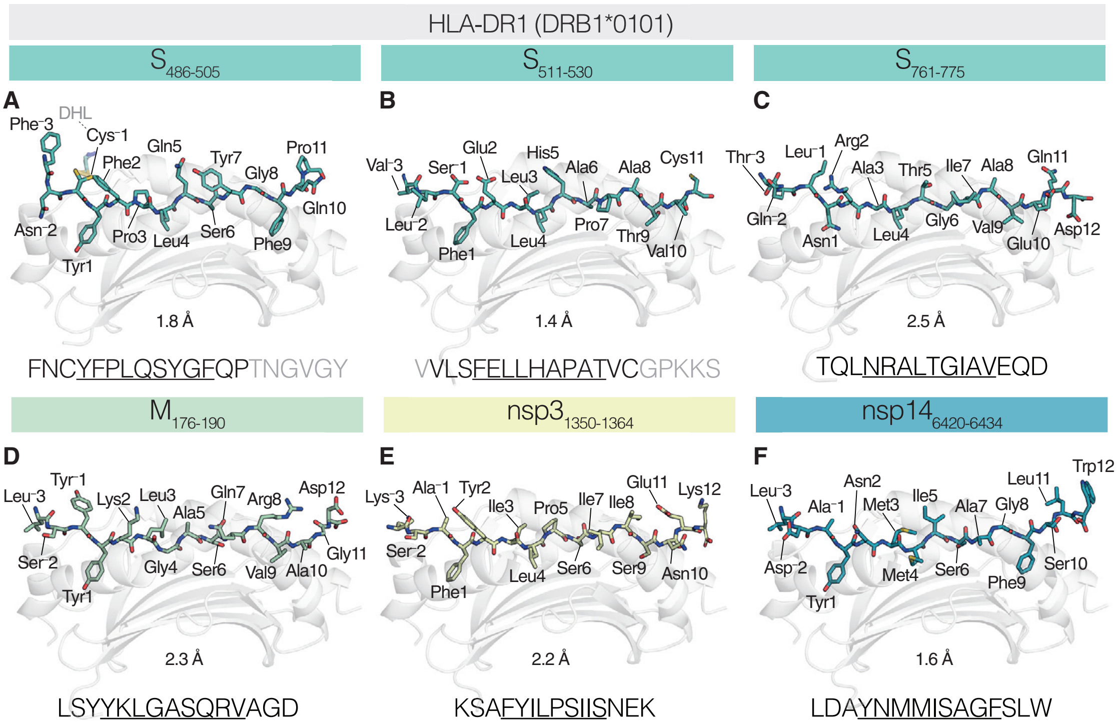

Figure 3. Structural definition of SARS-CoV-2-derived HLA-DR1 epitopes

Structural overview of HLA-DR1-$S_{486–505}$ (A), -$S_{511–530}$ (B), -$S_{761–775}$ (C), -$M_{176–190}$ (D), -nsp$3_{1350–1364}$ (E), and -nsp$14_{6420–6434}$ (F). In each, the HLA-DR1 peptide binding groove (light gray, cartoon representation) and bound peptide cargo are shown. Each peptide is shown as sticks and colored by atom (C matches the color of the viral protein origin shown at the top; N, blue; O, red; S, yellow). Residues within the peptides are numbered according to their register position, i.e., Tyr1 is in the P1 position, and Phe^{-}3 is in the P^{-}3 position. Inset peptide amino acid sequences are shown below each, with modeled residues (black) and unmodeled residues (gray) indicated, and the core nonamer binding register is underlined. Resolution is indicated below each.

where an enriched $CD4^+$ T cell line (which had undergone three rounds of enrichment/stimulation) exhibited ~80% $M_{176–190}$ reactivity by dual expression of TNF-α and IFN-γ via intracellular cytokine staining (Figure S1G). Together, these results highlight the complexity of coupling HLA-peptide binding affinities, T cell responses, and immunodominance and predicting ligand → epitope selection for HLA-II.

Structural definition of SARS-CoV-2 epitopes presented by HLA-DR1

Of the 29 peptides studied, we focused our structural analysis on three Spike and three non-Spike peptides, reasoning that this approach would characterize epitopes relevant to vaccination/infection (Spike) and infection only (non-Spike). A rationale for the selection of each is given below. All six peptide-HLA-DR1 complexes were solved via X-ray crystallography at resolutions of 1.4 to 2.5 Å (Figure 3; data collection and refinement statistics detailed in Table S1). The primary aim was to define the core binding motif of each peptide, which determines the presented peptide's binding register. We used unbiased omit map analysis to affirm binding registers (Figure S2). To consider each complex in the context of HLA-DR1 binding "fit," below we compare each to eluted ligand (EL) enrichment or binding affinity (BA) motifs described by NetMHCIIpan-4.1 data.[35] Concurrently, the definition of the core binding motif allowed for the assignment of outward-facing epitope positions, which are typically contacted by TCRs.[40,41]

$S_{486–505}$: FNCYFPLQSYGFQPTNGVGY

$S_{486–505}$-reactive $CD4^+$ T cell clones restricted to HLA-DR1 have been isolated from individuals recovering from COVID-19 by ourselves[36] and others.[42] $S_{486–505}$ bound HLA-DR1 at low affinity (IC_{50} = 3,046 nM; Figures 1B and 2D). The refolded complex was crystallized and diffracted to a resolution of 1.8 Å. HLA-DR1-$S_{486–505}$ was solved in space group P 2_1 2_1 2 containing a single copy in the asymmetric unit (Figure 3A). HLA-DR1 bound $S_{486–505}$ via the register FNCYFPLQSYGFQPTNGVGY (core residues underlined). Binding in this register satisfied 3/4 anchor residue preferences for HLA-DR1, incorporating the favored P1-Tyr, P4-Leu, and P6-Ser. Deviation from the motif ideals was observed at P9 by incorporation of P9-Phe, which prefers shorter aliphatic residues. Within the core, this register placed P2-Phe, P5-Gln, and P7-Tyr as potential contact points for TCR recognition.

A free cysteine within the peptide did not negatively affect HLA-DR1-$S_{486–505}$ refolding; however, a strong positive peak in the Fo-Fc map at the P^{-}1-Cys side chain indicated additional density capping the reactive free thiol. A covalently conjugated cystamine, likely from reaction with cysteamine (a component of the refolding solution), modeled and refined well into this

density. The cysteamine, however, did not affect peptide-HLA-DR complexation due to its outward-facing orientation.

$S_{511–530}$: VVLSFELLHAPATVCGPKKS

Studies using peptides encompassing $S_{511–530}$ have identified the region to contain a potential CD4$^+$ T cell epitope in cohorts of individuals recovered from COVID-19 (3%–22% of individuals responded).[15,17,43] We have recently isolated CD4$^+$ T cell clones reactive to $S_{511–530}$ and confirmed HLA-DR1 restriction via activation to HLA-matched allogeneic lymphoblastoid cell lines (LCLs).[36] CD4$^+$ T cell clones restricted to HLA-DR1 have also been identified by others.[42,44] $S_{511–530}$ was designated a weak binder (IC_{50} = 2,624 nM; Figure 1B). The structure of HLA-DR1-$S_{511–530}$ was solved to 1.4 Å resolution in space group P 6_5 2 2 (Figure 3B).

With a defined core of VVLSFELLHAPATVCGPKKS, $S_{511–530}$ best satisfied the HLA-DR1 binding motif within all presented peptide-HLA-DR1 complexes, where P1-Phe, P4-Leu, and P6-Ala are all the most enriched amino acids at these positions within both the EL and the BA motif. At P9, P9-Thr is enriched in the BA motif but not EL data. Despite satisfying BA/EL motifs, $S_{511–530}$ exhibited a modest affinity for HLA-DR1 (~2,600 nM). The presence of Cys11 in the C-terminal PFR did not affect refold or crystallization ability, and additional density indicating thiol capping was not present as observed in HLA-DR1-$S_{486–505}$. Within the core epitope, $S_{511–530}$ exhibits diverse physicochemical properties at TCR-facing positions: acidic P2-Glu, a short aliphatic P6-Ala, P7-Pro and P8-Ala stretch, and the basic P5-His, which shrouded over P3-Leu to form a central charge within the epitope core.

$S_{761–775}$: TQLNRALTGIAVEQD

$S_{761–775}$ was an immunogenic CD4$^+$ T cell epitope in healthy unexposed individuals (3/14; 21%), of which 5/14 donors possessed the DRB1*0101 allele.[14] More recently, $S_{761–775}$ was identified as the most immunogenic peptide to induce CD4$^+$ T cell reactivity *ex vivo*, including in subjects with no obvious history of virus exposure.[45] Despite very weak binding to HLA-DR1 *in vitro* (IC_{50} > 10 μM; Figure 1B), not only were T cell responses to $S_{761–775}$ observed in all three of our DR1$^+$ donors (Figures 2A and 2B), but the peptide bound and was crystallized with HLA-DR1. The structure of DR1-$S_{761–775}$ was solved to 2.5 Å resolution in space group C 2 2 2_1 containing three copies in the asymmetric unit (Figure 3C).

$S_{761–775}$ bound HLA-DR1 via the register TQLNRALTGIAVEQD, which placed an unfavorable Asn at P1 while placing favorable residues at all other anchor positions: P4-Leu, P6-Gly, and P9-Val. Thus, lower compatibility of P1-Asn for the P1 pocket, with preferences for larger bulky side chains, was the most likely contributor to the observed weak affinity of $S_{761–775}$ for HLA-DR1 (weakest of all crystallized peptide-HLA-DR1 complexes) when considering binding motifs. Outward-facing residues across the core and PFRs were very diverse (polar, aliphatic, and charged).

$M_{176–190}$: LSYYKLGASQRVAGD

$M_{176–190}$ was initially identified in convalescent (21/22 individuals; 95%)[16] and fully recovered (16/34 individuals)[15] patient cohorts. $M_{176–190}$ has confirmed restriction to DRB1*0101 in humanized mice,[18] using DRB1*0101-expressing APCs[46] and via HLA-DR1-$M_{176–190}$ tetramers.[47] $M_{176–190}$ is therefore one of the best-characterized CD4$^+$ T cell epitopes for SARS-CoV-2 in the Immune Epitope Database (IEDB). In our study, $M_{176–190}$ had the highest affinity for HLA-DR1 assayed (IC_{50} = 8 nM; Figure 1B). The structure of HLA-DR1-$M_{176–190}$ was solved at 2.3 Å resolution in space group C 2 2 2_1 with three copies in the asymmetric unit (Figure 3D).

HLA-DR1 bound $M_{176–190}$ via the register LSYYKLGASQRVAGD, which is in line with previous *in vitro* data determining a minimal epitope for reactive CD4$^+$ T cells.[46,47] This register placed the archetypal aromatic P1-Tyr into the P1 pocket as well as the favored P6-Ser and P9-Val. P4-Gly represented the only mismatch from mooted motif ideals. Outward TCR-facing residues were most varied at the N terminus (P$^-$1-Try and P2-Lys) and the C terminus (P8-Arg). In contrast, the aliphatic central core (P4-Gly and P5-Ala) maintained a low profile in the groove. Despite being the highest-affinity binder, $M_{176–190}$ did not distinguish itself by best satisfying EL/BA motifs compared with other epitopes with lower affinity (particularly $S_{511–530}$).

$nsp3_{1350–1364}$: KSAFYILPSIISNEK

$nsp3_{1350–1364}$ was identified as immunogenic in humanized HLA-DR1$^+$ transgenic mice and elicited strong IFN-γ responses in individuals who were both SARS-CoV-2 experienced and HLA-DR1$^+$ (27/30; 90%), as well as healthy donors with no known exposure (10/10; 100% deemed responders).[18] As one of the highest-affinity binders for HLA-DR1 identified herein (IC_{50} = 140 nM; Figure 1B) the structure of $nsp3_{1350–1364}$ was solved at 2.2 Å resolution in space group P 2_1 2_1 2_1 with two copies in the asymmetric unit (Figure 3E).

HLA-DR1 bound $nsp3_{1350–1364}$ via its predicted register (KSAFYILPSIISNEK). Placing P1-Phe and P4-Leu (most enriched in EL and BA motifs) and P6-Ser (enriched at P6) meant that $nsp3_{1350–1364}$ satisfied 3/4 positions within the HLA-DR1 binding motif. Incorporation of P9-Ser is deemed detrimental to HLA-DR1 binding. Thus, P9-Ser is likely the only shortcoming to $nsp3_{1350–1364}$ affinity when considering the core binding motif. Yet, $nsp3_{1350–1364}$ was still one of the highest-binding peptides, suggesting P9 may not be as important to HLA-DR1 binding affinity compared with other pockets (such as P1). In TCR-facing positions, $nsp3_{1350–1364}$ placed the large bulky Tyr residue at position P2 (P2-Tyr), forming a pi-pi stack between aromatic rings of P2-Tyr and His50β, resulting in a protruding surface at this TCR-facing position.

$nsp14_{6420–6434}$: LDAYNMMISAGFSLW

$nsp14_{6420–6434}$ was identified as an HLA-DR1 peptide epitope in HLA-DR1$^+$ transgenic mice and HLA-DR1$^+$ exposed individuals (16/30; 53%) and individuals with no known exposure (3/10; 30%).[18] Exhibiting strong binding to HLA-DR1 (IC_{50} = 541 nM; Figure 1B), the structure of $nsp14_{6420–6434}$ was solved at 1.6 Å resolution in space group P 6_5 2 2 with a single copy in the asymmetric unit (Figure 3F).

HLA-DR1 bound $nsp14_{6420–6434}$ via its predicted register (LDAYNMMISAGFSLW). Once again, $nsp14_{6420–6434}$ satisfied 3/4 of the allowed HLA-DR1 pocket anchors: P1-Tyr, P4-Met,

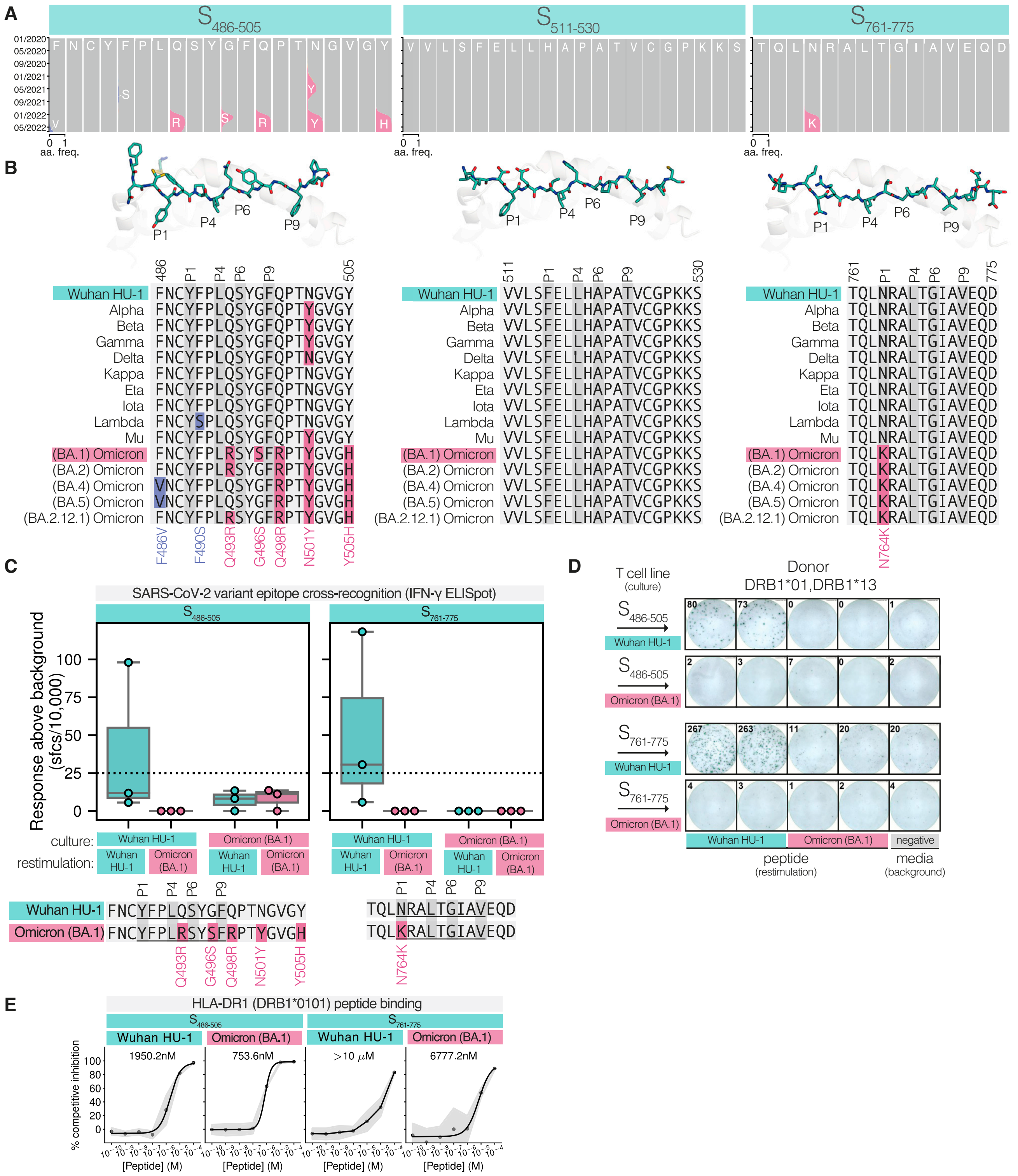

Figure 4. Impact of SARS-CoV-2 variants on epitope HLA-DR1 binding and immunogenicity

(A) Cumulative genotypic frequency plots of amino acid usage of peptide epitope residue positions over time (January 2020–June 2022) in global viral genome sequences (GISAID)[55]: HLA-DR1-$S_{486–505}$ (left), -$S_{511–530}$ (middle), and -$S_{761–775}$ (right). Reference (Wuhan HU-1 strain) amino acid usage frequency (aa. freq.) is colored gray, and mutations associated with Omicron (BA.1) mutations are colored pink. Other mutations present in other lineages are colored blue. Plots were generated using the Nextstrain ncov portal.[58]

(legend continued on next page)

and P6-Ser, while P9-Phe represented a deviation. The incorporation of P9-Phe was reminiscent of the similar $S_{486-505}$ (also P9-Phe), which bound HLA-DR1 at weak affinity. TCR-facing residues were highly aliphatic ($P^{-}1$-Ala, P3-Met, P5-Ile, P7-Ala, P8-Gly, and P11-Leu), the exception being the polar P2-Asn.

In summary, each peptide bound HLA-DR1 canonically in the "forward" orientation that is described for HLA-DR,[48] excluding CLIP.[49] We observed little deviation in the overall structure of HLA-DR1 irrespective of bound peptide (Figure S3A), particularly within the peptide binding groove (Figure S3B), where the solved diversity of bound peptides had little impact on the position of the HLA-DR1 residues that lined each pocket (Figure S3C). NetMHCIIpan-4.1 correctly predicted five of six peptide registers, the exception being $S_{486-505}$ (prediction, FNCYFPLQSYGFQPTNGVGY, core reliability score 89% vs. structure, FNCYFPLQSYGFQPTNGVGY). The bound peptides encompassed a range of affinities (IC_{50} = 7.9 to >10,000 nM), and no peptide fully satisfied the EL or BA motif. HLA-DR1-$S_{511-530}$ presented a "best fit," deviating only by Thr incorporation at P9. Despite this, $S_{511-530}$ was in fact a weak binding peptide *in vitro*. The strongest binding peptide, $M_{176-190}$, also satisfied 3/4 binding pocket preferences: incorporating its mismatch at position P4 (Gly). In fact, satisfying 3/4 EL binding pocket preferences was a commonality to all six complexes. The weakest affinity, $S_{761-775}$ (IC_{50} > 10,000 nM), was the only complex where the P1 position did not incorporate a favored aromatic side chain, which may point toward an increased importance of peptide-HLA-DR1 affinity compared with other pockets. Nevertheless, the degree of adherence to core binding motifs did not clearly distinguish strong from weak binders, suggesting more subtle parameters may determine binding affinity such as the influence of PFRs.[50]

Spike-derived SARS-CoV-2 HLA-DR1 epitopes have mutated in viral variants

The impact of emerging viral mutations of SARS-CoV-2 on immunity has mainly been assessed in the context of escape from Spike-specific neutralizing antibodies.[51–54] However, recent data have demonstrated the loss of T cell responses as crucial to a decline in immunity from reinfection.[11] This has not been explored mechanistically, particularly for HLA-II-restricted responses, which may be critical for prolonged serological protection.

The defined HLA-DR1 epitopes we initially studied used the Wuhan HU-1 sequences (Figure 1A and Table 1). Each of the non-Spike epitopes studied structurally remained stable and largely unmutated (to June 2022) when comparing sequenced genomes within the all-time global GISAID ncov genomic sequencing database (Figure S4A).[55] This included sequences of WHO-designated variants of interest (VOI)/variants of concern (VOC) strains, i.e., Alpha to Omicron. $nsp14_{6420-6434}$ exhibited a brief sequence deviation through incorporation of L6433F (maximal genotypic frequency ~5%; circa November 2021) present in a Delta (21J) strain predominating in Australasian viral genome sequences that did not persist regionally or globally (Figures S4B–S4D).

Within Spike, $S_{511-530}$ also remained stable; however, $S_{486-505}$ and $S_{761-775}$ contained mutations that have persisted in sequenced viral genomes (Figure 4A). Within $S_{486-505}$, N501Y was one of the first defining mutations to emerge[56] circa November 2020 (Figure 4A). N501Y was present within Alpha, Beta, and Gamma, but was absent in Kappa, Eta, Iota, and Lambda before returning in Mu and all Omicron-designated variants (Figure 4B). Later mutations arose centrally to the epitope through the outgrowth of Omicron-designated strains circa November 2021. Within Omicron (BA.1), the earliest Omicron lineage,[57] Q493R and G496S mutations occupied core residue positions P5 and P8 within the HLA-DR1 epitope. The more C-terminal Q498R (P10), N501Y (P13), and Y505H (P17) have persisted in more recent Omicron lineages (BA.2, BA.4, BA.5, and BA.2.12.1), while Q493R has reverted (Q493; BA.4 and BA.5) along with G496S (G496; BA.2, BA.4, BA.5, and BA.2.12.1). Likewise, the spread of Omicron (BA.4 and BA.5) circa May 2022 resulted in the rising frequency of a further mutation, F486V, positioned at $P^{-}3$ in the HLA-DR1 presented epitope.

$S_{761-775}$ was more stable in comparison, but exhibited a single mutation, N764K, located at the P1 anchor residue position with HLA-DR1-$S_{761-775}$. N764K emerged circa November 2021 as a defining mutation of the Omicron (BA.1) variant (Figures 4A and 4B). N764K persisted throughout all Omicron-designated lineages (BA.1, BA.2, BA.4, BA.5, and BA.2.12.1) to become the dominant genotype (~100% genotypic frequency) throughout global SARS-CoV-2 genomic sequencing data circa May 2022. Thus, the N764K mutation within $S_{761-775}$ can be considered a pan-Omicron-lineage mutation.

Omicron (BA.1) mutations result in a loss of recognition of two structurally defined HLA-DR1 epitopes

As Omicron (BA.1) represented the biggest step change in viral sequence to emerge, we synthesized and assessed the impact of Omicron (BA.1) mutations on the immunogenicity of $S_{486-505}$ and $S_{761-775}$; henceforth $S_{486-505}^{Wuhan\ HU-1}$ and $S_{761-775}^{Wuhan\ HU-1}$ or $S_{486-505}^{Omicron\ (BA.1)}$ and $S_{761-775}^{Omicron\ (BA.1)}$. We have recently shown that $CD4^{+}$ T cell clones reactive to $S_{486-505}^{Wuhan\ HU-1}$ presented by HLA-DR1 were not able to cross-recognize $S_{486-505}^{Omicron\ (BA.1)}$ at a clonal level, even at high concentrations

(B) Sequence alignment of epitope sequences in SARS-CoV-2 variants. Variant mutations are as defined by the CoVariants project,[59] highlighting mutations found in Omicron (BA.1) (pink) and other variants (blue) compared with Wuhan HU-1. Anchor residue positions are highlighted (darker gray) based on structural definitions.

(C) *In vitro* immunogenicity of Wuhan HU-1 and Omicron (BA.1) variants of $S_{486-505}$ and $S_{761-775}$: overnight IFN-γ ELISpot (n = 2 wells) in response to short-term culture with both variant peptides in HLA-DR1^{+} donors (n = 3). Data are presented as boxplots (center line, mean; box edges, IQR; whiskers, ±1.5*IQR) with individual response by each donor shown via circles. Data are colored by restimulating peptide during ELISpot assay as indicated below.

(D) Representative IFN-γ ELISpot images of data described in (C) for donor DRB1*01, DRB1*13. Peptides used in the cultured T cell line are across rows, and the variant peptides used for restimulation (overnight ELISpot assay) are indicated in columns. ELISpot images from all three donors are shown in Figure S4E.

(E) Peptide-HLA-DR1 binding curves for Omicron (BA.1) variant peptide epitopes of $S_{486-505}$ and $S_{761-775}$. Data are presented as described in Figure 1B with calculated affinity (IC_{50}) indicated in the insets, representative of n = 3 independent assays, each with n = 3 technical replicates.

of peptide (10 μM).[36] To further this analysis of $S_{486-505}$ and to understand $S_{761-775}$ in Omicron, we assayed Omicron (BA.1) recognition of $S_{486-505}$ and $S_{761-775}$ *in vitro* in our HLA-DR1-positive donors who initially responded to Wuhan HU-1-derived sequences. We assessed cross-recognition between the corresponding Wuhan HU-1 and Omicron (BA.1) peptides by generating short-term cultures (12 days) of T cells exposed independently to each peptide. The expanded T cells were subsequently assayed for responding cells via overnight stimulation with Omicron (BA.1) variant or Wuhan HU-1 peptides, measuring activation via IFN-γ ELISpot. This approach allowed for the assessment of cross-recognition by expanded T cells that may be at low frequency *ex vivo*.

We first retested the expansion of T cells stimulated with Wuhan HU-1 peptides, where one of three donors responded to $S_{486-505}^{\text{Wuhan HU-1}}$ and two of three donors responded to $S_{761-775}^{\text{Wuhan HU-1}}$ (Figures 4C, 4D, and S3E). These same T cell lines exhibited no reactivity to the variant peptides, $S_{486-505}^{\text{Omicron (BA.1)}}$ and $S_{761-775}^{\text{Omicron (BA.1)}}$. We also tested the ability of the same donors to expand T cells against the Omicron (BA.1) variant peptides $S_{486-505}^{\text{Omicron (BA.1)}}$ and $S_{761-775}^{\text{Omicron (BA.1)}}$. In these T cell lines, we observed no reactivity to either the Omicron (BA.1) or the Wuhan HU-1 peptides in any donors. These results indicate that Wuhan HU-1-reactive T cells against the two epitopes do not cross-recognize their mutated counterparts.

To attempt to gain insight into the mechanism surrounding complete loss of T cell activation, we assessed the ability of HLA-DR1 to bind $S_{486-505}$ and $S_{761-775}$ in Omicron (BA.1) form. Both $S_{486-505}^{\text{Omicron (BA.1)}}$ and $S_{761-775}^{\text{Omicron (BA.1)}}$ variant sequences demonstrated an improved ability to bind HLA-DR1: $S_{486-505}^{\text{Wuhan HU-1}}$ IC_{50} = 1,950.2 nM vs. $S_{486-505}^{\text{Omicron (BA.1)}}$ IC_{50} = 753.6 nM and $S_{761-775}^{\text{Wuhan HU-1}}$ IC_{50} > 10 μM vs. $S_{761-775}^{\text{Omicron (BA.1)}}$ IC_{50} = 6,777.2 nM (Figure 4E). Thus, Omicron (BA.1) mutations did not escape the $CD4^{+}$ T cell response through inability to present peptide to T cells on HLA-DR1; in fact, the converse was found, with improved binding.

Viral variant mutations directly alter TCR-facing residues

Given that $S_{486-505}^{\text{Omicron (BA.1)}}$ and $S_{761-775}^{\text{Omicron (BA.1)}}$ exhibited increased HLA-DR1 binding but lost T cell recognition, we sought to generate structures of these peptides bound to HLA-DR1 to understand this mechanistically.

$S_{486-505}^{\text{Omicron (BA.1)}}$ possesses mutations located within both the epitope core and the PFR as determined by the HLA-DR1-$S_{486-505}^{\text{Wuhan HU-1}}$ structure. The structure of HLA-DR1-$S_{486-505}^{\text{Omicron (BA.1)}}$ was solved at 1.6 Å and compared with the similar-resolution Wuhan HU-1 structure (Figure 5A). HLA-DR1 bound $S_{486-505}^{\text{Omicron (BA.1)}}$ using the same register. All core-positioned mutations were in potential upward-facing TCR contact positions, i.e., non-anchor residues: Q493R (P5) and G496S (P8). The overall peptide conformation remained highly similar to that of $S_{486-505}^{\text{Wuhan HU-1}}$, with the most drastic alteration induced by the Q493R mutation, which placed the differing elongated positive charge of P5-Arg in the center of the peptide core. The more subtle G496S resulting in P8-Ser did not alter backbone dihedrals, but placed an additional polar hydroxyl at this position. Thus, loss of cross-recognition between Wuhan HU-1 and the Omicron (BA.1) peptide variant of $S_{486-505}$ was concurrent with mutations to epitope positions typically recognized by TCRs.

A single viral variant mutation can induce a complete register shift in HLA-DR1 presentation

$S_{761-775}^{\text{Omicron (BA.1)}}$ contains a single mutation positioned at the archetypal P1 anchor position for HLA-DR1. This was particularly puzzling for the variant $S_{761-775}^{\text{Omicron (BA.1)}}$ peptide, which possessed only a single Leu → Lys change at position P1 defined by the structure of HLA-DR1-$S_{761-775}^{\text{Wuhan HU-1}}$ shown in Figure 3. This mutation, N764K, in fact increased HLA-DR1 binding affinity (Figure 4E), ruling out loss of HLA binding as an explanation for the loss of T cell recognition. The structure of HLA-DR1-$S_{761-775}^{\text{Omicron (BA.1)}}$ was solved at 2.5 Å resolution in space group C 2 2 2_1 with three copies in the asymmetric unit allowing comparison to HLA-DR1-$S_{761-775}^{\text{Wuhan HU-1}}$ at similar resolutions (Figure 5B). Surprisingly, HLA-DR1-$S_{761-775}^{\text{Omicron (BA.1)}}$ exhibited two distinct peptide conformations within the three copies in the asymmetric unit whereby two copies (copies 1 and 3) exhibited a distinct peptide binding register compared with copy 2. These differential registers were supported by omit map analysis (Figure S5), which evidenced that HLA-DR1 can bind $S_{761-775}^{\text{Omicron (BA.1)}}$ by accommodating the N764K mutation into the P1 pocket and thus maintain the same binding register as $S_{761-775}^{\text{Wuhan HU-1}}$ (copy 2) but also incorporate the neighboring Leu into P1 (copies 1 and 3) to create a +1 register frameshift, i.e., TQLKRALTGIAVEQD to TQLKRALTGIAVEQD. This new register was accommodated by incorporating P1-Leu, P4-Ala, and P9-Ala, while placing the unfavored Thr at P6. The HLA-DR1 P1 binding pocket was able to accommodate the P1-Asn ($S_{761-775}^{\text{Wuhan HU-1}}$), P1-Leu, or P1-Lys ($S_{761-775}^{\text{Omicron (BA.1)}}$) within its deep hydrophobic pocket volume (Figure 5C). In each case, the residues surrounding the P1 pocket were positionally identical irrespective of buried cargo (Figure 5D), highlighting an extensive permissibility of the HLA-DR1 P1 pocket to accommodate diverse amino acids, including residues absent from the EL and BA motifs. While the +1 frameshifted register of the peptide was present in 2/3 of the copies within the asymmetric unit of the crystal structure, the relative distribution between the two registers in solution is unclear. Despite the results from these structural variants, the complete abrogation of T cell reactivity argues that, under physiological conditions, the shifted +1 register binding is favored. As a result, the frameshifted peptide epitope exhibited a drastically altered peptide landscape to the TCR repertoire compared with HLA-DR1-$S_{761-775}^{\text{Wuhan HU-1}}$, P3, Ala → Arg; P5, Thr → Leu; and P7, Ile → Gly, thus explaining the lack of immune cross-recognition against the Omicron (BA.1) variant of $S_{761-775}$ peptide in Wuhan HU-1 Spike-experienced HLA-DR1-positive donors.

DISCUSSION

Our analyses of SARS-CoV-2 $CD4^{+}$ T cell epitopes has provided insights into viral antigen recognition from immunodominance down to the molecular level via structural characterization of ancestral- and variant-derived viral epitopes presented by HLA-DR1. These first provide a resource of model-characterized

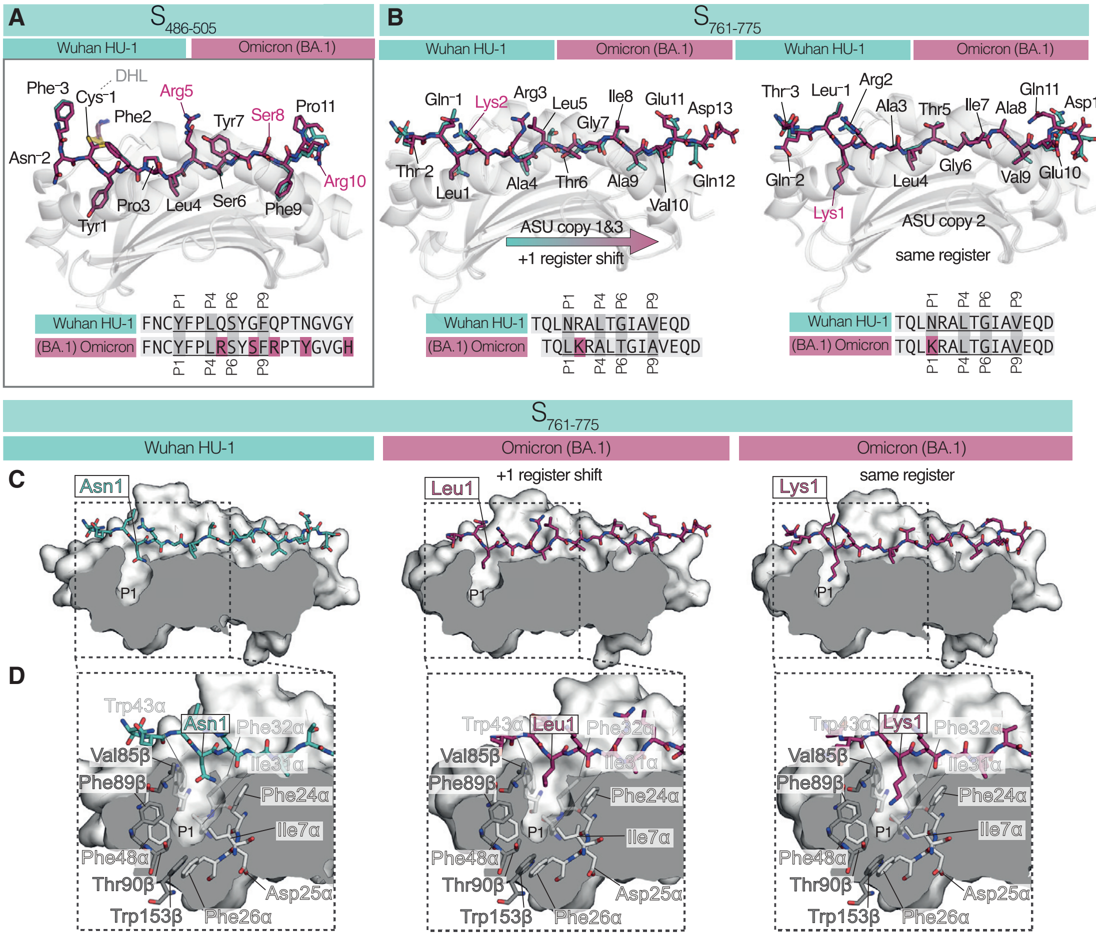

Figure 5. Structural implications of Omicron (BA.1) mutations on HLA-DR1 epitope presentation

(A) Structural overview of HLA-DR1-$S_{486–505}^{Omicron\ (BA.1)}$ aligned and overlaid on top of the HLA-DR1-$S_{486–505}^{Wuhan\ HU-1}$ structure. For both, the HLA-DR1 peptide binding groove is shown as a gray cartoon representation, with the $S_{486–505}^{Omicron\ (BA.1)}$ peptide shown as sticks (C atoms, pink) and $S_{486–505}^{Wuhan\ HU-1}$ peptide shown as sticks (C atoms, aqua). Amino acid mutations contained within $S_{486–505}^{Omicron\ (BA.1)}$ that differ between variants are highlighted by the pink residue labels (inset).

(B) Structural overview of HLA-DR1-$S_{761–775}^{Omicron\ (BA.1)}$ aligned and overlaid on top of the HLA-DR1-$S_{761–775}^{Wuhan\ HU-1}$ structure in two registers: (left) a +1 register shift relative to HLA-DR1-$S_{761–775}^{Wuhan\ HU-1}$ observed in asymmetric unit (ASU) copies 1and3 and (right) the same register as HLA-DR1-$S_{761–775}^{Wuhan\ HU-1}$ observed in ASU copy 2. Colored and represented as described in (A).

(C) Surface cross-sectional view of the HLA-DR1 binding groove of HLA-DR1-$S_{761–775}^{Wuhan\ HU-1}$ (left), HLA-DR1-$S_{761–775}^{Omicron\ (BA.1)}$ +1 register shift (middle), and HLA-DR1-$S_{761–775}^{Omicron\ (BA.1)}$ same register (right). In each, the HLA-DR1 binding groove has been clipped in the z plane at approximately the deepest point in the P1 pocket. The residue buried in the deep hydrophobic P1 pocket is labeled with peptide represented as sticks (colored as previously).

(D) Expanded cross-sectional view of the P1 pocket of $S_{761–775}$ peptides/conformations. In each, residues that line the P1 pocket are shown as stick representations (DRA, light gray C atoms; DR1β, dark gray C atoms). Residues that form the back of the pocket (as viewed) are shown semi-transparent. Asn82β, which forms the front of the pocket (as viewed), is omitted for clarity.

HLA-II epitopes for tracking $CD4^+$ T cells in COVID-19. Further, these data provide a base on which we could understand mechanistically the impact SARS-CoV-2 variants had on immune recognition of $CD4^+$ T cells. The key outcome of this was that viral variants of two HLA-II-presented epitopes escaped pre-existing immune memory in two ways. An epitope from a highly variable region of Spike induced loss of $CD4^+$ T cell memory via multiple mutations that are likely directly recognized by TCRs. A second more stable epitope contained a single amino acid mutation found in all Omicron lineages to date. Located at a key anchor position, which dictates peptide selection by HLA-DR1, this mutation did not lessen or ablate epitope presentation but instead induced a drastic alteration in its presentation by causing a register shift within the HLA-DR groove. The occurrence of register shift-inducing mutations may have wider implications in HLA-II-presented epitopes in the context of immune memory in general.

The most immunogenic HLA-II peptides are thought to have a predicted binding affinity of K_D <1,000 nM.[60] All three Spike epitopes studied structurally exhibit *predicted* affinities for HLA-DR1 within this proposed threshold, yet we demonstrated that many showed experimentally weaker IC_{50} binding. These weaker-binding peptides could bind into the HLA-DR groove

and elicit memory responses in individuals who were antigen experienced, which can be detected *in vitro* (this study) and *ex vivo*.[36] Weaker-binding peptides may be compensated for by antigen abundance to reach sufficient antigen density.[61] Indeed, SARS-CoV-2 Nucleocapsid, Spike, and Membrane proteins are the most abundant in infected cells at the protein[62] and transcript[63] levels. Together, these results highlight the difficulty in accurately predicting important and immunodominant HLA-II epitopes through *in silico* assessment alone of peptide-HLA-II binding.

Of the three Spike epitopes studied structurally, $S_{486–505}$ was most frequently targeted by mutation. $S_{486–505}$ is located within the receptor binding motif (RBM),[64–66] which is highly mutable through positive selection at the Spike-ACE2 interface[67] and the immunodominance of RBM-binding neutralizing antibodies.[68] Mutations that enhance Spike-ACE2 binding are associated with increased replication efficiency[69] and transmissibility.[70] Indeed, Q493R, G496S, Q498R, and N501Y of Omicron (BA.1), present in $S_{486–505}$, form new high-enthalpy receptor-ligand interactions.[71] In contrast, Y505H is detrimental to Spike-ACE2 contacts[72] and free energy estimations.[71] Omicron strain Spike-ACE2 affinity is not significantly increased from the earlier-lineage Delta, which is thus suggestive that Omicron-lineage mutations may be driven by another factor,[72] such as immune escape. Indeed, Omicron broadly escapes Spike-specific neutralizing antibodies,[51–54] including N501Y.[73] Viral accumulation of Q493R, which is an escape mutant of drug/antibody binding,[74] and subsequent drug resistance were demonstrated in an individual treated with mAb cocktails of bamlanivimab and etesevimab.[75] It is difficult to deduce whether these Omicron Spike mutations were driven by T cell escape, antibody escape, or viral-intrinsic evolution of fitness. Nevertheless, we have shown that mutations in $S_{486–505}$ enable escape of epitope-specific $CD4^+$ T cell immunity through alterations in TCR-facing residues that are sufficient to ablate T cell recognition in HLA-$DR1^+$ donors.

$S_{761–775}$ differs by being located within the S2 domain of Spike, which is more conserved across coronaviruses.[76] $S_{761–775}$ does not directly bind ACE2 and is not likely to be accessible to B cells,[77,78] as it is within a tightly packed helix sandwiched between the central helical domain and the N-terminal domain of the prefusion Spike trimer.[79] $S_{761–775}$ exhibited a single mutation, N764K, which is persistent in all Omicron strains, having reached and maintained >98% genotypic frequency since around April 2022.[55,58] The N764K mutation affected the archetypal P1 pocket of the $S_{761–775}$ epitope presented by HLA-DR1 through its positioning, inducing a frameshift concurrent with loss of cross-recognition. Such frameshift mutations have been postulated previously in a DRB1*07:01 epitope,[19] but we herein demonstrate structural evidence of this mechanism of $CD4^+$ T cell escape.

While we demonstrate two examples of $CD4^+$ T cell epitope escape, T cell immunity toward SARS-CoV-2 viral variants is more robust compared with antibody immunity,[80] potentially due to the sheer breadth of epitopes recognized by T cells.[81] Characterization of the TCR clonotypes and the modes of their binding would aid the mechanistic explanations as to why, in the case of $S_{486–505}$ and $S_{761–775}$, TCRs of HLA-DR-restricted $CD4^+$ T cells do not exhibit the capacity to cross-recognize their mutated variant counterparts. Nevertheless, these data highlight the importance of vaccinating against circulating Spike variants, i.e., Spike Omicron (BA.1) mRNA-1273.214[82] and Omicron (BA.4/BA.5) BNT162b2,[83] and/or targeting more conserved regions of coronaviral proteomes, which exhibit higher homology and also induce strong humoral and T cell responses.[84]

Our structural studies show how mutations to virus sequences may induce drastic alterations in HLA-II-presented peptides through either multiple epitope changes or seemingly subtle mutations. The net result of both observed mechanisms was the same (i.e., a loss of cross-recognition/memory), yet while the former may have been easily predicted due to the large number of mutations, the latter would not have been obvious without experimental characterization. The implication of this is that we require further ways to identify and predict the occurrence of register-shifting mutations and the frequency at which they influence $CD4^+$ T cell recognition/memory such that future vaccines are appropriately designed to induce cross-protection against heterologous viruses.

Limitations of the study

Our study set out during the pandemic to use the HLA-DR1 allotype as a model to assess the presentation and recognition of SARS-CoV-2 by $CD4^+$ T cells. Emerging epitopes were selected (circa November 2020) from published studies that performed thorough and extensive epitope mapping throughout the SARS-CoV-2 genome. By selecting peptides from these published data, we have not characterized the full breadth of potential HLA-DR1 epitopes. As the peptides were selected from a combination of studies describing immunogenicity in unexposed individuals[14] and individuals who were SARS-CoV-2 experienced,[15,16] we have not drawn conclusions from the collective responses to individual peptides observed in our donors to inform whether donors had (1) experienced a previous infection, (2) responded to vaccination, or (3) immune protection against viral infection/reinfection. This is in part because we did not screen peptide responses against the full breadth of epitopes, but also because some peptides were immunogenic in individuals who were unexposed (pre-/early pandemic), and thus may represent cross-reactive memory to antigens other than SARS-CoV-2. Similarly, the effect of variant mutations on the breadth of HLA-DR1 epitopes was not studied, as we could focus our in-depth structural analysis on only a small subset of peptides. Thus, the overall rate of epitope loss within host-viral evolution and whether other mechanisms of escape also exist cannot be determined. Nevertheless, our study highlights the consequences that viral evolution could have generally on the peptide epitopes presented to $CD4^+$ T cells on the HLA-II platform and that could have relevance in all host-viral interactions.

STAR★METHODS

Detailed methods are provided in the online version of this paper and include the following:

- KEY RESOURCES TABLE
- RESOURCE AVAILABILITY
  - ○ Lead contact
  - ○ Materials availability
  - ○ Data and code availability
- EXPERIMENTAL MODEL AND STUDY PARTICIPANT DETAILS

- Ethical approval, participant recruitment & consenting
- Generation of cultured T cell lines from donor PBMCs
- Cell lines
- METHOD DETAILS
  - Production of peptide-HLA-DR1 proteins
  - HLA-II peptide binding assays
  - Peptide immunogenicity analysis
  - Crystallization and structure determination of peptide-HLA-DR1 complexes
  - SARS-CoV-2 mutational sequence analysis
- QUANTIFICATION AND STATISTICAL ANALYSIS
  - HLA-II peptide binding assays
  - ELISpot analyses

SUPPLEMENTAL INFORMATION

Supplemental information can be found online at https://doi.org/10.1016/j.celrep.2023.112827.

ACKNOWLEDGMENTS

This work was funded by a Sêr Cymru–Tackling COVID-19 Award from the Welsh Government/Llywodraeth Cymru to B.J.M. and A.G. The authors would like to thank the blood donors for their contribution to this work. The authors would like to thank Diamond Light Source for beamtime (proposals mx20147, mx29502, and mx29990) and the staff of beamlines i04 and i04-1 for assistance with data collection. B.J.M. is a recipient of a Health and Care Research Wales–Health Research Fellowship (HF-21-1886). A.G. and A.M.G. are supported by a Wellcome Trust Collaborative Award in Science (209213/Z/17/Z). H.M.L. was supported by the UK Coronavirus Immunology Consortium (UK-CIC) funded by NIHR/MRC MR/V028448/1.

AUTHOR CONTRIBUTIONS

Conceptualization, Y.C., A.M.G., B.J.M., and A.G.; data curation, Y.C., P.R., and B.J.M.; formal analysis, Y.C., P.R., B.J.M., and A.G.; funding acquisition, B.J.M. and A.G.; investigation, Y.C., G.H.M., D.O.S., T.A.H., H.M.L., A.M.G., and B.J.M.; methodology, Y.C., G.H.M., D.O.S., A.G.-W., T.A.H., H.M.L., A.M.G., P.R., B.J.M., and A.G.; project administration, A.M.G., P.R., B.J.M., and A.G.; resources, A.K.S., A.M.G., P.R., B.J.M., and A.G.; supervision, A.M.G., B.J.M., and A.G.; validation, Y.C., P.R., B.J.M., and A.G.; visualization, Y.C., B.J.M., and A.G.; writing – original draft, B.J.M. and A.G.; writing – review & editing, Y.C., G.H.M., D.O.S., A.G.-W., A.K.S., H.M.L., A.M.G., P.R., B.J.M., and A.G.

DECLARATION OF INTERESTS

The authors declare no competing interests.

INCLUSION AND DIVERSITY

One or more of the authors of this paper self-identifies as an underrepresented ethnic minority in their field of research or within their geographical location. We support inclusive, diverse, and equitable conduct of research.

Received: March 8, 2023
Revised: May 21, 2023
Accepted: June 30, 2023
Published: July 19, 2023

REFERENCES

1. Rydyznski Moderbacher, C., Ramirez, S.I., Dan, J.M., Grifoni, A., Hastie, K.M., Weiskopf, D., Belanger, S., Abbott, R.K., Kim, C., Choi, J., et al. (2020). Antigen-specific adaptive immunity to SARS-CoV-2 in acute COVID-19 and associations with age and disease severity. Cell *183*, 996–1012.e19. https://doi.org/10.1016/J.CELL.2020.09.038.

2. Robbiani, D.F., Gaebler, C., Muecksch, F., Lorenzi, J.C.C., Wang, Z., Cho, A., Agudelo, M., Barnes, C.O., Gazumyan, A., Finkin, S., et al. (2020). Convergent antibody responses to SARS-CoV-2 in convalescent individuals. Nature *584*, 437–442. https://doi.org/10.1038/s41586-020-2456-9.

3. Anderson, E.J., Rouphael, N.G., Widge, A.T., Jackson, L.A., Roberts, P.C., Makhene, M., Chappell, J.D., Denison, M.R., Stevens, L.J., Pruijssers, A.J., et al. (2020). Safety and immunogenicity of SARS-CoV-2 mRNA-1273 vaccine in older adults. N. Engl. J. Med. *383*, 2427–2438. https://doi.org/10.1056/NEJMOA2028436.

4. Folegatti, P.M., Ewer, K.J., Aley, P.K., Angus, B., Becker, S., Belij-Rammerstorfer, S., Bellamy, D., Bibi, S., Bittaye, M., Clutterbuck, E.A., et al. (2020). Safety and immunogenicity of the ChAdOx1 nCoV-19 vaccine against SARS-CoV-2: a preliminary report of a phase 1/2, single-blind, randomised controlled trial. Lancet *396*, 467–478. https://doi.org/10.1016/S0140-6736(20)31604-4.

5. Sahin, U., Muik, A., Vogler, I., Derhovanessian, E., Kranz, L.M., Vormehr, M., Quandt, J., Bidmon, N., Ulges, A., Baum, A., et al. (2021). BNT162b2 vaccine induces neutralizing antibodies and poly-specific T cells in humans. Nature *595*, 572–577. https://doi.org/10.1038/s41586-021-03653-6.

6. Stephenson, K.E., Le Gars, M., Sadoff, J., de Groot, A.M., Heerwegh, D., Truyers, C., Atyeo, C., Loos, C., Chandrashekar, A., McMahan, K., et al. (2021). Immunogenicity of the Ad26.COV2.S Vaccine for COVID-19. JAMA *325*, 1535–1544. https://doi.org/10.1001/JAMA.2021.3645.

7. Mathew, D., Giles, J.R., Baxter, A.E., Oldridge, D.A., Greenplate, A.R., Wu, J.E., Alanio, C., Kuri-Cervantes, L., Pampena, M.B., D'Andrea, K., et al. (2020). Deep immune profiling of COVID-19 patients reveals distinct immunotypes with therapeutic implications. Science *369*, eabc8511. https://doi.org/10.1126/SCIENCE.ABC8511.

8. Bergamaschi, L., Mescia, F., Turner, L., Hanson, A.L., Kotagiri, P., Dunmore, B.J., Ruffieux, H., De Sa, A., Huhn, O., Morgan, M.D., et al. (2021). Longitudinal analysis reveals that delayed bystander CD8+ T cell activation and early immune pathology distinguish severe COVID-19 from mild disease. Immunity *54*, 1257–1275.e8. https://doi.org/10.1016/J.IMMUNI.2021.05.010.

9. Le Bert, N., Tan, A.T., Kunasegaran, K., Tham, C.Y.L., Hafezi, M., Chia, A., Chng, M.H.Y., Lin, M., Tan, N., Linster, M., et al. (2020). SARS-CoV-2-specific T cell immunity in cases of COVID-19 and SARS, and uninfected controls. Nature *584*, 457–462. https://doi.org/10.1038/s41586-020-2550-z.

10. Cohen, K.W., Linderman, S.L., Moodie, Z., Czartoski, J., Lai, L., Mantus, G., Norwood, C., Nyhoff, L.E., Edara, V.V., Floyd, K., et al. (2021). Longitudinal analysis shows durable and broad immune memory after SARS-CoV-2 infection with persisting antibody responses and memory B and T cells. Cell Rep. Med. *2*, 100354. https://doi.org/10.1016/J.XCRM.2021.100354.

11. Scurr, M.J., Lippiatt, G., Capitani, L., Bentley, K., Lauder, S.N., Smart, K., Somerville, M.S., Rees, T., Stanton, R.J., Gallimore, A., et al. (2022). Magnitude of venous or capillary blood-derived SARS-CoV-2-specific T cell response determines COVID-19 immunity. Nat. Commun. *13*, 5422. https://doi.org/10.1038/s41467-022-32985-8.

12. Grifoni, A., Sidney, J., Zhang, Y., Scheuermann, R.H., Peters, B., and Sette, A. (2020). A sequence homology and bioinformatic approach can predict candidate targets for immune responses to SARS-CoV-2. Cell Host Microbe *27*, 671–680.e2. https://doi.org/10.1016/J.CHOM.2020.03.002.

13. Kiyotani, K., Toyoshima, Y., Nemoto, K., and Nakamura, Y. (2020). Bioinformatic prediction of potential T cell epitopes for SARS-Cov-2. J. Hum. Genet. *65*, 569–575. https://doi.org/10.1038/s10038-020-0771-5.

14. Mateus, J., Grifoni, A., Tarke, A., Sidney, J., Ramirez, S.I., Dan, J.M., Burger, Z.C., Rawlings, S.A., Smith, D.M., Phillips, E., et al. (2020). Selective and cross-reactive SARS-CoV-2 T cell epitopes in unexposed humans. Science *370*, 89–94. https://doi.org/10.1126/SCIENCE.ABD3871.

15. Peng, Y., Mentzer, A.J., Liu, G., Yao, X., Yin, Z., Dong, D., Dejnirattisai, W., Rostron, T., Supasa, P., Liu, C., et al. (2020). Broad and strong memory CD4+ and CD8+ T cells induced by SARS-CoV-2 in UK convalescent individuals following COVID-19. Nat. Immunol. *21*, 1336–1345. https://doi.org/10.1038/s41590-020-0782-6.

16. Nelde, A., Bilich, T., Heitmann, J.S., Maringer, Y., Salih, H.R., Roerden, M., Lübke, M., Bauer, J., Rieth, J., Wacker, M., et al. (2021). SARS-CoV-2-derived peptides define heterologous and COVID-19-induced T cell recognition. Nat. Immunol. *22*, 74–85. https://doi.org/10.1038/s41590-020-00808-x.

17. Tarke, A., Sidney, J., Kidd, C.K., Dan, J.M., Ramirez, S.I., Yu, E.D., Mateus, J., da Silva Antunes, R., Moore, E., Rubiro, P., et al. (2021). Comprehensive analysis of T cell immunodominance and immunoprevalence of SARS-CoV-2 epitopes in COVID-19 cases. Cell Rep. Med. *2*, 100204. https://doi.org/10.1016/J.XCRM.2021.100204.

18. Prakash, S., Srivastava, R., Coulon, P.-G., Dhanushkodi, N.R., Chentoufi, A.A., Tifrea, D.F., Edwards, R.A., Figueroa, C.J., Schubl, S.D., Hsieh, L., et al. (2021). Genome-wide B cell, CD4 + , and CD8 + T cell epitopes that are highly conserved between human and animal Coronaviruses, identified from SARS-CoV-2 as targets for preemptive Pan-Coronavirus vaccines. J. Immunol. *206*, 2566–2582. https://doi.org/10.4049/JIMMUNOL.2001438.

19. Obermair, F.J., Renoux, F., Heer, S., Lee, C.H., Cereghetti, N., Loi, M., Maestri, G., Haldner, Y., Wuigk, R., Iosefson, O., et al. (2022). High-resolution profiling of MHC II peptide presentation capacity reveals SARS-CoV-2 CD4 T cell targets and mechanisms of immune escape. Sci. Adv. *8*, 5394. https://doi.org/10.1126/SCIADV.ABL5394.

20. Augusto, D.G., Yusufali, T., Sabatino, J.J., Jr., Peyser, N.D., Murdolo, L.D., Butcher, X., Murray, V., Pae, V., Sarvadhavabhatla, S., Beltran, F., et al. (2022). A common allele of HLA mediates asymptomatic SARS-CoV-2 infection. Preprint at medRxiv. https://doi.org/10.1101/2021.05.13.21257065.

21. Swadling, L., Diniz, M.O., Schmidt, N.M., Amin, O.E., Chandran, A., Shaw, E., Pade, C., Gibbons, J.M., Le Bert, N., Tan, A.T., et al. (2021). Pre-existing polymerase-specific T cells expand in abortive seronegative SARS-CoV-2. Nature *601*, 110–117. https://doi.org/10.1038/s41586-021-04186-8.

22. Greenshields-Watson, A., Attaf, M., MacLachlan, B.J., Whalley, T., Rius, C., Wall, A., Lloyd, A., Hughes, H., Strange, K.E., Mason, G.H., et al. (2020). CD4+ T cells recognize conserved Influenza A epitopes through shared patterns of V-gene usage and complementary biochemical features. Cell Rep. *32*, 107885. https://doi.org/10.1016/J.CELREP.2020.107885.

23. Hennecke, J., Carfi, A., and Wiley, D.C. (2000). Structure of a covalently stabilized complex of a human αβ T-cell receptor, influenza HA peptide and MHC class II molecule, HLA-DR1. EMBO J. *19*, 5611–5624. https://doi.org/10.1093/EMBOJ/19.21.5611.

24. Galperin, M., Farenc, C., Mukhopadhyay, M., Jayasinghe, D., Decroos, A., Benati, D., Tan, L.L., Ciacchi, L., Reid, H.H., Rossjohn, J., et al. (2018). CD4+ T cell–mediated HLA class II cross-restriction in HIV controllers. Sci. Immunol. *3*, eaat0687. https://doi.org/10.1126/SCIIMMUNOL.AAT0687.

25. Chaurasia, P., Nguyen, T.H.O., Rowntree, L.C., Juno, J.A., Wheatley, A.K., Kent, S.J., Kedzierska, K., Rossjohn, J., and Petersen, J. (2021). Structural basis of biased T cell receptor recognition of an immunodominant HLA-A2 epitope of the SARS-CoV-2 spike protein. J. Biol. Chem. *297*, 101065. https://doi.org/10.1016/J.JBC.2021.101065.

26. Dolton, G., Rius, C., Hasan, M.S., Wall, A., Szomolay, B., Behiry, E., Whalley, T., Southgate, J., Fuller, A., et al.; COVID-19 Genomics UK COG-UK consortium (2022). Emergence of immune escape at dominant SARS-CoV-2 killer T cell epitope. Cell *185*, 2936–2951.e19. https://doi.org/10.1016/J.CELL.2022.07.002.

27. Wu, D., Kolesnikov, A., Yin, R., Guest, J.D., Gowthaman, R., Shmelev, A., Serdyuk, Y., Dianov, D.V., Efimov, G.A., Pierce, B.G., et al. (2022). Structural assessment of HLA-A2-restricted SARS-CoV-2 spike epitopes recognized by public and private T-cell receptors. Nat. Commun. *13*, 19. https://doi.org/10.1038/s41467-021-27669-8.

28. Wu, D., Efimov, G.A., Bogolyubova, A.V., Pierce, B.G., and Mariuzza, R.A. (2023). Structural insights into protection against a SARS-CoV-2 spike variant by T cell receptor (TCR) diversity. J. Biol. Chem. *299*, 103035. https://doi.org/10.1016/j.jbc.2023.103035.

29. Shimizu, A., Kawana-Tachikawa, A., Yamagata, A., Han, C., Zhu, D., Sato, Y., Nakamura, H., Koibuchi, T., Carlson, J., Martin, E., et al. (2013). Structure of TCR and antigen complexes at an immunodominant CTL epitope in HIV-1 infection. Sci. Rep. *3*, 1–9. https://doi.org/10.1038/srep03097.

30. Cole, D.K., Fuller, A., Dolton, G., Zervoudi, E., Legut, M., Miles, K., Blanchfield, L., Madura, F., Holland, C.J., Bulek, A.M., et al. (2017). Dual molecular mechanisms govern escape at immunodominant HLA A2-restricted HIV epitope. Front. Immunol. *8*, 1503. https://doi.org/10.3389/fimmu.2017.01503.

31. Lippolis, J.D., White, F.M., Marto, J.A., Luckey, C.J., Bullock, T.N.J., Shabanowitz, J., Hunt, D.F., and Engelhard, V.H. (2002). Analysis of MHC class II antigen processing by quantitation of peptides that constitute nested sets. J. Immunol. *169*, 5089–5097. https://doi.org/10.4049/jimmunol.169.9.5089.

32. Holland, C.J., Cole, D.K., and Godkin, A. (2013). Re-directing CD4(+) T cell responses with the flanking residues of MHC class II-bound peptides: the core is not enough. Front. Immunol. *4*, 172. https://doi.org/10.3389/fimmu.2013.00172.

33. McMichael, A. (1998). T cell responses and viral escape. Cell *93*, 673–676. https://doi.org/10.1016/S0092-8674(00)81428-2.

34. Harcourt, G.C., Garrard, S., Davenport, M.P., Edwards, A., and Phillips, R.E. (1998). HIV-1 variation diminishes CD4 T lymphocyte recognition. J. Exp. Med. *188*, 1785–1793. https://doi.org/10.1084/JEM.188.10.1785.

35. Reynisson, B., Alvarez, B., Paul, S., Peters, B., and Nielsen, M. (2020). NetMHCpan-4.1 and NetMHCIIpan-4.0: improved predictions of MHC antigen presentation by concurrent motif deconvolution and integration of MS MHC eluted ligand data. Nucleic Acids Res. *48*, W449–W454. https://doi.org/10.1093/NAR/GKAA379.

36. Tye, E.X.C., Jinks, E., Haigh, T.A., Kaul, B., Patel, P., Parry, H.M., Newby, M.L., Crispin, M., Kaur, N., Moss, P., et al. (2022). Mutations in SARS-CoV-2 spike protein impair epitope-specific CD4+ T cell recognition. Nat. Immunol. *23*, 1726–1734. https://doi.org/10.1038/s41590-022-01351-7.

37. Godkin, A.J., Davenport, M.P., Willis, A., Jewell, D.P., and Hill, A.V. (1998). Use of complete eluted peptide sequence data from HLA-DR and -DQ molecules to predict T cell epitopes, and the influence of the nonbinding terminal regions of ligands in epitope selection. J. Immunol. *161*, 850–858. https://doi.org/10.4049/jimmunol.161.2.850.

38. Lamb, J.R., Eckels, D.D., Lake, P., Woody, J.N., and Green, N. (1982). Human T-cell clones recognize chemically synthesized peptides of influenza haemagglutinin. Nature *300*, 66–69. https://doi.org/10.1038/300066a0.

39. Hennecke, J., and Wiley, D.C. (2002). Structure of a complex of the human α/β T cell receptor (TCR) HA1.7, Influenza hemagglutinin peptide, and major histocompatibility complex class II molecule, HLA-DR4 (DRA*0101 and DRB1*0401). J. Exp. Med. *195*, 571–581. https://doi.org/10.1084/jem.20011194.

40. Rudolph, M.G., Stanfield, R.L., and Wilson, I. a ((2006). How TCRs bind MHCs, peptides, and coreceptors. Annu. Rev. Immunol. *24*, 419–466. https://doi.org/10.1146/annurev.immunol.23.021704.115658.

41. Rossjohn, J., Gras, S., Miles, J.J., Turner, S.J., Godfrey, D.I., and McCluskey, J. (2015). T cell antigen receptor recognition of antigen-presenting molecules. Annu. Rev. Immunol. *33*, 169–200. https://doi.org/10.1146/annurev-immunol-032414-112334.

42. Low, J.S., Vaqueirinho, D., Mele, F., Foglierini, M., Jerak, J., Perotti, M., Jarrossay, D., Jovic, S., Perez, L., Cacciatore, R., et al. (2021). Clonal analysis of immunodominance and crossreactivity of the CD4 T cell

response to SARS-CoV-2. Science *372*, 1336–1341. https://doi.org/10.1126/SCIENCE.ABG8985.

43. Zhao, J., Wang, L., Schank, M., Dang, X., Lu, Z., Cao, D., Khanal, S., Nguyen, L.N., Nguyen, L.N.T., Zhang, J., et al. (2021). SARS-CoV-2 specific memory T cell epitopes identified in COVID-19-recovered subjects. Virus Res. *304*, 198508. https://doi.org/10.1016/J.VIRUSRES.2021.198508.

44. Verhagen, J., van der Meijden, E.D., Lang, V., Kremer, A.E., Völkl, S., Mackensen, A., Aigner, M., and Kremer, A.N. (2021). Human CD4+ T cells specific for dominant epitopes of SARS-CoV-2 Spike and Nucleocapsid proteins with therapeutic potential. Clin. Exp. Immunol. *205*, 363–378. https://doi.org/10.1111/CEI.13627.

45. Zhang, Y., Yang, Z., Tang, M., Li, H., Tang, T., Li, G., Zhong, Y., Zhang, X., Wang, X., and Wang, C. (2022). Three specific potential epitopes that could be recognized by T cells of convalescent COVID-19 patients were identified from spike protein. Front. Immunol. *13*, 75. https://doi.org/10.3389/FIMMU.2022.752622/BIBTEX.

46. Heide, J., Schulte, S., Kohsar, M., Brehm, T.T., Herrmann, M., Karsten, H., Marget, M., Peine, S., Johansson, A.M., Sette, A., et al. (2021). Broadly directed SARS-CoV-2-specific CD4+ T cell response includes frequently detected peptide specificities within the membrane and nucleoprotein in patients with acute and resolved COVID-19. PLoS Pathog. *17*, e1009842. https://doi.org/10.1371/JOURNAL.PPAT.1009842.

47. Poluektov, Y., George, M., Daftarian, P., and Delcommenne, M.C. (2021). Assessment of SARS-CoV-2 specific CD4(+) and CD8 (+) T cell responses using MHC class I and II tetramers. Vaccine *39*, 2110–2116. https://doi.org/10.1016/J.VACCINE.2021.03.008.

48. Klobuch, S., Lim, J.J., Balen, P. van, Kester, M.G.D., Klerk, W., Arnoud, H.d.R., Pothast, C.R., Jedema, I., Drijfhout, J.W., Rossjohn, J., et al. (2022). Human T cells recognize HLA-DP-bound peptides in two orientations. Proc. Natl. Acad. Sci. USA *119*, 2214331119. https://doi.org/10.1073/PNAS.2214331119.

49. Günther, S., Schlundt, A., Sticht, J., Roske, Y., Heinemann, U., Wiesmüller, K.H., Jung, G., Falk, K., Rötzschke, O., and Freund, C. (2010). Bidirectional binding of invariant chain peptides to an MHC class II molecule. Proc. Natl. Acad. Sci. USA *107*, 22219–22224. https://doi.org/10.1073/pnas.1014708107.

50. MacLachlan, B.J., Dolton, G., Papakyriakou, A., Greenshields-Watson, A., Mason, G.H., Schauenburg, A., Besneux, M., Szomolay, B., Elliott, T., Sewell, A.K., et al. (2019). Human leukocyte antigen (HLA) class II peptide flanking residues tune the immunogenicity of a human tumor-derived epitope. J. Biol. Chem. *294*, 20246–20258. https://doi.org/10.1074/jbc.RA119.009437.

51. Planas, D., Saunders, N., Maes, P., Guivel-Benhassine, F., Planchais, C., Buchrieser, J., Bolland, W.H., Porrot, F., Staropoli, I., Lemoine, F., et al. (2021). Considerable escape of SARS-CoV-2 Omicron to antibody neutralization. Nature *602*, 671–675. https://doi.org/10.1038/s41586-021-04389-z.

52. Cao, Y., Wang, J., Jian, F., Xiao, T., Song, W., Yisimayi, A., Huang, W., Li, Q., Wang, P., An, R., et al. (2021). Omicron escapes the majority of existing SARS-CoV-2 neutralizing antibodies. Nature *602*, 657–663. https://doi.org/10.1038/s41586-021-04385-3.

53. Garcia-Beltran, W.F., Lam, E.C., St Denis, K., Nitido, A.D., Garcia, Z.H., Hauser, B.M., Feldman, J., Pavlovic, M.N., Gregory, D.J., Poznansky, M.C., et al. (2021). Multiple SARS-CoV-2 variants escape neutralization by vaccine-induced humoral immunity. Cell *184*, 2523. https://doi.org/10.1016/J.CELL.2021.04.006.

54. Hachmann, N.P., Miller, J., Collier, A.Y., Ventura, J.D., Yu, J., Rowe, M., Bondzie, E.A., Powers, O., Surve, N., Hall, K., et al. (2022). Neutralization escape by SARS-CoV-2 Omicron subvariants BA.2.12.1, BA.4, and BA.5. N. Engl. J. Med. *387*, 86–88. https://doi.org/10.1056/NEJMC2206576.

55. Elbe, S., and Buckland-Merrett, G. (2017). Data, disease and diplomacy: GISAID's innovative contribution to global health. Glob. Chall. *1*, 33–46. https://doi.org/10.1002/GCH2.1018.

56. Liu, Y., Liu, J., Plante, K.S., Plante, J.A., Xie, X., Zhang, X., Ku, Z., An, Z., Scharton, D., Schindewolf, C., et al. (2021). The N501Y spike substitution enhances SARS-CoV-2 infection and transmission. Nature *602*, 294–299. https://doi.org/10.1038/s41586-021-04245-0.

57. Viana, R., Moyo, S., Amoako, D.G., Tegally, H., Scheepers, C., Althaus, C.L., Anyaneji, U.J., Bester, P.A., Boni, M.F., Chand, M., et al. (2022). Rapid epidemic expansion of the SARS-CoV-2 Omicron variant in Southern Africa. Nature *603*, 679–686. https://doi.org/10.1038/s41586-022-04411-y.

58. Hadfield, J., Megill, C., Bell, S.M., Huddleston, J., Potter, B., Callender, C., Sagulenko, P., Bedford, T., and Neher, R.A. (2018). Nextstrain: real-time tracking of pathogen evolution. Bioinformatics *34*, 4121–4123. https://doi.org/10.1093/BIOINFORMATICS/BTY407.

59. Hodcroft, E. (2021). CoVariants: SARS-CoV-2 Mutations and Variants of Interest. https://covariants.org/.

60. Paul, S., Grifoni, A., Peters, B., and Sette, A. (2019). Major histocompatibility complex binding, eluted ligands, and immunogenicity: benchmark testing and predictions. Front. Immunol. *10*, 3151. https://doi.org/10.3389/FIMMU.2019.03151.

61. Abelin, J.G., Keskin, D.B., Sarkizova, S., Hartigan, C.R., Zhang, W., Sidney, J., Stevens, J., Lane, W., Zhang, G.L., Eisenhaure, T.M., et al. (2017). Mass spectrometry profiling of HLA-associated peptidomes in mono-allelic cells enables more accurate epitope prediction. Immunity *46*, 315–326. https://doi.org/10.1016/J.IMMUNI.2017.02.007.

62. Grenga, L., Gallais, F., Pible, O., Gaillard, J.C., Gouveia, D., Batina, H., Bazaline, N., Ruat, S., Culotta, K., Miotello, G., et al. (2020). Shotgun proteomics analysis of SARS-CoV-2-infected cells and how it can optimize whole viral particle antigen production for vaccines. Emerg. Microbes Infect., 1712–1721. https://doi.org/10.1080/22221751.2020.1791737.

63. Finkel, Y., Mizrahi, O., Nachshon, A., Weingarten-Gabbay, S., Morgenstern, D., Yahalom-Ronen, Y., Tamir, H., Achdout, H., Stein, D., Israeli, O., et al. (2020). The coding capacity of SARS-CoV-2. Nature *589*, 125–130. https://doi.org/10.1038/s41586-020-2739-1.

64. Yan, R., Zhang, Y., Li, Y., Xia, L., Guo, Y., and Zhou, Q. (2020). Structural basis for the recognition of SARS-CoV-2 by full-length human ACE2. Science *367*, 1444–1448. https://doi.org/10.1126/science.abb2762.

65. Lan, J., Ge, J., Yu, J., Shan, S., Zhou, H., Fan, S., Zhang, Q., Shi, X., Wang, Q., Zhang, L., and Wang, X. (2020). Structure of the SARS-CoV-2 spike receptor-binding domain bound to the ACE2 receptor. Nature *581*, 215–220. https://doi.org/10.1038/s41586-020-2180-5.

66. Shang, J., Ye, G., Shi, K., Wan, Y., Luo, C., Aihara, H., Geng, Q., Auerbach, A., and Li, F. (2020). Structural basis of receptor recognition by SARS-CoV-2. Nature *581*, 221–224. https://doi.org/10.1038/s41586-020-2179-y.

67. Starr, T.N., Greaney, A.J., Hilton, S.K., Ellis, D., Crawford, K.H.D., Dingens, A.S., Navarro, M.J., Bowen, J.E., Tortorici, M.A., Walls, A.C., et al. (2020). Deep mutational scanning of SARS-CoV-2 receptor binding domain reveals constraints on folding and ACE2 binding. Cell *182*, 1295–1310.e20. https://doi.org/10.1016/J.CELL.2020.08.012.

68. Starr, T.N., Czudnochowski, N., Liu, Z., Zatta, F., Park, Y.J., Addetia, A., Pinto, D., Beltramello, M., Hernandez, P., Greaney, A.J., et al. (2021). SARS-CoV-2 RBD antibodies that maximize breadth and resistance to escape. Nature, 97–102. https://doi.org/10.1038/s41586-021-03807-6.

69. Kidd, M., Richter, A., Best, A., Cumley, N., Mirza, J., Percival, B., Mayhew, M., Megram, O., Ashford, F., White, T., et al. (2021). S-variant SARS-CoV-2 lineage B1.1.7 is associated with significantly higher viral load in samples tested by TaqPath polymerase chain reaction. J. Infect. Dis. *223*, 1666–1670. https://doi.org/10.1093/INFDIS/JIAB082.

70. Leung, K., Shum, M.H., Leung, G.M., Lam, T.T., and Wu, J.T. (2021). Early transmissibility assessment of the N501Y mutant strains of SARS-CoV-2 in the United Kingdom, October to November 2020. Euro Surveill. *26*, 2002106. https://doi.org/10.2807/1560-7917.ES.2020.26.1.2002106/CITE/PLAINTEXT.

71. Lan, J., He, X., Ren, Y., Wang, Z., Zhou, H., Fan, S., Zhu, C., Liu, D., Shao, B., Liu, T.Y., et al. (2022). Structural insights into the SARS-CoV-2 Omicron

RBD-ACE2 interaction. Cell Res., 593–595. https://doi.org/10.1038/s41422-022-00644-8.

72. Mannar, D., Saville, J.W., Zhu, X., Srivastava, S.S., Berezuk, A.M., Tuttle, K.S., Marquez, A.C., Sekirov, I., and Subramaniam, S. (2022). SARS-CoV-2 Omicron variant: Antibody evasion and cryo-EM structure of spike protein–ACE2 complex. Science *375*, 760–764. https://doi.org/10.1126/SCIENCE.ABN7760.

73. Lu, L., Chu, A.W.H., Zhang, R.R., Chan, W.M., Ip, J.D., Tsoi, H.W., Chen, L.L., Cai, J.P., Lung, D.C., Tam, A.R., et al. (2021). The impact of spike N501Y mutation on neutralizing activity and RBD binding of SARS-CoV-2 convalescent serum. EBioMedicine *71*, 103544. https://doi.org/10.1016/j.ebiom.2021.103544.

74. Starr, T.N., Greaney, A.J., Dingens, A.S., and Bloom, J.D. (2021). Complete map of SARS-CoV-2 RBD mutations that escape the monoclonal antibody LY-CoV555 and its cocktail with LY-CoV016. Cell Rep. Med. *2*, 100255. https://doi.org/10.1016/J.XCRM.2021.100255.

75. Focosi, D., Novazzi, F., Genoni, A., Dentali, F., Gasperina, D.D., Baj, A., and Maggi, F. (2021). Emergence of SARS-COV-2 spike protein escape mutation Q493R after treatment for COVID-19 - Volume 27, Number 10—October 2021 - Emerging Infectious Diseases journal - CDC. Emerg. Infect. Dis. *27*, 2728–2731. https://doi.org/10.3201/EID2710.211538.

76. Okba, N.M.A., Müller, M.A., Li, W., Wang, C., Geurtsvankessel, C.H., Corman, V.M., Lamers, M.M., Sikkema, R.S., de Bruin, E., Chandler, F.D., et al. (2020). Severe acute respiratory syndrome Coronavirus 2−specific antibody responses in Coronavirus disease patients - Volume 26, Number 7—July 2020 - Emerging Infectious Diseases journal - CDC. Emerg. Infect. Dis. *26*, 1478–1488. https://doi.org/10.3201/EID2607.200841.

77. Heffron, A.S., McIlwain, S.J., Amjadi, M.F., Baker, D.A., Khullar, S., Armbrust, T., Halfmann, P.J., Kawaoka, Y., Sethi, A.K., Palmenberg, A.C., et al. (2021). The landscape of antibody binding in SARS-CoV-2 infection. PLoS Biol. *19*, e3001265. https://doi.org/10.1371/JOURNAL.PBIO.3001265.

78. Sikora, M., von Bülow, S., Blanc, F.E.C., Gecht, M., Covino, R., and Hummer, G. (2021). Computational epitope map of SARS-CoV-2 spike protein. PLoS Comput. Biol. *17*, e1008790. https://doi.org/10.1371/JOURNAL.PCBI.1008790.

79. Cai, Y., Zhang, J., Xiao, T., Peng, H., Sterling, S.M., Walsh, R.M., Rawson, S., Rits-Volloch, S., and Chen, B. (2020). Distinct conformational states of SARS-CoV-2 spike protein. Science *369*, 1586–1592. https://doi.org/10.1126/SCIENCE.ABD4251.

80. GeurtsvanKessel, C.H., Geers, D., Schmitz, K.S., Mykytyn, A.Z., Lamers, M.M., Bogers, S., Scherbeijn, S., Gommers, L., Sablerolles, R.S.G., Nieuwkoop, N.N., et al. (2022). Divergent SARS-CoV-2 Omicron-reactive T and B cell responses in COVID-19 vaccine recipients. Sci. Immunol. *7*, eabo2202. https://doi.org/10.1126/SCIIMMUNOL.ABO2202.

81. Grifoni, A., Sidney, J., Vita, R., Peters, B., Crotty, S., Weiskopf, D., and Sette, A. (2021). SARS-CoV-2 human T cell epitopes: adaptive immune response against COVID-19. Cell Host Microbe *29*, 1076–1092. https://doi.org/10.1016/J.CHOM.2021.05.010.

82. Chalkias, S., Harper, C., Vrbicky, K., Walsh, S.R., Essink, B., Brosz, A., McGhee, N., Tomassini, J.E., Chen, X., Chang, Y., et al. (2022). A bivalent Omicron-containing booster vaccine against Covid-19. N. Engl. J. Med. *387*, 1279–1291. https://doi.org/10.1056/NEJMOA2208343.

83. Wang, Q., Bowen, A., Valdez, R., Gherasim, C., Gordon, A., Liu, L., and Ho, D.D. (2023). Antibody response to Omicron BA.4–BA.5 bivalent booster. N. Engl. J. Med. *388*, 567–569. https://doi.org/10.1056/NEJMC2213907.

84. Dutta, N.K., Mazumdar, K., and Gordy, J.T. (2020). The Nucleocapsid Protein of SARS–CoV-2: a Target for Vaccine Development. J. Virol. *94*, 00647-20. https://doi.org/10.1128/JVI.00647-20.

85. Bulek, A.M., Madura, F., Fuller, A., Holland, C.J., Schauenburg, A.J. a, Sewell, A.K., Rizkallah, P.J., and Cole, D.K. (2012). TCR/pMHC optimized protein crystallization screen. J. Immunol. Methods *382*, 203–210. https://doi.org/10.1016/j.jim.2012.06.007.

86. Theaker, S.M., Rius, C., Greenshields-Watson, A., Lloyd, A., Trimby, A., Fuller, A., Miles, J.J., Cole, D.K., Peakman, M., Sewell, A.K., et al. (2016). T-cell libraries allow simple parallel generation of multiple peptide-specific human T-cell clones. J. Immunol. Methods *430*, 43–50. https://doi.org/10.1016/J.JIM.2016.01.014.

87. Williams, C.J., Headd, J.J., Moriarty, N.W., Prisant, M.G., Videau, L.L., Deis, L.N., Verma, V., Keedy, D.A., Hintze, B.J., Chen, V.B., et al. (2018). MolProbity: More and better reference data for improved all-atom structure validation. Protein Sci. *27*, 293–315. https://doi.org/10.1002/PRO.3330.

88. Frayser, M., Sato, A.K., Xu, L., and Stern, L.J. (1999). Empty and peptide-loaded class II major histocompatibility complex proteins produced by expression in Escherichia coli and folding in vitro. Protein Expr. Purif. *15*, 105–114. https://doi.org/10.1006/prep.1998.0987.

89. Studier, F.W. (2005). Protein production by auto-induction in high-density shaking cultures. Protein Expr. Purif. *41*, 207–234. https://doi.org/10.1016/J.PEP.2005.01.016.

90. Waskom, M. (2021). seaborn: statistical data visualization. J. Open Source Softw. *6*, 3021. https://doi.org/10.21105/JOSS.03021.

91. Winter, G., Lobley, C.M.C., and Prince, S.M. (2013). Decision making in xia2. Acta Crystallogr. D Biol. Crystallogr. *69*, 1260–1273. https://doi.org/10.1107/S0907444913015308/BA5195SUP1.PDF.

92. Winter, G., Waterman, D.G., Parkhurst, J.M., Brewster, A.S., Gildea, R.J., Gerstel, M., Fuentes-Montero, L., Vollmar, M., Michels-Clark, T., Young, I.D., et al. (2018). DIALS: implementation and evaluation of a new integration package. Acta Crystallogr. D Struct. Biol. *74*, 85–97. https://doi.org/10.1107/S2059798317017235.

93. Kabsch, W., and IUCr. (2010). XDS. Acta Crystallogr. D Biol. Crystallogr. *66*, 125–132. https://doi.org/10.1107/S0907444909047337.

94. McCoy, A.J. (2007). Solving structures of protein complexes by molecular replacement with Phaser. Acta Crystallogr. D Biol. Crystallogr. *63*, 32–41. https://doi.org/10.1107/S0907444906045975.

95. Liebschner, D., Afonine, P.V., Baker, M.L., Bunkóczi, G., Chen, V.B., Croll, T.I., Hintze, B., Hung, L.W., Jain, S., McCoy, A.J., et al. (2019). Macromolecular structure determination using X-rays, neutrons and electrons: Recent developments in Phenix. Acta Crystallogr. D Struct. Biol. *75*, 861–877. https://doi.org/10.1107/S2059798319011471.

96. Emsley, P., Lohkamp, B., Scott, W.G., and Cowtan, K. (2010). Features and development of Coot. Acta Crystallogr. D Biol. Crystallogr. *66*, 486–501. https://doi.org/10.1107/S0907444910007493.

97. Long, F., Nicholls, R.A., Emsley, P., Graǽulis, S., Merkys, A., Vaitkus, A., and Murshudov, G.N. (2017). AceDRG: a stereochemical description generator for ligands. Acta Crystallogr. D Struct. Biol. *73*, 112–122. https://doi.org/10.1107/S2059798317000067/BA5260SUP2.TXT.

98. Agirre, J., Atanasova, M., Bagdonas, H., Ballard, C.B., Baslé, A., Beilsten-Edmands, J., Borges, R.J., Brown, D.G., Burgos-Mármol, J.J., Berrisford, J.M., et al. (2023). The CCP4 suite: integrative software for macromolecular crystallography. Acta Cryst D *79*, 449–461. https://doi.org/10.1107/S2059798323003595.

99. Virtanen, P., Gommers, R., Oliphant, T.E., Haberland, M., Reddy, T., Cournapeau, D., Burovski, E., Peterson, P., Weckesser, W., Bright, J., et al. (2020). SciPy 1.0: fundamental algorithms for scientific computing in Python. Nat. Methods *17*, 261–272. https://doi.org/10.1038/s41592-019-0686-2.

100. Hunter, J.D. (2007). Matplotlib: A 2D graphics environment. Comput. Sci. Eng. *9*, 90–95. https://doi.org/10.1109/MCSE.2007.55.

STAR★METHODS

KEY RESOURCES TABLE

REAGENT or RESOURCE	SOURCE	IDENTIFIER
Antibodies		
anti-CD3-APC (clone UCHT1)	Biolegend	Cat#300412; RRID: AB_314066
anti-CD4-BV421 (clone RPA-T4)	Biolegend	Cat#300532; RRID: AB_10965645
anti-CD8-BV786 (RPA-T8)	BD Bioscience	Cat#563823; RRID: AB_2687487
anti-IFN-γ-FITC (clone B27)	Biolegend	Cat#506504; RRID: AB_315437
anti-TNF-α-PE-Cy7 (clone MAb11)	Biolegend	Cat#502930; RRID: AB_2204079
anti-HLA-DR (clone L243)	Biolegend	Cat#307667; RRID: AB_2800798
Bacterial and virus strains		
Escherichia coliBL21(DE3) Chemically Competent cells	ThermoFisher Scientific	Cat#C600003
Biological samples		
Peripheral blood of local HLA-DR1^{+} and DR4^{+} individuals	This manuscript	N/A
Chemicals, peptides, and recombinant proteins		
Synthetic peptide S_{1-15} MFVFLVLLPLVSSQC	Peptide Protein Research	Custom synthesis
Synthetic peptide $S_{167-181}$ TFEYVSQPFLMDLEG	Peptide Protein Research	Custom synthesis
Synthetic peptide $S_{235-249}$ ITRFQTLLALHRSYL	Peptide Protein Research	Custom synthesis
Synthetic peptide $S_{241-255}$ LLALHRSYLTPGDSS	Peptide Protein Research	Custom synthesis
Synthetic peptide $S_{339-353}$ GEVFNATRFASVYAW	Peptide Protein Research	Custom synthesis
Synthetic peptide $S_{344-358}$ ATRFASVYAWNRKRI	Peptide Protein Research	Custom synthesis
Synthetic peptide $S_{486-505}$ FNCYFPLQSYGFQPTN GVGY	Peptide Protein Research	Custom synthesis
Synthetic peptide $S_{511-530}$ VVLSFELLHAPATVCG PKKS	Peptide Protein Research	Custom synthesis
Synthetic peptide $S_{512-526}$ VLSFELLHAPATVCG	Peptide Protein Research	Custom synthesis
Synthetic peptide $S_{749-763}$ CSNLLLQYGSFCTQL	Peptide Protein Research	Custom synthesis
Synthetic peptide $_{S761-775}$ TQLNRALTGIAVEQD	Peptide Protein Research	Custom synthesis
Synthetic peptide $S_{818-832}$ IEDLLFNKVTLADAG	Peptide Protein Research	Custom synthesis
Synthetic peptide $S_{866-880}$ TDEMIAQYTSALLAG	Peptide Protein Research	Custom synthesis

(Continued on next page)

Continued

REAGENT or RESOURCE	SOURCE	IDENTIFIER
Synthetic peptide $S_{990-1004}$ EVQIDRLITGRLQSL	Peptide Protein Research	Custom synthesis
Synthetic peptide $S_{1015-1029}$ AAEIRASANLAATKM	Peptide Protein Research	Custom synthesis
Synthetic peptide E_{56-70} FYVYSRVKNLNSSRV	Peptide Protein Research	Custom synthesis
Synthetic peptide $M_{176-190}$ LSYYKLGASQRVAGD	Peptide Protein Research	Custom synthesis
Synthetic peptide N_{49-63} TASWFTALTQHGKED	Peptide Protein Research	Custom synthesis
Synthetic peptide $N_{108-122}$ WYFYYLGTGPEAGLP	Peptide Protein Research	Custom synthesis
Synthetic peptide $N_{127-141}$ KDGIIWVATEGALNT	Peptide Protein Research	Custom synthesis
Synthetic peptide $N_{224-238}$ LDRLNQLESKMSGKG	Peptide Protein Research	Custom synthesis
Synthetic peptide $N_{328-342}$ GTWLTYTGAIKLDDK	Peptide Protein Research	Custom synthesis
Synthetic peptide $NSP3_{1350-1364}$ KSAFYILPSIISNEK	Peptide Protein Research	Custom synthesis
Synthetic peptide $NSP6_{3801-3815}$ NRYFRLTLGVYDYLV	Peptide Protein Research	Custom synthesis
Synthetic peptide $NSP12_{5019-5033}$ PNMLRIMASLVLARK	Peptide Protein Research	Custom synthesis
Synthetic peptide $NSP14_{6420-6434}$ LDAYNMMISAGFSLW	Peptide Protein Research	Custom synthesis
Synthetic peptide $ORF6_{13-27}$ EILLIIMRTFKVSIW	Peptide Protein Research	Custom synthesis
Synthetic peptide $ORF8_{3-17}$ FLVFLGIITTVAAFH	Peptide Protein Research	Custom synthesis
Synthetic peptide $ORF8_{43-57}$ SKWYIRVGARKSAPL	Peptide Protein Research	Custom synthesis
Synthetic peptide $S_{486-505}$ (Omicron BA.1) FNCYFPLRSY SFRPTYGVGH	Peptide Protein Research	Custom synthesis
Synthetic peptide S761-775 (Omicron BA.1) TQLKRALTGIAVEQD	Peptide Protein Research	Custom synthesis
Synthetic peptide CLIP105–117 SKMRMATPLLMQA	Peptide Protein Research	Custom synthesis
Synthetic peptide N-terminally biotinylated CLIP99–117 bt-LPKPPKPVSKMRMAT PLLMQA	Peptide Protein Research	Custom synthesis
Lymphoprep	Axis-Shield	Cat#1114547
Pierce^{TM} Protein A IgG Plus Orientation Kit, 2 mL	Thermo Scientific	Cat#44893
Human recombinant IL-2 (Proleukin®)	Cardiff and Vale University Health Board Pharmacy	N/A
CTL Test Plus media	CTL Europe	Cat#CTLTP-005
RPMI 1640	ThermoFisher Scientific	Cat#11875093
**Critical commercial assays**		
Streptavidin-HRP	R&D Systems (bio-techne)	Cat# DY998

(*Continued on next page*)

Continued		
REAGENT or RESOURCE	SOURCE	IDENTIFIER
BD OptEIA^{TM} TMB Substrate Reagent Set	BD Biosciences	Cat# 555214; RRID: AB_2869044
ELISpot Flex: Human IFN-γ (ALP)	Mabtech	Cat# 3420-2A
ELISPOT plates	Sigma-Aldrich	Cat# MAIPS4510
TOPS Crystallization Screen	Molecular Dimensions	Custom product. Bulek et al.[85]
PACT premier^{TM} HT-96 Crystallization Screen	Molecular Dimensions	Cat# MD1-36
MojoSort^{TM} Human CD4 T Cell Isolation Kit	Biolegend	Cat# 480010
FIX & PERM Cell Fixation & Cell Permeabilization	BD Biosciences	Cat# 554714
LIVE/DEAD^{TM} Fixable Aqua	ThermoFisher Scientific	Cat# L34957
Deposited data		
HLA-DR1-$S_{486-505}$	This manuscript	PDB: 8CMB
HLA-DR1-$S_{511-530}$	This manuscript	PDB: 8CMC
HLA-DR1-$S_{761-775}$	This manuscript	PDB: 8CMD
HLA-DR1-$M_{176-190}$	This manuscript	PDB: 8CME
HLA-DR1-$nsp3_{1350-1364}$	This manuscript	PDB: 8CMF
HLA-DR1-$nsp14_{6420-6434}$	This manuscript	PDB: 8CMG
HLA-DR1-$S_{486-505}^{Omicron\ (BA.1)}$	This manuscript	PDB: 8CMH
HLA-DR1-$S_{761-775}^{Omicron\ (BA.1)}$	This manuscript	PDB: 8CMI
3,013-genome subsample of the ncov/gisaid/global/all-time dataset	GISAID contributors	Table S3
3,214 genome subset of the ncov/gisaid/oceania/all-time dataset	GISAID contributors	Table S3
Experimental models: Cell lines		
T2 (174 x CEM.T2)	ATCC	CRL-1992^{TM}; RRID: CVCL_2211
T2-DR1 (DRA*0101, DRB1*0101 lentivirally transduced)	Cole, Godkin labs	Theaker et al.[86]
Recombinant DNA		
pGEM-T7	Promega	Cat#A3600
pGEM-T7-DRA1*0101	Cole, Godkin labs	Greenshields-Watson et al.[22]
pGEM-T7-DRB1*0101	Cole, Godkin labs	Greenshields-Watson et al.[22]
Software and algorithms		
PyMOL (v2.5 open-source build)	Maintained by Schrodinger	RRID:SCR_000305
Phaser	Phenix Online	RRID:SCR_014219
Phenix v1.20	Phenix Online	RRID:SCR_014224
CCP4	Collaborative Computational Project No. 4	RRID:SCR_007255
Molprobity	Duke University[87]	RRID:SCR_014226
Xia2 pipeline	Diamond Light Source (DLS)	RRID:SCR_015746
Dials	DLS, Lawrence Berkeley National Laboratory and STFC	https://dials.github.io/about.html
XDS program package	Max Planck Institute for Medical Research	RRID:SCR_015652
COOT v0.9.6	MRC Laboratory of Molecular Biology	RRID:SCR_014222
AceDRG	Collaborative Computational Project No. 4	RRID:SCR_007255

(*Continued on next page*)

Continued

REAGENT or RESOURCE	SOURCE	IDENTIFIER
Nextstrain interactive portal	Nextstrain (Hadfield et al.)[58]	https://nextstrain.org/ncov/gisaid/global/6m
FlowJo	FlowJo LLC	RRID:SCR_008520
NetMHCpanII v4.1	DTU Health Tech Department of Health Technology	https://services.healthtech.dtu.dk/services/NetMHCpan-4.1/
Matplotlib	The Matplotlib development team	RRID:SCR_008624
Seaborn	Michael Waskom	RRID:SCR_018132
SciPy	The SciPy Project	RRID:SCR_008058

RESOURCE AVAILABILITY

Lead contact

Further information and requests for resources and reagents should be directed to and will be fulfilled by the lead contact, Andrew Godkin (godkinaj@cardiff.ac.uk).

Materials availability

Cell lines and protein expression plasmids are available from the lead contact on request. All other reagents are available to purchase from commercial suppliers.

Data and code availability

- Final model co-ordinates and structure factors were submitted to the Protein Data Bank under accession codes: DR1-$S_{486-505}$ = PDB: 8CMB, DR1-$S_{511-530}$ = PDB: 8CMC, DR1-$S_{761-775}$ = PDB: 8CMD, DR1-$M_{176-190}$ = PDB: 8CME, DR1-$nsp3_{1350-1364}$ = PDB: 8CMF, DR1-$nsp14_{6420-6434}$ = PDB: 8CMG, DR1-$S_{486-505}^{Omicron\ (BA.1)}$ = PDB: 8CMH, DR1-$S_{761-775}^{Omicron\ (BA.1)}$ = PDB: 8CMI. Raw data from all non-structural related figures are available from Mendeley Data: https://doi.org/10.17632/5hh7rwwttm.2.
- Structural analysis code is available at: https://zenodo.org/record/8047547.
- Any additional information required to reanalyze the data reported in this paper is available from the lead contact upon request.

EXPERIMENTAL MODEL AND STUDY PARTICIPANT DETAILS

Ethical approval, participant recruitment & consenting

Ethical approval was obtained from the Medical School Research Ethics Committee, Cardiff University to study "Immune surveillance and recognition of infection and cancer" in local healthy individuals. Study participants were recruited via an open advertisement for volunteers within the Heath Park Campus, Cardiff, UK. Informed consent was obtained from all volunteering donors. Donors were aged 31-59 years old (mean age 39 years old) at time of their first donated sample. The cohort consisted of four females and four males. Ethnicity was not recorded during consenting. Participation in the UK COVID-19 vaccination campaign was not a requirement for study participation. HLA-typing was performed by the Welsh Transplantation and Immunogenetics Laboratory (Pontyclun, UK). Blood donations to test the Wuhan HU-1 peptide panel were taken between November 2020 and November 2021. Blood donations to compare Wuhan HU-1 and Omicron (BA.1) peptides were taken between March 2022 and October 2022.

Generation of cultured T cell lines from donor PBMCs

PBMCs were isolated over ficoll gradient (Lymphoprep, Axis-Shield). Subsequent isolated PBMCs were cultured at 2×10^6 cells/mL in CTL Test Plus (CTL Europe), 2 mM L-glutamine, 100 U/mL penicillin & 100 μg/mL streptomycin (Sigma-Aldrich) media in 96-well plates incubated at 37 °C, 5 % CO_2. Cultured T cell lines were generated by addition of 5 μg/mL of candidate test peptide at day 0. Cultured cells were replenished with fresh media containing 20 IU/mL IL-2 on days 3,7 & 10 before harvest at day 12-14.

For long term $CD4^+$ T cell lines with identified specificity, cells were first sorted using a MojoSort^{TM} Human CD4 T Cell Isolation Kit (Biolegend, UK) according to manufacturer's instructions. Enriched $CD4^+$ T cells were then expanded using co-culture with irradiated T2 cells (detailed below). T2-DR1 cells were loaded with 2 μg/mL of relevant peptides for 2 hrs before washing and irradiating. $CD4^+$ T cells were co-cultured with irradiated peptide loaded T2-DR1 (as APCs) and irradiated autologous PBMCs (as feeder cells) and analysed for functionality 14 days after restimulation.

Cell lines

T2 cells (T2 (174 x CEM.T2) - CRL-1992) transduced with HLA-DR1 (HLA-DRA*0101, DRB1*0101) were generated previously and denoted as T2-DR1 cells.[22,86] T2-DR1 cells were maintained in suspension at 37 °C, 5 % CO_2 in RPMI-1640 media (GIBCO)

supplemented with 10% heat inactivated fetal calf serum (gibco), 2 mM l-glutamine, 100 U/mL penicillin, 100 μg/mL streptomycin (all gibco/ThermoFisher Scientific). Cells were passaged every two to three days, maintaining cell density between $1x10^6$ and $1x10^7$ cells/mL.

METHOD DETAILS

Production of peptide-HLA-DR1 proteins

HLA-DR1 (HLA-DRA*0101 & HLA-DRB1*0101) proteins were expressed and refolded *in vitro* from inclusion bodies as previously described.[50,88] HLA-DRA and –DRB chains were produced separately in BL21(DE3) *Escherichia coli* cells via autoinduction expression.[89] Inclusion bodies were extracted and solubilized in 50 mM Tris, 8 M Urea, 2 mM EDTA, 100 mM NaCl, 1 mM DTT. Peptide-HLA-DR1 molecules were refolded by rapid dilution of HLA-DRA & -DRB inclusion bodies into refold buffer (20 mM TRIS pH 8.5, 30 % w/v glycerol, 1 mM EDTA, 20 mM NaCl, 0.3 mM cystamine & 0.6 mM cysteamine) in the presence of synthetically synthesized peptides (Peptide Protein Research Ltd.) at final concentrations of 10 μg/mL for each –DR chain and 0.5 μg/mL of peptide. Refolds were incubated at 4 °C for 2-3 days before sequential diafiltration with 10 mM Tris pH 8.1, 150 mM NaCl and 10 mM Tris pH 8.1 using 30 kDa MWCO filtration devices (Sartorius). Buffer exchanged peptide-HLA-DR1 samples were purified via 1) anion exchange (HiTrap® Q High Performance) attached to an AKTA Pure FPLC system (Cytiva), 2) affinity chromatography purified using the conformationally specific anti-HLA-DR antibody clone L243 chemically crosslinked to Protein A gravity columns (Thermo Scientific) and 3) size-exclusion chromatography using a Superdex®200 Increase 10/300 GL column (Cytiva). Final samples were purified into a buffer solution of 10 mM Tris pH 8.1, 150 mM NaCl. Sample purity was analyzed by SDS-PAGE.

HLA-II peptide binding assays

HLA-DR1 proteins were expressed as above, refolded with $CLIP_{105–117}$ (SKMRMATPLLMQA), henceforth DR1-$CLIP_{105–117}$. Competitive inhibition HLA-DR1 binding assays were performed as previously described.[37,50] Peptide exchange reactions were prepared in 20 mM MES pH 5, 140 mm NaCl, 0.02% NaN_3. Each reaction contained 0.1 μg of refolded DR1-$CLIP_{105–117}$, 45 nM N-terminally biotinylated $CLIP_{99–117}$ (bt-LPKPPKPVSKMRMATPLLMQA) marker peptide and the candidate test peptide. Candidate test peptides were 10-fold serially diluted (10^{-4}-10^{-10} M) and added to the exchange reaction before incubation overnight at 37 °C. After incubation, exchange reactions were neutralized with 1 M Tris, 10 % BSA, 1 % Tween, 0.02 % NaN_3, pH 8 and transferred to high-bind ELISA plates coated with the anti-HLA-DR antibody clone L243. After capture, biotinylated $CLIP_{99–117}$ was detected using streptavidin-HRP (R&D Systems) and colorimetric HRP substrate reagent (BD Bioscience).

Peptide immunogenicity analysis

Short-term T cell lines cultured after day 12 were directly restimulated with candidate peptide and assayed via IFN-γ ELISpot assay. T cell lines were washed, counted and plated in duplicate onto anti-IFN-γ coated PVDF filter membrane plates (Mabtech) with addition of 5 μg/mL candidate peptide. For each T cell line, response to restimulation with peptide was compared to a no peptide (media) control. Plates were incubated overnight (~16 hrs.) at 37 °C, 5% CO_2 before developing as described by manufacturer protocol. Developed plates were imaged and spot forming cells (sfcs) counted using an ImmunoSpot S6 Ultra (CTL Europe).

For long-term T cell lines, post-12 days were restimulated with candidate peptide-pulsed T2-DR1 cells (generated as described above). T2-DR1 cells were first pulsed with 5 μg/mL of candidate peptide for 2 hrs., washed to remove unbound peptide, then co-cultured with T cells in the presence of 10 μg/mL Brefeldin A. Co-cultured activation assays were incubated overnight (~16 hrs.) at 37 °C, 5% CO_2. Unpulsed T2-DR1 cells (no peptide/media) were used as negative control. Following co-culture, cells were stained with LIVE/DEAD™ Fixable Aqua (ThermoFisher Scientific), anti-CD3-APC, anti-CD4-BV421 antibodies (all Biolegend) and anti-CD8-BV780 (BD Biosciences), subsequently treated with FIX & PERM Cell Fixation & Cell Permeabilization (BD Biosciences) and then stained with anti-IFN-γ-FITC & TNF-α-PE-Cy7 (both Biolegend) for 20 mins at 4 °C in the absence of light. Stained cells were analyzed on a NovoCyte 3000 (Agilent Technologies Inc.) and analyzed using FlowJo v10 (FlowJo LLC). Heatmap and swarm/box plots presented using *seaborn.*[90]

Crystallization and structure determination of peptide-HLA-DR1 complexes

Purified peptide-HLA-DR1 samples were screened for crystallization using the vapor diffusion method using screen plates dispensed by a Crystal Gryphon (Art Robbins Instruments, LLC) at drop volumes of 0.4 μL in sitting drop format. Further condition screening was performed using hanging drop experiments hand-pipetted in EasyXtal 15-Well plates (Nextal) with a drop volume of 3 μL. Peptide-HLA-DR1 complexes crystallized at 18 °C within 2 – 30 days in a variety of conditions outlined in Table S2.

Obtained crystals of sufficient size for diffraction were flash frozen in crystallization solution supplemented with either 10 % glycerol or 10 % ethylene-glycol. Crystals were exposed to x-rays at Diamond Light Source beamlines i04 (Harwell Science and Innovation Campus, Oxfordshire, United Kingdom). Diffraction data were reduced using xia2[91] which implements DIALS[92] and XDS.[93] Phases were estimated using molecular replacement implemented by PHASER[94] using various HLA-DR1 models as search models depending on space group. For each, peptide atoms were removed from search models to prevent phases biasing peptide atom placement. Details specific to each structure are detailed in Table S2. Refinement of structures was performed using iterative rounds of refinement using *phenix.refine* of the PHENIX suite[95] and manual model editing using COOT v0.9.6. TLS groups for TLS refinement were

determined using *phenix.find_tls_groups.* Progress of model quality during refinement was assessed by MolProbity.[87,96] For HLA-DR1-$S_{486-505}$ and HLA-DR1-$S_{486-505}^{Omicron (BA.1)}$, coordinates for cysteamine (2-Aminoethanethiol, PDB ligand code: DHL) were obtained from the COOT ligand dictionary after which covalent link restraints to the cysteine thiol sulfur atom were generated using AceDRG,[97] part of the CCP4 suite.[98] Subsequent refinements proceeded by defining this covalent constraint using the *refinement.-geometry_restraints.edits* parameter during *phenix.refine*.

Omit maps were calculated by performing a two macro-cycle refinement (bulk solvent and scaling, local real-space refinement, simulated annealing (cartesian), reciprocal space refinement, occupancy refinement) in the absence of all peptide atoms. This was achieved by passing the following command line parameter definitions to the *phenix.refine* default refinement strategy: *simulated_annealing=True, main.number_of_macro_cycles=2, tls=False*. Molecular visualizations of structures were generated using the open-source build of Pymol V.2.5.0 (Schrodinger, LLC). CCP4 format electron density maps were generated using *phenix.mtz2map*. Final model co-ordinates and structure factors were submitted to the Protein Data Bank under accession codes: DR1-$S_{486-505}$ = 8CMB, DR1-$S_{511-530}$ = 8CMC, DR1-$S_{761-775}$ = 8CMD, DR1-$M_{176-190}$ = 8CME, DR1-$nsp3_{1350-1364}$ = 8CMF, DR1-$nsp14_{6420-6434}$ = 8CMG, DR1-$S_{486-505}^{Omicron (BA.1)}$ = 8CMH, DR1-$S_{761-775}^{Omicron (BA.1)}$ = 8CMI.

SARS-CoV-2 mutational sequence analysis

Analysis of variant mutations was performed using the Nextstrain[58] ncov interactive visualization portal which is enabled by data from GISAID[55] and its contributors – a full acknowledgements list is shown in Table S3: References for accessed GISAID contributor data. A 3,013-genome subsample of the ncov/gisaid/global/all-time dataset was used to display the genotypic per-residue frequency of amino acid usage encompassing epitope regions over time (16th December 2019 – 10th June 2022; Accessed 14th June 2022) within the subsampled dataset. For analysis of $nsp14_{6420-6434}$ L6433F in Australasia, a 3,214 genome subset of the ncov/gisaid/oceania/all-time dataset (4th Jan 2020 – 14th June 2022; Accessed 15th June 2022) was used. Defining mutations of Variants of Concern/Variants of Interest were accessed as defined by the CoVariants portal[59] which is enabled by Nextstrain and data from GISAID.

QUANTIFICATION AND STATISTICAL ANALYSIS

HLA-II peptide binding assays

All assays were performed in triplicate (n=3 technical replicate wells) in two independent experiments (n=2). Mean, standard deviation and IC50 were calculated from a representative example. Raw absorbance data were normalized to a no-competitor peptide control to derive percentage competitive inhibition by each candidate peptide. Mean percentage competitive inhibition values and standard deviation were calculated using *SciPy.*[99] IC50 values were calculated by fitting a four-parameter log(inhibitor) response function by *math.curve_fit* of *SciPy*, using the following initial values for curve fitting: slope = SIGN(Y at XMAX – Y at XMIN), min = YMIN, max = YMAX & IC50 = X at YMID. Data were plotted and presented using *matplotlib.*[100]

ELISpot analyses

Assays were performed in duplicate peptide stimulations (n=2 technical replicate wells) at two independent two time points (n=2 peripheral blood donations per donor). For each blood donation, replicate wells were averaged (mean) and background subtracted by a no peptide control well (media) for each corresponding cultured line. Spot forming cells (sfcs) were normalized to sfcs/10,000 cells based on total number of plated cells in each assay well. The maximal response for each peptide from the two time points was taken as a maximal response. A positive response to a peptide by a donor was considered if maximal response was $\geq$ 25 sfcs/10,000 cells above background. Data were binned into no response (< 25 sfcs/10,000 cells), low (25-50) moderate (50-100) and high (100+) for the purpose of heat map visualization clarity.

To evaluate the peptide response rate in the donor cohort, the number of responses (tests) was divided by the total number of tests (i.e. number of donors multiplied by number of peptides tested) and represented as a percentage. This equated to 87 total tests for HLA-DR1^{+} donors and 135 total tests for HLA-DR4^{+} donors. A Fisher's exact test was used to evaluate an association between DR1^{+}/DR4^{+} status (groups) and the positive responses to peptides (outcomes). Significance was defined by $P < 0.01$.

Cell Reports, Volume *42*

Supplemental information

Structural definition of HLA class II-presented SARS-CoV-2 epitopes reveals a mechanism to escape pre-existing CD4$^+$ T cell immunity

Yuan Chen, Georgina H. Mason, D. Oliver Scourfield, Alexander Greenshields-Watson, Tracey A. Haigh, Andrew K. Sewell, Heather M. Long, Awen M. Gallimore, Pierre Rizkallah, Bruce J. MacLachlan, and Andrew Godkin

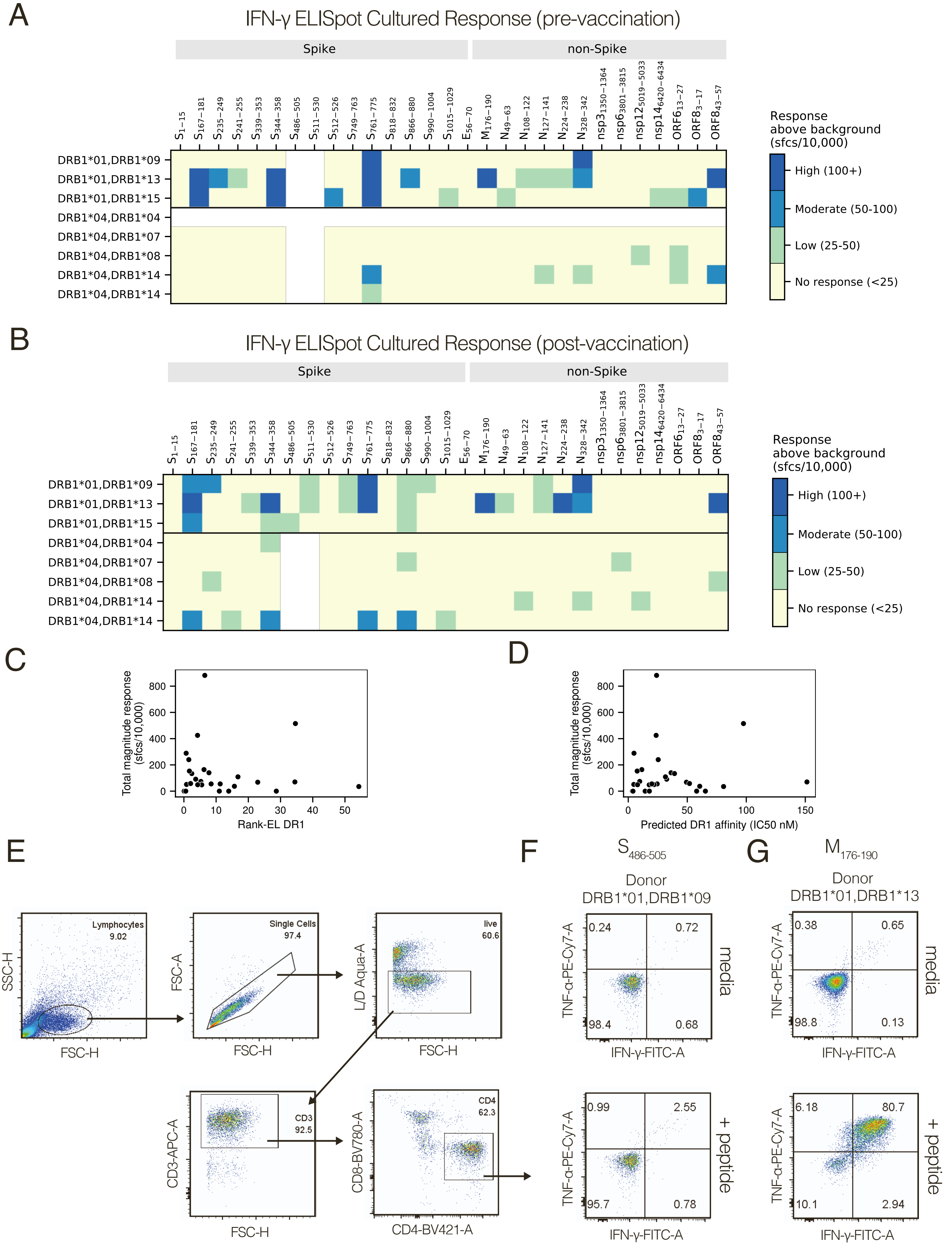
A
IFN-γ ELISpot Cultured Response (pre-vaccination)
Spike
non-Spike
Response above background (sfcs/10,000)
High (100+)
Moderate (50-100)
Low (25-50)
No response (<25)
DRB1*01,DRB1*09
DRB1*01,DRB1*13
DRB1*01,DRB1*15
DRB1*04,DRB1*04
DRB1*04,DRB1*07
DRB1*04,DRB1*08
DRB1*04,DRB1*14
DRB1*04,DRB1*14
B
IFN-γ ELISpot Cultured Response (post-vaccination)
C
Total magnitude response (sfcs/10,000)
Rank-EL DR1
D
Predicted DR1 affinity (IC50 nM)
E
Lymphocytes 9.02
Single Cells 97.4
live 60.6
CD3 92.5
CD4 62.3
SSC-H
FSC-H
FSC-A
L/D Aqua-A
CD3-APC-A
CD8-BV780-A
CD4-BV421-A
F
Donor DRB1*01,DRB1*09
G
Donor DRB1*01,DRB1*13
TNF-α-PE-Cy7-A
IFN-γ-FITC-A
media
+ peptide

Figure S1 – Immunogenicity of selected SARS-CoV-2 peptides in HLA-DR1$^+$ and HLA-DR4$^+$ donors. A) Heat map summary of IFN-γ ELISpot responses to overnight restimulation with SARS-CoV-2 peptides (15 – 20mer length) following 12-day culture of PBMCs in presence of candidate peptide before vaccination. Donors are grouped by HLA-DR1$^+$ and HLA-DR4$^+$ status. ELISPOT assays performed in duplicate. Responses were background subtracted (media only), normalized to sfcs/10,000 cells and binned into low, moderate and high responders (cut-offs and colors indicated inset). **B)** Heatmap summary of IFN-γ ELISpot responses as described in A) following SARS-CoV-2 vaccination. **C)** Scatter plot summary of total magnitude response to each peptide (summated maximal response by each donor for each peptide) in HLA-DR1$^+$ donors against predicted rank eluted likelihood (Rank-EL) as determined by NetMHCIIpan v4.1. No correlation was observed. **D)** Scatter plot summary of total magnitude response to each peptide in HLA-DR1$^+$ donors against predicted binding affinity (IC_{50} nM) as determined by NetMHCIIpan v4.1. No correlation was observed. **E)** Representative gating strategy analyzing lymphocyte gate, single cells, live cells, CD3$^+$, CD4$^+$ cells. Gating strategy applied in D&E. **F)** TNF-α & IFN-γ intracellular cytokine staining of $S_{761-775}$ T cell line in response to $S_{761-775}$ pulsed T2-DR1$^+$ cells. Response of T cell/APC co-culture in absence of peptide (media) control is shown. **G)** TNF-α & IFN-γ intracellular cytokine staining of $M_{176-190}$ T cell line in response to $M_{176-190}$ pulsed T2-DR1$^+$ cells. Response of T cell/APC co-culture in absence of peptide (media) control is shown.

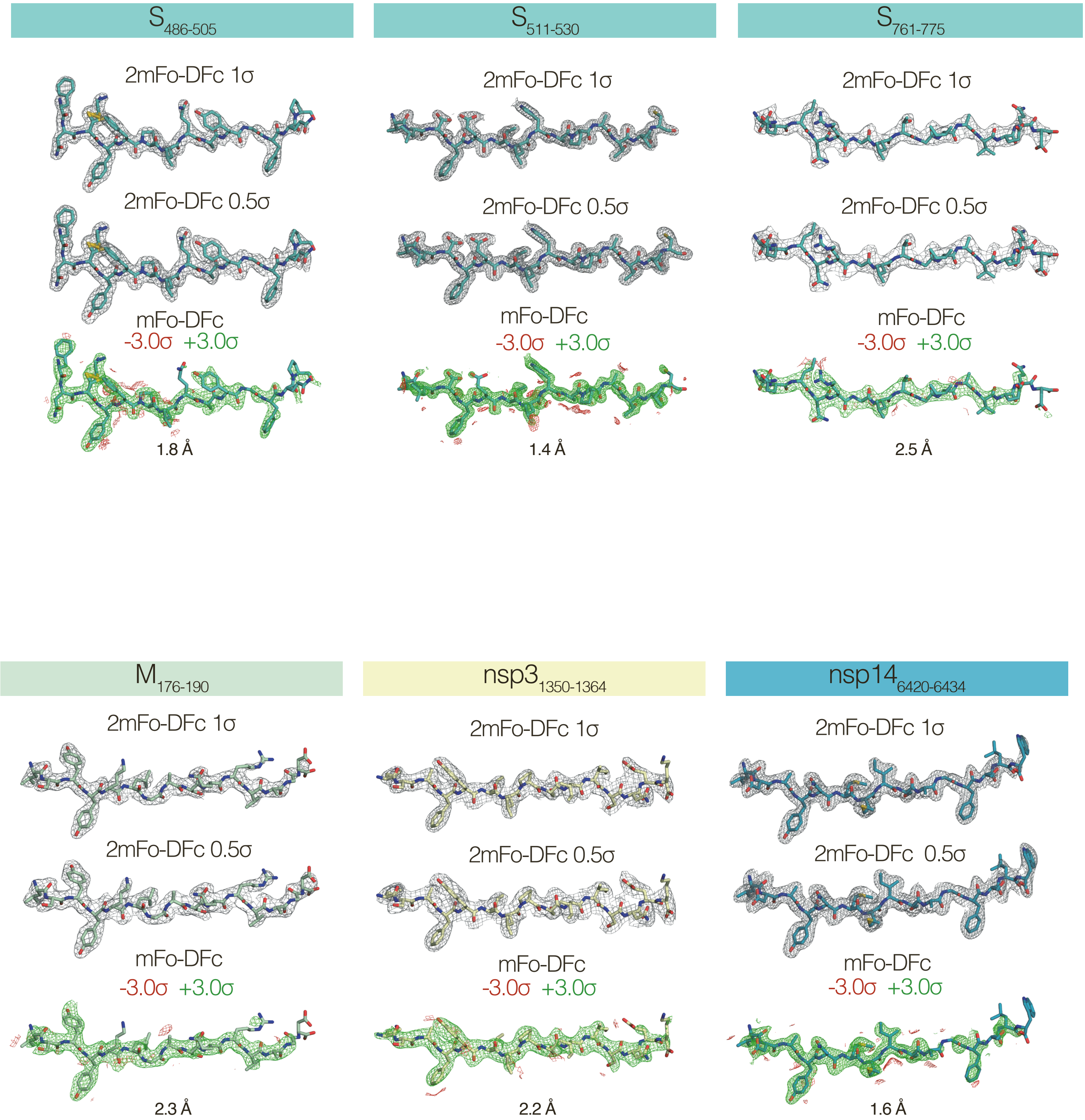

$S_{486-505}$
2mFo-DFc 1σ
2mFo-DFc 0.5σ
mFo-DFc
-3.0σ +3.0σ
1.8 Å
$S_{511-530}$
2mFo-DFc 1σ
2mFo-DFc 0.5σ
mFo-DFc
-3.0σ +3.0σ
1.4 Å
$S_{761-775}$
2mFo-DFc 1σ
2mFo-DFc 0.5σ
mFo-DFc
-3.0σ +3.0σ
2.5 Å
$M_{176-190}$
2mFo-DFc 1σ
2mFo-DFc 0.5σ
mFo-DFc
-3.0σ +3.0σ
2.3 Å
$nsp3_{1350-1364}$
2mFo-DFc 1σ
2mFo-DFc 0.5σ
mFo-DFc
-3.0σ +3.0σ
2.2 Å
$nsp14_{6420-6434}$
2mFo-DFc 1σ
2mFo-DFc 0.5σ
mFo-DFc
-3.0σ +3.0σ
1.6 Å

Figure S2 – Electron density maps and omit map analysis of peptide-HLA-DR1 structures. 2mFo-DFc contoured at 1.0 σ (top), 0.5 σ (middle) and omit map mFo-DFc difference map (bottom) are shown for each peptide-HLA-DR1 structure. In each, peptide is shown as stick representation, colored with C=indicated inset, N=blue, O=red. 2mFo-DFc maps are presented as grey mesh. For omit map analysis, a two macro-cycle refinement including simulated annealing was performed using *phenix.refine* in the absence of peptide atoms. A resultant mFo-DFc map was calculated from the omit map refinement. For each omit map, positive difference map peaks (green mesh, +3.0 σ) and negative difference map peaks (red mesh, -3.0 σ) are shown overlayed with the final peptide co-ordinates which were absent in their calculation.

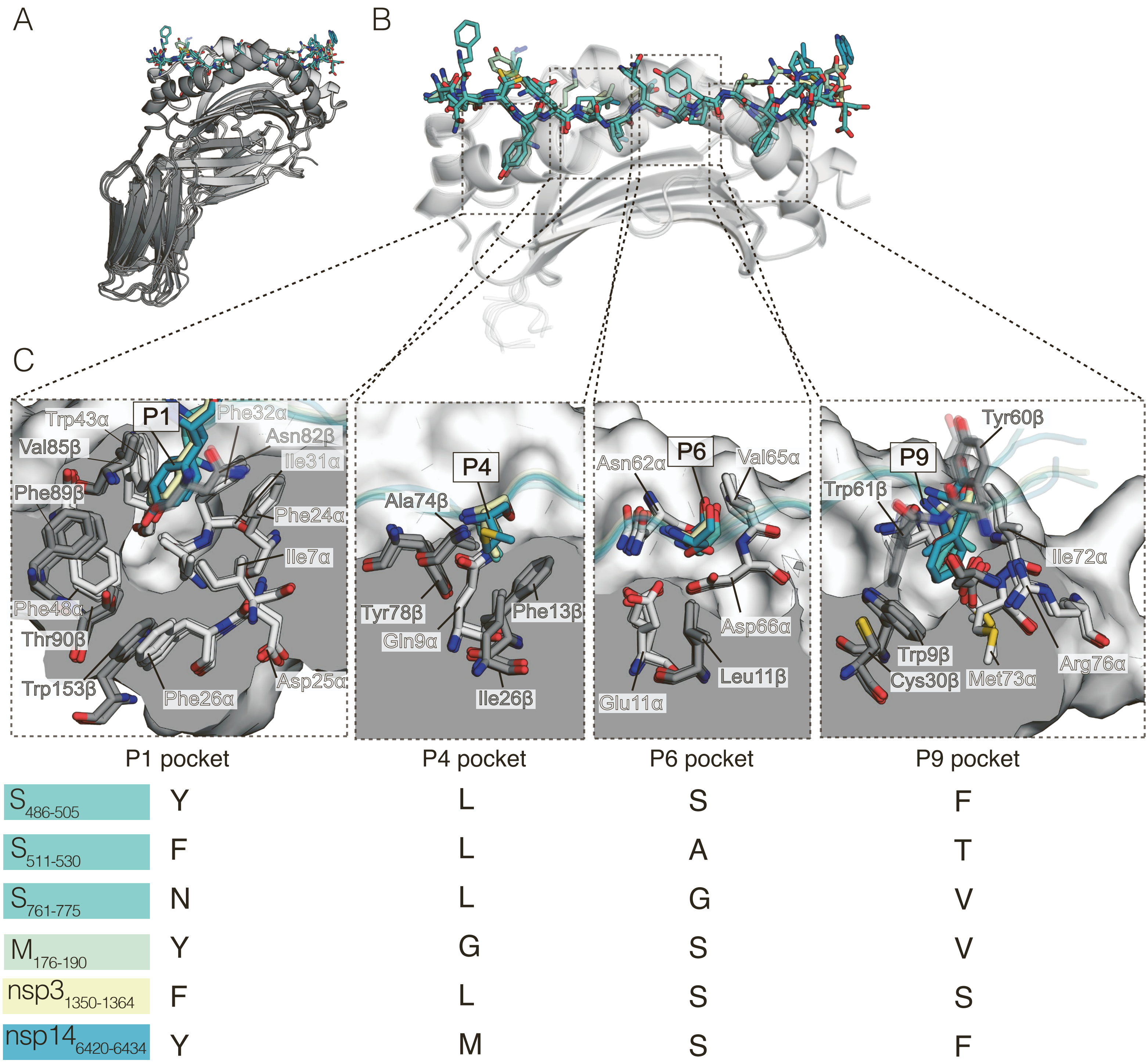
A
B
C
P1
Trp43α
Phe32α
Asn82β
Val85β
Ile31α
Phe89β
Phe24α
Ile7α
Phe48α
Thr90β
Trp153β
Phe26α
Asp25α
P4
Ala74β
Tyr78β
Gln9α
Phe13β
Ile26β
P6
Asn62α
Val65α
Asp66α
Leu11β
Glu11α
P9
Tyr60β
Trp61β
Ile72α
Trp9β
Cys30β
Met73α
Arg76α
P1 pocket
P4 pocket
P6 pocket
P9 pocket
S486-505 Y L S F
S511-530 F L A T
S761-775 N L G V
M176-190 Y G S V
nsp31350-1364 F L S S
nsp146420-6434 Y M S F

Figure S3 – Comparison of each SARS-CoV-2 peptide-HLA-DR1 structure. A) Structural alignment of the six solved peptide-HLA-DR1 complexes (HLA-DR1-$S_{486\text{-}505,}$ -$S_{511\text{-}530}$, -$S_{761\text{-}775}$, -$M_{176\text{-}190,,}$ -$nsp3_{1350\text{-}1364}$ and – $nsp14_{6420\text{-}6434}$). An overall view is shown of HLA-DR1 (grey, cartoon representation). Each peptide is shown as sticks and colored by atom (C= indicated inset matching to viral protein origin as in Fig 3, N=blue, O=red, S=yellow). **B)** A zoomed in view of the aligned peptide-HLA-DR1 binding groove for each structure as in A). **C)** Focused views of the P1, P4, P6 & P9 binding pockets of aligned peptide-HLA-DR1 structures. In each, the HLA-DR1 residues which form the binding pocket are shown as sticks (DRA: light grey, DRB: dark grey). Each peptide is shown (cartoon, semi-transparent) with the residue bound into each pocket show as opaque sticks. Residues which are in front of the bound peptide are shown semi-transparent for clarity (e.g. Asn82β). A surface cross-sectional view of the HLA-DR1 binding groove clipped in the z-plane at approximately the deepest point in the P1 pocket is also shown.

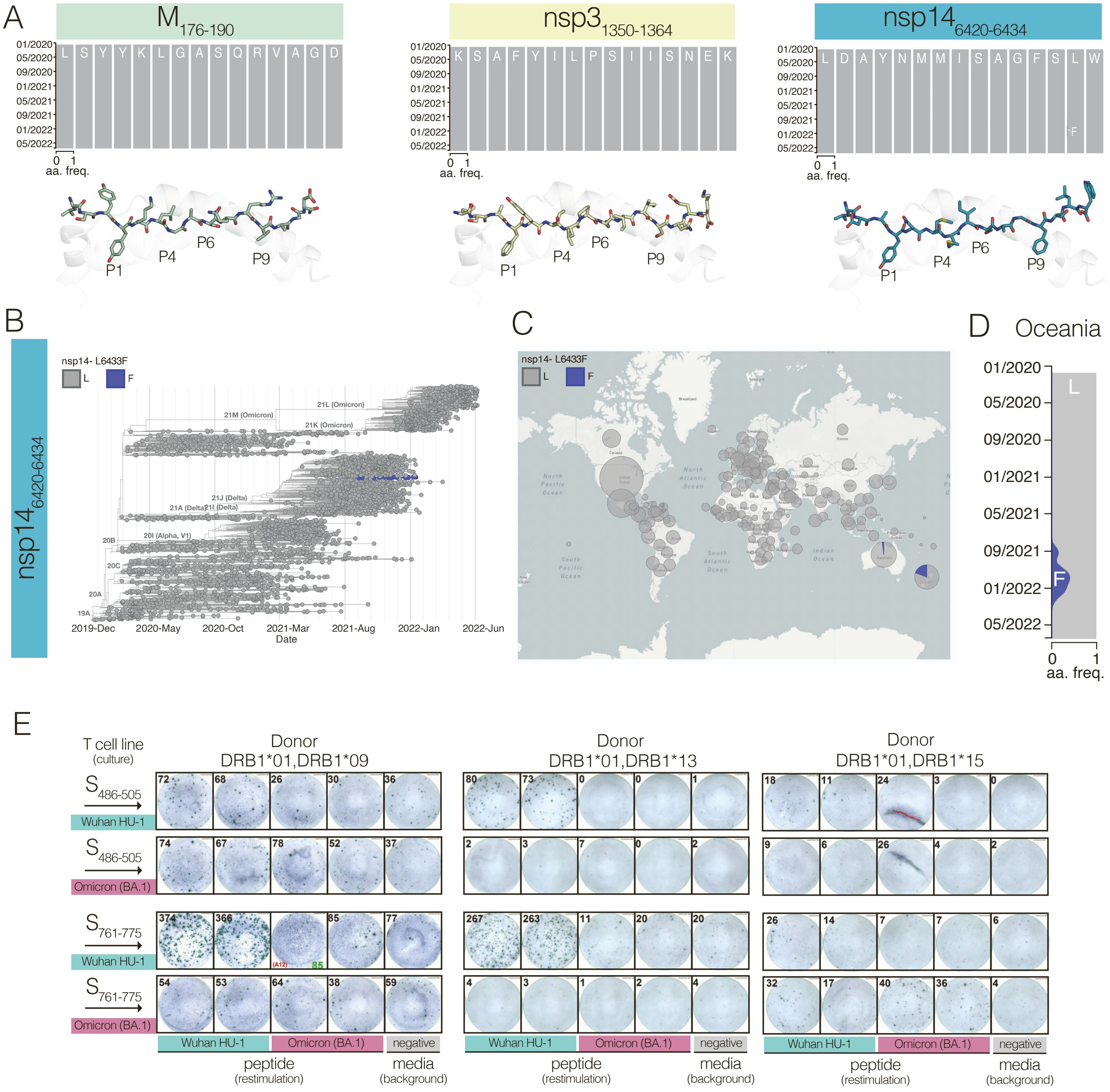
A
M176-190
nsp3 1350-1364
nsp14 6420-6434
aa. freq.
P1
P4
P6
P9
B
nsp14 6420-6434
nsp14- L6433F
21L (Omicron)
21M (Omicron)
21K (Omicron)
21J (Delta)
21A (Delta)
21I (Delta)
20I (Alpha, V1)
20B
20C
20A
19A
2019-Dec
2020-May
2020-Oct
2021-Mar
2021-Aug
2022-Jan
2022-Jun
Date
C
D
Oceania
E
T cell line
(culture)
Donor
DRB1*01,DRB1*09
DRB1*01,DRB1*13
DRB1*01,DRB1*15
S486-505
S761-775
Wuhan HU-1
Omicron (BA.1)
negative
peptide
(restimulation)
media
(background)

Figure S4 - Analysis of SARS-CoV-2 variant impact on epitope sequence and immunogenicity. A) Cumulative genotypic frequency plots of amino acid usage of peptide epitope residue positions over time (Jan 2020 – June 2022) in global viral genome sequences for non-Spike epitopes crystallized (GISAID database) - HLA-DR1-$M_{176-190}$ (left), -$nsp3_{1350-1364}$ (middle) and -$nsp14_{6420-6434}$ (right). For each, the reference amino acid (aa) usage (Wuhan HU-1 strain) is colored grey. No mutations associated with Omicron (BA.1) were observed in global data. Other mutations present in other lineages are colored blue. Plots generated using the Nextstrain ncov portal. **B)** Phylogeny tree of SARS-CoV-2 sequences in the GISAID global database highlighting a lineage of 21J (Delta) containing the nsp14-L6433F dimorphism contained within the HLA-DR1-$nsp14_{6420-6434}$ epitope. **C)** Geographical map highlighting the global distribution of the nsp14-L6433F dimorphism isolated to Oceania. Pie slices colored as indicated inset. **D)** Cumulative genotypic frequency plot of amino acid residue usage at position nsp14-L6433F over time (Jan 2020 – June 2022) in the GISAID Oceania dataset alone highlighting the nsp14-L6433F dimorphism occurrence over time within this population. **E)** IFN-γ ELISpot images of data described and plotted in Fig 5C) for all three HLA-$DR1^+$ donors. Donor DRB1*01, DRB1*13 is replicated from Fig 5D to allow ease of comparison with other donors. Peptides used in cultured T cell line are across rows and the variant peptides used for restimulation (overnight ELISpot assay) indicated in columns.

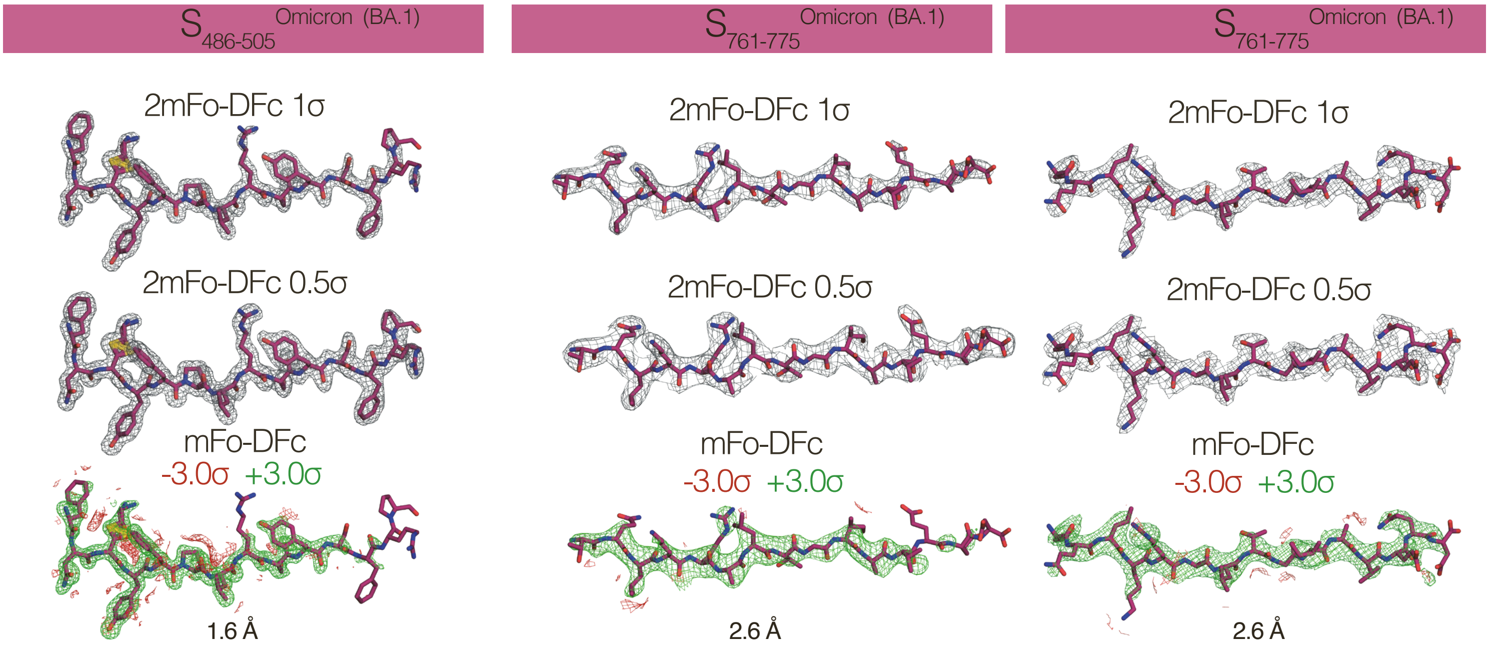

S486-505 Omicron (BA.1)
S761-775 Omicron (BA.1)
S761-775 Omicron (BA.1)
2mFo-DFc 1σ
2mFo-DFc 0.5σ
mFo-DFc
-3.0σ +3.0σ
1.6 Å
2.6 Å
2.6 Å

Figure S5 – Electron density maps and omit map analysis of HLA-DR1-S$_{486\text{-}505}$ $^{\text{Omicron (BA.1)}}$ and -S$_{761\text{-}775}$ $^{\text{Omicron (BA.1)}}$. Electron density and omit map analysis as described in FigS2 for HLA-DR1-S$_{486\text{-}505}$ $^{\text{Omicron (BA.1)}}$ (left) and -S$_{761\text{-}775}$ $^{\text{Omicron (BA.1)}}$ in the two registers observed in the asymmetric unit (middle & right). 2mFo-DFc contoured at 1.0 σ (top), 0.5 σ (middle) and omit map mFo-DFc difference map (bottom) are shown for each. In each, peptide is shown as stick representation, colored with C=indicated inset, N=blue, O=red. 2mFo-DFc maps are presented as grey mesh. For omit map analysis, a two macro-cycle refinement including simulated annealing was performed using *phenix.refine* in the absence of peptide atoms. A resultant mFo-DFc map was calculated from the omit map refinement. For each omit map, positive difference map peaks (green mesh, +3.0 σ) and negative difference map peaks (red mesh, -3.0 σ) are shown overlayed with the final peptide co-ordinates which were absent in their calculation.

	HLA-DR1-$S_{486-505}$	HLA-DR1-$S_{511-530}$	HLA-DR1-$S_{761-775}$	HLA-DR1-$M_{176-190}$	HLA-DR1-nsp$3_{1350-1364}$	HLA-DR1-nsp$14_{6420-6434}$	HLA-DR1-$S_{486-505}^{(BA.1)\ omicron}$	HLA-DR1-$S_{761-775}^{(BA.1\ Omicron)}$
Wavelength	0.9795	0.9795	0.9795	0.9795	0.9795	0.9795	0.9795	0.9795
Resolution range	42.43 - 1.84 (1.90 - 1.84)	52.47 - 1.42 (1.47 - 1.42)	91.56 - 2.54 (2.63 - 2.54)	47.29 - 2.26 (2.34 - 2.26)	55.11 - 2.20 (2.28 - 2.20)	59.80 - 1.64 (1.70 - 1.64)	50.16 - 1.64 (1.70 - 1.64)	48.67 - 2.60 (2.69 - 2.60)
Space group	P 2_1 2_1 2	P 6_5 2 2	C 2 2 2_1	C 2 2 2_1	P 2_1 2_1 2_1	P 6_5 2 2	P 2_1 2_1 2	P 3_2 2 1
Unit cell	89.29, 136.12, 41.18 90°, 90°, 90°	60.58, 60.58, 419.25 90°, 90°, 120°	113.02, 156.12, 164.85 90°, 90°, 90°	113.37, 156.77, 165.45 90°, 90°, 90°	60.23, 95.49, 202.43 90°, 90°, 90°	61.25, 61.25, 418.59 90°, 90°, 120°	91.03, 120.19, 40.88 90°, 90°, 90°	148.19, 148.19, 129.07 90°, 90°, 120°
Total reflections	580210 (60381)	3420039 (337712)	1721918 (174963)	946681 (92623)	1678731 (154626)	1271644 (126014)	754062 (76271)	1062657 (109285)
Unique reflections	44533 (4359)	88010 (8568)	48332 (4754)	69030 (6779)	60213 (5910)	58910 (5691)	55347 (5416)	50460 (4998)
Multiplicity	13.0 (13.9)	38.9 (39.4)	35.6 (36.8)	13.7 (13.7)	27.9 (26.2)	21.6 (22.1)	13.6 (14.1)	21.1 (21.8)
Completeness (%)	99.96 (99.98)	99.89 (99.63)	99.69 (99.22)	99.81 (98.96)	99.64 (98.89)	99.52 (98.07)	98.92 (97.87)	99.52 (99.09)
Mean I/sigma(I)	10.48 (0.80)	16.93 (0.73)	8.05 (0.88)	16.05 (0.50)	9.39 (0.86)	24.06 (1.08)	6.20 (0.59)	5.93 (0.56)
Wilson B-factor	36.33	20.77	53.04	62.92	44.49	32.77	19.44	55.03
R_{merge}	0.133 (3.046)	0.116 (3.341)	0.808 (8.588)	0.0996 (3.099)	0.345 (4.163)	0.0540 (2.341)	0.341 (3.678)	0.358 (2.637)
R_{meas}	0.138 (3.162)	0.117 (3.383)	0.820 (8.707)	0.104 (3.219)	0.352 (4.244)	0.0553 (2.396)	0.354 (3.815)	0.367 (2.699)
R_{pim}	0.0385 (0.842)	0.0187 (0.530)	0.137 (1.431)	0.0279 (0.867)	0.0663 (0.818)	0.0120 (0.502)	0.0949 (1.005)	0.0793 (0.571)
$CC_{1/2}$	0.999 (0.368)	1.000 (0.663)	0.994 (0.356)	0.998 (0.378)	0.998 (0.717)	0.999 (0.859)	0.995 (0.340)	0.992 (0.522)
CC*	1.000 (0.734)	1.000 (0.893)	0.999 (0.725)	1.000 (0.741)	1.000 (0.914)	1.000 (0.961)	0.999 (0.712)	0.998 (0.828)
Reflections used in refinement	44522 (4358)	87967 (8565)	48190 (4717)	68956 (6753)	60029 (5859)	58711 (5643)	55327 (5411)	50437 (5001)
Reflections used for R-free	2182 (217)	4405 (425)	2452 (219)	3406 (315)	2972 (280)	2865 (281)	2731 (283)	2554 (245)
R_{work}	0.193 (0.375)	0.187 (0.310)	0.195 (0.355)	0.198 (0.383)	0.207 (0.368)	0.194 (0.411)	0.182 (0.325)	0.192 (0.293)
R_{free}	0.231 (0.427)	0.209 (0.319)	0.239 (0.366)	0.240 (0.408)	0.240 (0.380)	0.226 (0.445)	0.207 (0.346)	0.237 (0.332)
CC_{work}	0.967 (0.685)	0.961 (0.797)	0.963 (0.673)	0.966 (0.546)	0.967 (0.822)	0.962 (0.883)	0.969 (0.657)	0.956 (0.658)
CC_{free}	0.967 (0.643)	0.963 (0.757)	0.955 (0.665)	0.948 (0.426)	0.954 (0.757)	0.963 (0.833)	0.965 (0.628)	0.931 (0.546)
Number of non-hydrogen atoms	3451	3608	9751	9655	6591	3447	3686	9714
- macromolecules	3207	3239	9546	9546	6363	3135	3228	9465
- ligands	48	82	40	20	32	84	133	98
- solvent	196	287	165	89	196	228	325	151
Protein residues	388	388	1162	1163	772	382	388	1156
RMS_{bonds}	0.016	0.015	0.004	0.005	0.006	0.012	0.005	0.002
RMS_{angles}	1.36	1.44	0.75	0.79	0.82	1.26	0.81	0.60
Ramachandran favored (%)	98.17	98.17	98.08	98.08	97.89	98.66	97.91	97.89

Ramachandran allowed (%)	1.83	1.83	1.92	1.83	2.11	1.34	2.09	2.11
Ramachandran outliers (%)	0.00	0.00	0.00	0.09	0.00	0.00	0.00	0.00
Rotamer outliers (%)	0.28	0.00	0.95	0.48	0.00	0.00	0.00	0.10
Clashscore	6.89	3.81	4.73	3.20	4.86	4.42	3.90	4.69
Average B-factor	40.93	30.06	56.11	74.35	62.96	46.47	25.80	65.65
- macromolecules	40.80	29.03	56.21	74.44	63.23	46.28	24.47	65.66
- ligands	47.19	48.90	64.86	83.85	71.48	53.04	42.15	83.45
- solvent	41.59	36.33	48.48	62.01	52.81	46.62	32.38	53.89
Number of TLS groups	10	5	36	33	19	5	10	34

Statistics for the highest-resolution shell are shown in parentheses.

Table S1 – Data collection and refinement statistics

	HLA-DR1-$S_{486-505}$	HLA-DR1-$S_{511-530}$	HLA-DR1-$S_{761-775}$	HLA-DR1-$M_{176-190}$	HLA-DR1-$nsp3_{1350-1364}$	HLA-DR1-$nsp14_{6420-6434}$	HLA-DR1-$S_{486-505}^{(BA.1)\ omicron}$	HLA-DR1-$S_{761-775}^{(BA.1\ Omicron)}$
Peptide sequence	FNCYFPLQSYGF QPTNGVGY	VVLSFELLHAPA TVCGPKKS	TQLNRALTGIAV EQD	LSYYKLGASQRV AGD	KSAFYILPSIISNE K	LDAYNMMISAGF SLW	FNCYFPLRSYSF RPTYGVGH	TQLKRALTGIAV EQD
Protein	S	S	S	M	nsp3	nsp14	S	S
Viral variant	Wuhan HU-1	Wuhan HU-1	Wuhan HU-1	Wuhan HU-1	Wuhan HU-1	Wuhan HU-1	Omicron (BA.1)	Omicron (BA.1)
Residues	S 486-505	S 511-530	S 761-775	M 167-181	nsp3<br><br>Orf1ab 1350-1364	nsp14<br><br>Orf1ab 6420-6434	S 486-505	S 761-775
Beamline	DLS i04	DLS i04	DLS i04	DLS i04	DLS i04	DLS i04	DLS i04	DLS i04
Proposal/visit	mx20147-39	mx20147-39	mx20147-44	mx20147-39	mx29502-3	mx29502-3	mx29990-2	mx29990-2
Crystallization conditions	M MES pH 6.0 20 % PEG1500	0.1 M MES pH 7.0, 25 % PEG8000, 0.2 M $(NH_4)_2SO4$	0.1 M MES pH 5.0, 20 % PEG1500	0.1 M MES pH 5.0 20 % PEG3350	0.1 M SPG pH 4.6 25 % PEG1500	0.1 M Sodium cacodylate pH 6.5, 25 % PEG8000, 0.2 M $(NH_4)_2SO4$	0.1 M Sodium cacodylate pH 6.0,25 % PEG4000, 0.2 M $(NH_4)_2SO4$	0.1 M Tris pH 7.0, 28 % PEG8000, 0.2 M $(NH_4)_2SO4$
Cryoprotectant	+ 10 % ethylene glycol	+ 10 % ethylene glycol	+ 10 % ethylene glycol	+ 10 % ethylene glycol	+ 10 % ethylene glycol	+ 10 % ethylene glycol	+ 10 % ethylene glycol	+ 10 % ethylene glycol
Drop size	3 µL	0.4 µL	3 µL	3 µL	0.4 µL	0.4 µL	0.4 µL	0.4 µL
Crystallization experiment	Hanging drop	Sitting drop	Hanging drop	Hanging drop	Sitting drop	Sitting drop	Sitting drop	Sitting drop
PHASER model (PDB Acc. No.)	6QZC	4X5W	4X5W	4X5W	3PDO	4X5W	HLA-DR1-$S_{486-505}$	HLA-DR1-$S_{761-775}$

Table S2 – Additional p-HLA-DR1 crystallization, data collection and refinement information
